# Supplementary material for: Trends in fertility intentions and contraceptive practices in the context of COVID-19 in sub-Saharan Africa: insights from four national and regional population-based cohorts
Source: BMJ Open. 2023 Jan 18;13(1):e062385. doi: 10.1136/bmjopen-2022-062385 (PMC9852736; doi:10.1136/bmjopen-2022-062385)
Supplement: Supplementary data [file bmjopen-2022-062385supp001.pdf]

APPENDIX 1

The following GEE model was used to test for differential trends in the use of any contraception between Phase 1 and Phase 2, by women's sociodemographic characteristics

$\text{Logit}\{E(y_{it})\}= \beta_n x_{nit} * t$, $y \sim \text{Bernoulli}$

Y_{it} represents the response for each subject i, measured at different time points (t=1,2).
$Xn=(X_1, X_2, X_3, \ldots, Xn$ represent sociodemographic variables
x_n represents each sociodemographic variable
βn represents the slope of the outcome for variable x_n

We used a logit function as the outcome y (contraception use) is binary with a Bernoulli distribution. We used unstructured-free estimation to account for within-subject correlation

Moreau C, *et al. BMJ Open* 2023; 13:e062385. doi: 10.1136/bmjopen-2022-062385

APPENDIX 2 Cross-sectional reproductive characteristics of women at Phase 1 and Phase 2, by urban and rural setting

	Urban Burkina Faso		Rural Burkina Faso		Urban Kenya		Rural Kenya		DRC-Kinshasa		Nigeria-Lagos	
	Phase 1 (n=)	Phase 2	Phase 1 (n=)	Phase 2	Phase 1 (n=)	Phase 2	Phase 1 (n=)	Phase 2	Phase 1 (n=2,611)	Phase 2	Phase 1 (n=1,469)	Phase 2
Intends to have a child in next year	18.6 (16.9, 20.6)	18.2 (16.8, 19.7)	11.9 (9.9, 14.3)	12.9 (10.8, 15.5)	8.4 (6.9, 10.1)	7.8 (6.5, 9.4)	6.3 (5.4, 7.4)	5.4 (4.7, 6.3)	12.2 (9.3, 15.8)	10.1 (7.8, 13.2)	16.8 (14.6, 19.2)	16.2 (14.6, 18.1)
In need of contraception	33.8 (31.9, 35.8)	35.5 (33.4, 37.7)	47.2 (43.4, 51.0)	47.1 (43.2, 51.0)	43.2 (40.9, 45.5)	43.7 (41.5, 45.9)	47.6 (46.0, 49.2)	46.4 (44.8, 48.1)	27.8 (24.7, 31.1)	29.2 (26.3, 32.2)	39.5 (36.6, 42.5)	40.4 (37.7, 43.2)
Contraceptive use, among women in need	61.5 (58.0, 64.9)	65.9 (62.8, 68.9)	39.4 (34.5, 44.5)	49.4 (44.3, 54.5)	79.1 (75.8, 82.0)	82.6 (79.8, 85.1)	68.2 (65.9, 70.5)	73.9 (71.6, 76.2)	71.8 (67.4, 75.9)	72.4 (68.2, 76.2)	63.4 (56.8, 69.5)	69.7 (63.7, 75.2)
Provider-dependent contraception, among contraceptive users	73.5 (69.5, 77.0)	68.7 (64.5, 72.7)	86.7 (81.3, 90.8)	82.1 (75.9, 87.0)	79.7 (75.8, 83.0)	80.1 (76.4, 83.3)	92.3 (90.8, 93.6)	89.1 (87.4, 90.6)	37.3 (29.5, 45.9)	35.6 (28.3, 43.5)	40.1 (33.7, 46.9)	35.2 (29.1, 41.8)
Currently pregnant	6 (5.2, 6.9)	6.1 (5.3, 7.0)	9.3 (8.1, 10.6)	8.1 (6.7, 9.6)	6.1 (5.1, 7.2)	4.2 (3.5, 5.1)	5.1 (4.5, 5.7)	5.5 (4.9, 6.2)	5.7 (4.5, 7.1)	5.2 (4.1, 6.5)	4.7 (3.7, 6.0)	4.7 (3.7, 5.9)
Current unintended pregnancy	1.6 (1.2,2.1)	1.2 (1.0,1.6)	3.1 (2.3,4.2)	2.3 (1.7,3.2)	2.2 (1.7,2.8)	1.5 (1.0,2.1)	2.3 (1.9,2.8)	2.3 (1.9,2.8)	2.6 (1.9,3.6)	2.8 (2.1,3.8)	1 (0.6-1.6)	1 (0.6.1.7)

Moreau C, *et al. BMJ Open* 2023; 13:e062385. doi: 10.1136/bmjopen-2022-062385

APPENDIX 3. Sociodemographic factors related to contraceptive adoption and discontinuation between Phases 1 and 2--results of multivariable logistic regression models, per setting

	Burkina Faso		Kenya		Kinshasa-DRC		Lagos-Nigeria	
	Adoption (n=972)	Discontinuation. (n=917)	Adoption (n=929)	Discontinuation. (n=2183)	Adoption (n=126)	Discontinuation. (n=321)	Adoption (n=156)	Discontinuation. (n=271)
	aOR (95% CI)							
Age								
15-24	ref	ref	ref	ref	ref	ref	ref	ref
25-34	1.3 (0.7-2.5)	**3.7 (1.1,12.0)**	0.7 (0.4-1.3)	1.2 (0.6-2.32)	0.5 (0.1-2.7)	2.1 (0.5-9.0)	-	1.0 (0.2-6.6)
35-49	1.0 (0.4-2.3)	2.9 (0.8-11.1	**0.4 (0.2-0.8)**	**2.0 (1.1-3.9)**	**0.1 (0.0-0.8)**	0.9 (0.2-4.7)	3.9 (0.3-51.3)	3.0 (0.4-24.7
Parity								
0-1	ref	ref	ref	ref	ref	ref	ref	ref
2-3	2.0 (1.0-3.9)	0.4 (0.1-1.6)	1.9 (1.0-3.8)	**0.4 (0.3-0.8)**	2.1 (0.10.4)	0.4 (0.2-1.6)	0.3 (0.0-2.3)	0.5 (0.1-1.9)
4+	1.6 (0.6-4.8)	0.9 (0.3-3.3)	1.4 (0.7-2.9)	**0.5 (0.2-0.9)**	1.3 (0.2-7.1)	0.6 (0.2-1.6)	0.6 (0.4-6.1)	0.3 (0.1-1.)
Residence								
Urban	1.3 (0.7-2.3)	0.8 (0.3-2.-2)	1.1 (0.7-1.6)	0.8 (0.5-1.3)	--	--	--	--
Rural	ref	ref	ref	ref	--	--	--	
Education								--
No schooling /primary	ref	ref	ref	ref	ref	ref	ref	ref
Secondary low	**6.0 (2.8-12.8)**	1.6 (0.6-4.5)	1.1 (0.5-2.8)	0.6 (0.1-2.2)	1.3 (0.2-7.1)	**0.4 (0.2-1.0)**	1.6 (0.4-6.1)	0.6 (0.2-2.0)
Secondary high	**22.6 (4.4-116.7)**	0.9 (0.3-3.3)	**2.0 (1.3-3.0)**	1.2 (0.8-1.7)	0.8 (0.1-7.5)	0.6 (0.2-2.7)	3.5 (0.8-15.4)	0.4 (0.1-2.0)
Food insecurity								
Yes	0.8 (0.3-2.3)	1.1 (0.3,3.6)	0.9 (0.6-1.5)	1.2 (0.6-2.1)	0.3 (0.1-1.3)	0.7 (0.3-1.5)	**-**	0.4 (0.0-3.8)
No	ref	ref	ref	ref	ref	ref	ref	ref
Wealth								
Poorest	ref	ref	ref	ref	ref	ref	ref	ref
Medium	0.7 (0.4-1.2)	0.7 (0.4,1.3)	1.4 (0.9-2.0)	1.0 (0.6-1.5)	0.8 (0.2-2.9)	0.9 (0.4-2.1)	0.7 (0.2-2.46	0.8 (0.3-2.2)
Richest	0.8 (0.4-1.5)	0.5 (0.2-1.6)	**2.0 (1.2-3.4)**	1.0 (0.6-1.7)	0.9 (0.2-3.1)	0.6 (0.2-1.4)	1.2 (0.4-3.9)	0.6 (0.2-2.1)

APPENDIX 4. Sociodemographic factors related to having an unintended pregnancy in Phase 2--results of multivariable logistic regression models, per setting

	Burkina Faso N=4018	Kenya N=5616	Kinshasa-DRC N=1489	Lagos-Nigeria N=836
Age				
15-24	ref	ref	ref	ref
25-34	**0.2 (0.1-0.5)**	0.7 (0.5-1.2)	0.4 (0.2-1.1)	0.3 (0.1,1.0)
35-49	**0.1 (0.0-0.4)**	**0.1 (0.1-0.3)**	**0.1 (0.0-0.3)**	**0.1 (0.0-0.3)**
Marital status				
Not in a cohabitating union	ref	ref	ref	ref
Married or in union	1.1 (0.4-3.1)	0.7 (0.45-1.2)	**2.4 (1.1-5.1)**	2.9 (0.4-21.6)
Parity				
0-1	ref	ref	ref	ref
2-3	1.6 (0.7-3.7)	1.2 (0.7-2.2)	**3.6 (1.4-8.9)**	1.3 (0.1-10.9)
4+	**5.1 (1.3-19.9)**	1.4 (0.6-3.0)	**3.7 (1.2-11.4)**	0.8 (0.0-21.2)
Residence				
Urban	0.9 (0.4-1.9)	0.9 (0.4-1.6)	--	--
Rural	ref	ref	--	--
Education				
No schooling /primary	ref	ref	ref	ref
Secondary low	1.6 (0.8-3.2)	0.4 (0.1-1.6)	0.5 (0.2-1.3)	0.6 (0.1-4.3)
Secondary high	0.6 (0.2-2.0)	**0.4 (0.2-0.7)**	0.6 (0.1-3.2)	0.3 (0.0-3.5)
Food insecurity				
Yes	1.6 (0.7-3.8)	1.3 (0.7-2.2)	0.9 (0.6-2.0)	-
No	ref	ref	ref	
Wealth				
Poorest	ref	ref	ref	ref
Medium	1.4 (0.8-2.7)	**0.6 (0.3-0.9)**	**0.4 (0.2-1.0)**	0.3 (0.0-2.1)
Richest	0.8 (0.3-2.0)	**0.4 (0.2-0.8)**	0.5 (0.2-1.2)	1.1 (0.2-5.1)
Contraception at phase 1				
None	ref	ref	ref	ref
Less effective (barrier/natural)	1.0 (0.3-2.8)	1.4 (0.8-2.7)	1.1 (0.6,5.1)	0.3 (0.0,1.4)
Effective (short-acting hormonal)	1.4 (0.7-2.9)	1.1 (0.6-1.9)	**1.8 (0.2-0.8)**	0.4 (0.0-5.3)
Highly effective (long-acting methods)	**0.1 (0.0-0.2)**	**0.4 (0.2-0.8)**	**0.1 (0.0-0.3)**	**0.1 (0.0-0.8)**

± Less effective=barrier or natural methods; effective= short-acting hormonal methods, including pills, injectables, patches, or rings; highly effective=IUDs, implants, sterilization

APPENDIX 5. Sociodemographic characteristics by sample and setting, weighted

	Burkina Faso			Kenya			Kinshasa-DRC			Lagos-Nigeria		
Overall	Phase 1[a]	Phase 2	Panel	Phase 1	Phase 2	Panel	Phase 1	Phase 2	Panel	Phase 1	Phase 2	Panel
Age												
15-19	21.6	20.9	20.9	21.6	22.3	21.9	22.2	23.2	22.3	14.0	15.8	13.4
20-24	17.3	18.4	17.4	17.6	17.4	17.7	20.9	20.4	21.4	14.0	12.6	12.8
25-29	14.7	15.0	14.8	16.3	15.5	16.3	16.3	15.2	16.0	15.5	16.0	15.7
30-34	15.6	15.2	16.1	15.4	15.6	15.1	12.3	13.4	12.3	17.7	16.1	18.1
35-39	12.7	12.9	12.6	11.8	11.6	11.8	10.7	10.8	10.7	17.8	16.8	18.4
40-44	10.9	10.9	11.1	9.5	9.6	9.4	10.4	10.2	10.2	12.7	12.8	13.0
45-49	7.2	6.9	7.1	7.8	8.1	7.9	7.2	6.8	7.2	8.3	10.0	8.5
Parity												
Nulliparous	24.3	22.7	23.2	28.3	29.2	28.5	41.9	41.4	41.7	35.2	36.5	32.9
1-2	24.8	24.9	24.9	30.9	30.1	29.9	28.1	28.7	28.7	29.5	28.8	30.4
3+	50.9	52.5	51.9	40.9	40.7	41.6	30.1	30.0	29.7	35.3	34.7	36.7
Residence												
Urban	22.8	22.3	22.9	30.2	30.4	30.5	100.0	100.0	100.0	100.0	100.0	100.0
Rural	77.2	77.7	77.1	69.8	69.6	69.5	--	0.0	0.0	0.0	0.0	0.0
Wealth Tertile												
Poorest	330	33.5	33.1	35.9	34.6	35.8	33.9	31.7	33.6	34.2	33.0	34.7
Middle	33.5	32.9	33.2	33.5	34.2	33.6	32.3	33.0	32.7	30.7	33.0	30.8
Richest	33.5	33.7	33.7	30.6	31.3	30.6	33.8	35.4	33.8	35.1	34.0	34.6
Loss of income last 12 months												
None		0.0	0.0		22.4	21.1		0.0	0.0		0.0	0.0
Complete		9.2	9.7		25.2	25.0		41.4	41.7		29.0	32.5
Partial		90.8	90.3		52.4	53.9		58.6	58.3		71.0	67.6
Food insecurity												
No		93.8	93.9		88.1	88.9		79.5	81.0		92.0	92.4
Yes		6.2	6.1		11.9	11.1		20.5	19.0		8.0	7.6
Education												
None/primary	77.6	75.7	77.7	49.7	47.5	49.2	7.8	6.7	7.8	11.8	11.5	12.1
Secondary low	16.4	17.3	16.1	2.1	1.6	2.0	72.9	75.1	72.9	51.2	52.8	51.3
Secondary high+	6.1	7.0	6.2	48.2	50.8	48.8	19.3	18.2	19.3	37.0	35.7	36.6
Contraception												
None	67.7	70.1	68.5	50.4	52.2	52.2	57.1	55.4	55.1	60.2	60.6	60.2
Less effective	7.8	5.7	5.8	9.7	7.2	7.2	31.3	32.7	32.3	27.1	25.8	25.6
Effective	10.3	9.9	10.4	20.5	19.2	19.6	4.3	4.2	10.4	6.2	6.1	6.7
Highly effective	14.0	14.3	15.3	19.4	21.4	21.2	7.3	7.8	15.3	6.5	7.5	7.5

± Less effective=barrier or natural methods; effective= short-acting hormonal methods, including pills, injectables, patches, or rings; highly effective=IUDs, implants, sterilization

Moreau C, *et al. BMJ Open* 2023; 13:e062385. doi: 10.1136/bmjopen-2022-062385
