# Supplementary material for: Trends in fertility intentions and contraceptive practices in the context of COVID-19 in sub-Saharan Africa: insights from four national and regional population-based cohorts
Source: BMJ Open. 2023 Jan 18;13(1):e062385. doi: 10.1136/bmjopen-2022-062385 (PMC9852736; doi:10.1136/bmjopen-2022-062385)
Supplement: Author's manuscript [file bmjopen-2022-062385.draft_revisions.pdf]

BMJ Open is committed to open peer review. As part of this commitment we make the peer review history of every article we publish publicly available.

When an article is published we post the peer reviewers' comments and the authors' responses online. We also post the versions of the paper that were used during peer review. These are the versions that the peer review comments apply to.

The versions of the paper that follow are the versions that were submitted during the peer review process. They are not the versions of record or the final published versions. They should not be cited or distributed as the published version of this manuscript.

BMJ Open is an open access journal and the full, final, typeset and author-corrected version of record of the manuscript is available on our site with no access controls, subscription charges or pay-per-view fees (http://bmjopen.bmj.com).

If you have any questions on BMJ Open's open peer review process please email info.bmjopen@bmj.com

BMJ Open

Trends in fertility intentions and contraceptive practices in the context of COVID-19 in sub-Saharan Africa: Insights from four national and regional population-based cohorts

Journal:	*BMJ Open*
Manuscript ID	bmjopen-2022-062385
Article Type:	Original research
Date Submitted by the Author:	09-Mar-2022
Complete List of Authors:	Moreau, Caroline; Johns Hopkins University Bloomberg School of Public Health, Department of Population Family and Reproductive Health; INSERM, Soins et Santé Primaire, CESP Centre for Research in Epidemiology and Population Health U1018<br>Karp, Celia; Johns Hopkins University Bloomberg School of Public Health<br>Wood, Shannon; Johns Hopkins University Bloomberg School of Public Health<br>Williams, Kelsey; Johns Hopkins University Bloomberg School of Public Health, Department of Population Family and Reproductive Health<br>Olaolorun, Funmi; University of Ibadan, Department of Community Medicine<br>Akilimali, Pierre; DRC<br>Guiella, Georges; Universite de Ouagadougou<br>Gichangi, Peter; International Centre for Reproductive Health Kenya<br>Zimmerman, Linnea; Johns Hopkins Bloomberg School of Public Health, Population Family and Reproductive Health<br>Anglewicz, Philip; Johns Hopkins University, Population, Family, and Reproductive Health
Keywords:	COVID-19, GYNAECOLOGY, REPRODUCTIVE MEDICINE

SCHOLARONE™
Manuscripts

I, the Submitting Author has the right to grant and does grant on behalf of all authors of the Work (as defined in the below author licence), an exclusive licence and/or a non-exclusive licence for contributions from authors who are: i) UK Crown employees; ii) where BMJ has agreed a CC-BY licence shall apply, and/or iii) in accordance with the terms applicable for US Federal Government officers or employees acting as part of their official duties; on a worldwide, perpetual, irrevocable, royalty-free basis to BMJ Publishing Group Ltd ("BMJ") its licensees and where the relevant Journal is co-owned by BMJ to the co-owners of the Journal, to publish the Work in this journal and any other BMJ products and to exploit all rights, as set out in our licence.

The Submitting Author accepts and understands that any supply made under these terms is made by BMJ to the Submitting Author unless you are acting as an employee on behalf of your employer or a postgraduate student of an affiliated institution which is paying any applicable article publishing charge ("APC") for Open Access articles. Where the Submitting Author wishes to make the Work available on an Open Access basis (and intends to pay the relevant APC), the terms of reuse of such Open Access shall be governed by a Creative Commons licence – details of these licences and which Creative Commons licence will apply to this Work are set out in our licence referred to above.

Other than as permitted in any relevant BMJ Author's Self Archiving Policies, I confirm this Work has not been accepted for publication elsewhere, is not being considered for publication elsewhere and does not duplicate material already published. I confirm all authors consent to publication of this Work and authorise the granting of this licence.

For peer review only

Trends in fertility intentions and contraceptive practices in the context of COVID-19 in sub-Saharan Africa: Insights from four national and regional population-based cohorts

Caroline Moreau[1,2]
Celia Karp[1]
Shannon N. Wood[1]
Kelsey Williams[1]
Funmilola OlaOlorun[3]
Pierre Akilimali[4]
Georges Guiella[5]
Peter Gichangi[6,7]
Linnea A. Zimmerman[1]
Philip A. Anglewicz[1]

[1] Department of Population Family and Reproductive Health, Johns Hopkins Bloomberg School of Public Health, Baltimore, Maryland, USA
[2] Soins et Santé Primaire, CESP Centre for Research in Epidemiology and Population Health U1018, Inserm, F-94805 Villejuif, France
3 College of Medicine, University of Ibadan, Ibadan, Nigeria
4 Kinshasa School of Public Health, Kinshasa, DRC.
5 Institut Supérieur des Sciences de la Population (ISSP/University of Ouagadougou), Ouagadougou, Burkina Faso
6 International Centre for Reproductive Health-Kenya, Nairobi, Kenya, Technical University of Mombasa;
7 Department of Public Health and Primary Care, Faculty of Medicine and Health Sciences, Ghent University, Belgium;

Corresponding author
Caroline Moreau, MD, PhD
Department of Population, Family and Reproductive Health
Johns Hopkins Bloomberg School of Public Health
615 N. Wolfe Street Rm. E4537 Baltimore, MD USA 21205
cmoreau2@jhu.edu

Word count: 3,584/5,000
Figures: 1
References: 23
Tables: 4 + 3 in Appendix

For peer review only

Significance Box

What we know?

- Despite significant healthcare disruptions, the impact of the pandemic on contraceptive behaviors and reproductive outcomes in sub-Saharan Africa remains largely unknown
- One study reports unexpected increases in contraceptive use in Burkina Faso and Kenya in the first six months of the pandemic but prolonged the epidemic and rising poverty is likely to further constrain women's access to family planning services in the region.

What are the new findings?

- This population-based longitudinal study, conducted in four geographies in Sub-Saharan Africa (Lagos-Nigeria, Burkina Faso Kinshasa-DRC, and Kenya) reveals no change in exposure to the risk of unintended pregnancy but a 5 to 9% rise in contraceptive coverage among women at risk in the first year of the pandemic in three study geographies.
- Unintended pregnancies were uncommon (1 to 2.8%) and unchanged since the start of the pandemic

What do the new findings imply?

- This study highlights the resiliency of African women in sustaining contraceptive practices amid the COVID-19 pandemic.
- Rising poverty and the potential diversion of international family planning funding call for continued monitoring of the impact of COVID-19 on sexual and reproductive health in the region.

For peer review only

ABSTRACT

Objectives: Studies conducted early in the COVID-19 pandemic suggested little impact on contraceptive behaviors in several sub-Saharan geographies. Initial results may mask widening disparities, with rising poverty, and changes to women's pregnancy desires and contraceptive use amid prolonged health service disruptions. We aimed to examine these trends in four African settings one year into the pandemic.

Methods: We used nationally and regionally representative longitudinal surveys from Burkina Faso, Kenya, Democratic Republic of Congo (Kinshasa), and Nigeria (Lagos) to evaluate changes in fertility preferences, contraceptive behaviors, and unintended pregnancies between the pre-COVID-19 period (November 2019-January 2020) and the COVID-19 era (November 2020-January 2021). Samples ranged from 1,469 women in Nigeria to 9,477 in Kenya. We described population-level and individual-level changes by socio-economic characteristics using generalized equation modeling. We used logistic regression models to identify factors related to contraceptive adoption and discontinuation and to the experience of an unintended pregnancy.

Results: At the population level, we found no change in women's exposure to unintended pregnancy risk but increases in contraceptive prevalence ranging from 5 to 9 percentage points in Burkina Faso, Kenya and Lagos. Reliance on prescriber-dependent methods dropped by two and four percentage points in Kenya and Burkina Faso, although these declines were not statistically significant. Between 1.0% and 2.8% of women across sites experienced an unintended pregnancy during COVID-19; with no significant change over time. Individual-level trajectories show contraceptive adoption was more common than discontinuation in Burkina Faso, Kenya, and Lagos, with little difference by sociodemographic characteristics. Women's COVID-related economic vulnerability was unrelated to unintended pregnancy across sites.

Conclusions:
This study highlights the resiliency of African women across diverse settings in sustaining contraceptive practices amid the COVID-19 pandemic. However, with rising poverty, there is continued need to monitor women's access to essential sexual and reproductive health services.

For peer review only

INTRODUCTION

The COVID-19 pandemic was projected to significantly alter fertility intentions, behaviors, and outcomes due to the global health and economic crises it induced [1–3]. This detrimental impact was expected to be far-reaching in sub-Saharan Africa [1], given pre-existing high unmet need [4]. Despite grim reports of family planning service disruptions during the first wave of COVID-19 infections and associated lockdown measures [1,2] and their anticipated effects on maternal health [1,2], initial empirical evidence from four sub-Saharan African countries, Burkina Faso, Democratic Republic of Congo, Kenya, and Nigeria, suggest little impact on women's use of contraception in the early stages of the pandemic [5]. In fact, the study reports a rise in contraceptive prevalence in rural Burkina Faso and across Kenya. These data are corroborated with findings further exploring changes in women's individual-level contraceptive behaviors in the early months of the pandemic in Burkina Faso and Kenya; specifically, these results indicate that more women adopted contraception than discontinued their methods between the end of 2019 and mid-2020 (13% Burkina Faso and 25% Kenya versus 5% Burkina Faso and 6% Kenya, respectively) [6].

While these initial studies were pivotal in reporting early pandemic trends, the longer term societal impacts of COVID-19, including rising poverty [7–9] and interruptions to schooling and employment [10,11], are likely to affect decisions regarding relationships and childbearing [11,12]. At the same time, growing economic disparities are likely to affect people's financial access and use of health services [7,11,12], potentially reducing their ability to achieve their reproductive goals. Furthermore, early impact estimates were drawn from phone-based surveys amid COVID-19 lockdowns, which may affect the interpretation of early study findings due to selection bias and data collection mode [5,6,13].

Against this backdrop, this study aimed to describe trends in women's fertility preferences, contraceptive behaviors, and pregnancy experiences in the context of COVID-19 pandemic across four sub-Saharan African countries/regions. We examined how these trends differed by sociodemographic characteristics and by COVID-19-related economic hardship. Finally, we examined factors related to individual-level changes in contraceptive behaviors and to experiencing an unintended pregnancy.

METHODS

Samples

We used two rounds of publicly available data (available at www.pmadata.org) collected in November - January 2019 (3-4 months before the pandemic) and one year later from nationally or regionally representative, population-based surveys. Surveys were implemented by the Performance Monitoring for Action (PMA) project among women aged 15-49 in four sub-Saharan African geographies: Burkina Faso, Kinshasa in the Democratic Republic of Congo, Kenya, and Lagos State in Nigeria. Women were identified using a multistage clustered sampling design, starting with the random selection of census enumeration areas (EAs) after stratifying by rural and urban area (where relevant), followed by the random selection of 35 households per EA. All women of reproductive age (15-49 years) from the selected households were eligible for inclusion. Sample sizes were determined to produce modern contraceptive prevalence rates (mCPRs) with a precision of +/- 3% at the national or regional level, leading to sample size variation by geography (Table 1).

For peer review only

Patient and Public involvement

This study is a population-based study that does not include patients. The public was not involved in this research.

Data collection

Pre-pandemic data (PMA's Phase 1 surveys) were collected face-to-face by trained resident interviewers before COVID-19 (November 2019-January 2020). Women who consented to be followed-up as part of PMA's panel design were re-interviewed face-to-face, one year later (PMA's Phase 2 surveys), during the pandemic (November 2020-January 2021). Replacement households from the EA were also included in Phase 2 to account for attrition at the household level (projected at 15%). The survey design allows for longitudinal analysis to examine individual-level changes since the onset of COVID-19 and for annual cross-sectional analysis to track national or regional level indicators (including household replacements). Follow-up rates between Phase 1 and Phase 2 surveys ranged from 73% in Kenya to 79% in Burkina Faso (Table 1). No differences between Phase 1 and Phase 2 cross-sectional sample characteristics were observed; differential lost to follow-up in the panel sample was accounted for by applying post-stratification weights (more details available at www.pmadata.org).

Table 1. Number of women aged 15 to 49 years included in each PMA survey and follow-up rate, by setting

Country/Site	Phase 1-Baseline	Phase 2 cross-sectional-including household replacement	Phase 2 panel-no replacement sample
Burkina Faso	6,590	6,362	5,207 (79%)
Kenya	9,477	9,310	6,932 (73%)
Kinshasa, DRC	2,611	2,368	1,967 (75%)
Lagos, Nigeria	1,469	1,481	1,088 (74%)

DRC=Democratic Republic of Congo.

Follow-up rate reported as % of all baseline women who successfully completed the Phase 2 survey

For peer review only

Ethical Approval

PMA surveys received approval from ethical committees in each geography (Burkina Faso: Comité d'Ethique Institutionnel Pour La Recherche en Santé, Ministère de l'Enseignement Supérieur, de la Recherche Scientifique et de l'Innovation; Democratic Republic of the Congo: University of Kinshasa School of Public Health; Kenya: Kenyatta National Hospital—University of Nairobi Ethics Research Committee; Nigeria: Lagos State University Teaching Hospital Health Research Ethics Committee). PMA also received approval from the Institutional review board of the Johns Hopkins School of Public Health (IRB00014702). Public health preventive measures (i.e., social distancing, mask-wearing) were instituted in Phase 2 to prevent COVID-19 transmission during data collection.

Measures

At each survey, women were asked about their current pregnancy status, whether the pregnancy was wanted or happened at the right time, future fertility intentions, and current contraceptive behaviors. This information was operationalized as indicators used for population-level comparisons (cross-sectional measures) and for individual-level trends analysis (longitudinal measures).

Cross-sectional measures

We explored six measures reflecting women's sexual and reproductive health experiences before and during the pandemic. *Women's fertility intentions for the next year* distinguished women who intended to have a child in the next year from those who wanted no more children or wanted to delay childbearing for more than one year. *Exposure to the risk of unintended pregnancy* (yes/no) classified women who had sexual intercourse in the last 12 months, were fecund, and were not pregnant or trying to have a child in the next year as being "at risk". *Use of contraception among women at risk of unintended pregnancy* (yes/no) classified women who were using barrier and natural methods, pills, injectables, patches, rings, implants, IUDs, and sterilization as using contraception. *Use of a provider-dependent method among contraceptive users* (yes/no) classified women using methods requiring interaction with a healthcare provider (i.e., pills, injectables, implants, IUDs, sterilization) as provider-dependent method users. We also considered a three-category measure of contraceptive method types, grouping contraceptive users according to the effectiveness of their contraceptive method—users of *less effective* methods (i.e., barrier or natural methods), users of *effective* methods (i.e., short-acting hormonal methods, including pills, injectables, patches, or rings), and users of *highly effective* methods (i.e., implants, IUDs, or sterilization). *Pregnancy status* identified women who were pregnant at the time of each survey (yes/no), while an indicator of *unintended* (yes/no) identified women who were pregnant and indicated they had not wanted to become pregnant or wanted to become pregnant later.

Longitudinal measures

We also explored individual-level changes in women's *exposure to risk of unintended pregnancy*, *contraceptive use*, and *experience of an unintended pregnancy* (reported as wanted later or not at all). Changes in women's exposure to unintended pregnancy risk across the two surveys were categorized into four categories: 1) *No risk*—never at risk before or during COVID-19; 2) *Acquired risk*—newly at risk during COVID-19 (no risk pre-pandemic); 3) *Ceased risk*—no longer at risk during COVID-19 (at risk pre-pandemic); 4) *Sustained risk*—at risk before and during COVID-19. Likewise, we defined a four-category variable describing trends in any contraceptive use: 1)

For peer review only

Continued non-use—non-use before or during COVID-19; 2) *Adoption*— during COVID-19 (non-use pre-pandemic); 3) *Discontinuation*—non-use during COVID-19 following use pre-pandemic; 4) *Continued use*—use before and during COVID-19. Finally, we considered the three-category indicator of contraceptive effectiveness (less effective/effective/highly effective) and a binary indicator of unintended pregnancy (yes/no) during Phase 2 (i.e., at the time of the COVID-19 survey), as previously defined.

We considered several sociodemographic and COVID-19-related factors that could impact women's use of contraception. Specifically, we assessed age, parity, marital status (in union/ not in union), education, household wealth in tertiles, and residence (urban/rural). During COVID-19, we also considered two indicators of household economic vulnerability: loss of income in the last 12 months (categorical—none, partial, complete), and food insecurity (i.e., not being able to eat for 24 hours due to lack of food since the beginning of the COVID-19 pandemic).

Analysis

We first examined population-level trends in reproductive health indicators comparing Phase 1 and Phase 2 cross sectional samples across our six indicators (proportion who intend to have a child in the next year, proportion at risk of unintended pregnancy, proportion using contraception among women who were at risk of unintended pregnancy, proportion using provider-dependent methods among users, proportion pregnant, and proportion experiencing an unintended pregnancy. Statistically significant differences were determined based on non-overlapping confidence intervals between Phase 1 and Phase 2 estimates.

Next, we conducted individual level analysis, focusing on the sample of women completing both surveys. We evaluated changes in exposure to unintended pregnancy risk and changes in contraceptive behaviors (among women at risk at both time points). We used generalized estimation equation models (GEE) to test for differential trends in the use of any contraception between Phase 1 and Phase 2, by women's sociodemographic characteristics (measured at Phase 1) and their experience of economic instability (measured at Phase 2). Next, we examined the contraceptive method mix of contraceptive adopters and the methods women discontinued between the two surveys. We conducted three multivariable logistic regression models to identify factors related to 1) contraceptive adoption among women who were not using contraception pre-pandemic, 2) contraceptive discontinuation among women who were using a method pre-pandemic, and 3) the experience of an unintended pregnancy during the pandemic.

All analyses applied survey and post-stratification weights to account for the complex survey design, clustering of women within EAs and differential loss-to-follow-up in the case of the longitudinal sample. All analyses were conducted in Stata 16.1 with statistical significance set a priori at $p<0.05$.

RESULTS

The characteristics of pre-pandemic study samples by setting are presented in Table 2. By design, all women in Kinshasa and Lagos were living in urban areas, while this was the case for 22.8% and 30.2% of the samples in Burkina Faso and Kenya, respectively. The mean age of women ranged from 28.3 years in Kinshasa to 31.0 years in Lagos. Most women across settings were in

For peer review only

union, except in Kinshasa, where this was the case of 42.7% of respondents. Women in Burkina Faso had the highest parity (50.9% with three or more children), followed by Kenya (40.9% with three or more children), while lower parities were observed in Kinshasa and Lagos, where 41.9% and 35.2% of women, respectively, had no children. Educational attainment varied substantially across settings, as 77.6% of women in Burkina Faso had no schooling or a primary level of education, with this proportion dropping to 49.7% in Kenya and to 7.8% and 11.8% in Kinshasa and Lagos, respectively. Contraceptive practices also varied, with non-use ranging from 52.2% in Kenya to 70.1% in Burkina Faso. Reliance on highly effective methods was greatest in Kenya (21.4%) and Burkina Faso (14.3%), while use of less effective barrier and natural methods was more common in Kinshasa (32.7%) and Lagos (25.8%).

Table 2. Phase 1 sociodemographic characteristics, by setting

		Burkina Faso (n=6,590)	Kenya (n=9,477)	Kinshasa-DRC (n=2,611)	Lagos-Nigeria (n=1,469)
Age (mean)		28.9	28.8	28.3	31.0
		Column %			
Age	15-19	21.6	21.6	22.2	14.0
	20-24	17.3	17.6	20.9	14.0
	25-29	14.7	16.3	16.3	15.5
	30-34	15.6	15.4	12.3	17.7
	35-39	12.7	11.8	10.7	17.8
	40-44	10.9	9.5	10.4	12.7
	45-49	7.2	7.8	7.2	8.3
Union status	In union†	75.8	59.2	42.7	60.9
	Not in union	24.2	40.8	57.3	39.1
Parity	Nulliparous	24.3	28.3	41.9	35.2
	1-2 children	24.8	30.9	28.1	29.5
	3+ children	50.9	40.9	30.1	35.3
Residence	Urban	22.8	30.2	100.0	100.0
	Rural	77.2	69.8	--	--
Education	No schooling /primary	77.6	49.7	7.8	11.8
	Secondary low	16.4	2.1	72.9	51.2
	Secondary high or more	6.1	48.2	19.3	37.0
Contraception±	None	70.1	52.2	55.4	60.6
	Less effective	5.7	7.2	32.7	25.8
	Effective	9.9	19.2	4.2	6.1
	Highly effective	14.3	21.4	7.8	7.5

†In union defined as married or living with a partner as if married

± Less effective=barrier or natural methods; effective= short-acting hormonal methods, including pills, injectables, patches, or rings; highly effective=IUDs, implants, sterilization

For peer review only

At the population level, 6.2% to 16.8% of women intended a birth in the next 12 months across geographies, with no significant change during the pandemic (Table 3). Accounting for past-year sexual activity, fertility intentions, and fecundity, 27.8% to 46.3% of women were exposed to the risk of unintended pregnancy, with the highest proportion observed in Burkina Faso. Exposure to unintended pregnancy risk did not vary over time in any setting.

Contraceptive use (any method) varied widely by setting and was lowest in Burkina Faso and highest in Kenya and Kinshasa. During the pandemic, contraceptive use rose significantly by nine percentage points in Burkina Faso and five percentage points in Kenya. Contraceptive use also rose by six percentage points in Lagos (although confidence intervals were overlapping), while it remained stable in Kinshasa. The proportion of contraceptive users relying on provider-dependent methods ranged from 35.2% in Lagos to 88.3% in Kenya. This proportion remained stable in Kinshasa and Lagos and declines were non-significant in Burkina Faso and in Kenya (overlapping confidence intervals) (Table 3).

Between 5.1% women in Kenya and 8.5% of women in Burkina Faso were pregnant at the time of the survey with no change over time. Finally, between 1.0% and 2.8% of women across sites had an unintended pregnant at the time of the Phase 2 survey, this proportion remaining stable over time in all sites (Table 3).

For peer review only

Table 3. Cross-sectional reproductive characteristics of women at Phase 1 and Phase 2, by setting

	Burkina Faso		Kenya		DRC-Kinshasa		Nigeria-Lagos	
	Phase 1 (n=6,590)	Phase 2 (n=6,362)	Phase 1 (n=9,477)	Phase 2 (n=9,310)	Phase 1 (n=2,611)	Phase 2 (n=2,368)	Phase 1 (n=1,469)	Phase 2 (n=1,481)
	% (95% CI)							
Intends to have a child in next year	13.4 (11.8, 15.3)	14.1 (12.3, 16.1)	6.9 (6.1, 7·8)	6.2 (5.5, 6.9)	12.2 (9.3, 15.8)	10.1 (7.8, 13.2)	16.8 (14.6, 19.2)	16.2 (14.6, 18.1)
In need of contraception	44.1 (41.1, 47.2)	44.5 (41.4, 47.4)	46.3 (44.9, 47.6)	45.6 (44.3, 46.9)	27.8 (24.7, 31.1)	29.2 (26.3, 32.2)	39.5 (36.6, 42.5)	40.4 (37.7, 43.2)
Contraceptive use, among women in need	43.2 (39.0, 47.6)	52.3 (48.0, 56.6)	71.3 (69.4, 73.2)	76.5 (74.6, 78.2)	71.8 (67.4, 75.9)	72.4 (68.2, 76.2)	63.4 (56.8, 69.5)	69.7 (63.7, 75.2)
Provider-dependent contraception, among contraceptive users	83.4 (79.3, 86.9)	79.1 (74.3, 83.1)	88.3 (86.7, 89.8)	86.3 (84.6, 87.8)	37.3 (29.5, 45.9)	35.6 (28.3, 43.5)	40.1 (33.7, 46.9)	35.2 (29.1, 41.8)
Currently pregnant	8.5 (7.6, 9.6)	7.6 (6.6, 8.4)	5.4 (4.9, 5.9)	5.1 (4.6, 5.7)	5.7 (4.5, 7.1)	5.2 (4.1, 6.5)	4.7 (3.7, 6.0)	4.7 (3.7, 5.9)
Current unintended pregnancy	2.8 (2.1,3.7)	2.1 (1.6,2.7)	2.3 (2.0,2.7)	2.1 (1.7,2.4)	2.6 (1.9,3.6)	2.8 (2.1,3.8)	1.0 (0.6-1.6)	1.0 (0.6.1.7)

For peer review only

Assessment of individual-level trajectories of women who responded to both surveys indicated that changes in exposure to unintended pregnancy risk among all women ranged from 16.9% in Kinshasa to 31.5% in Burkina Faso (Figure 1). Half of changes involved new exposure to risk, while the other half led to cessation of exposure to risk during the pandemic. Among all women, the proportion at risk of unintended pregnancy at both time points (i.e., sustained risk) ranged from 20.5% in Kinshasa to 42.2% in Kenya. Among women with sustained risk, change in contraceptive status varied from 19.4% in Kenya to 25.2% in Burkina Faso. Contraceptive adoption was more common than discontinuation in Burkina Faso (16.9% versus 8.3%, respectively), Kenya (12.6% versus 6.8%, respectively) and Lagos (15.2% versus 8.7%, respectively) but equivalent in Kinshasa (11.5% versus 12.6%, respectively). Among women who changed contraceptive status, most adopted or discontinued less effective barrier and natural methods in Kinshasa (66.9% and 68.5%, respectively) and Lagos (77.8% and 74.1%, respectively), while most adopted or discontinued effective short-acting methods in Burkina Faso (45.1% and 44.7%, respectively) and Kenya (47.8% and 54.2%, respectively). Highly effective long-acting method contributed the least to adoption and discontinuation in Lagos (6.0% and 2.7%, respectively) and accounted for 27.0% of adoption and 39.5% of discontinuation in Burkina Faso. Patterns of change reflect differences in contraceptive method mix among women at risk before COVID-19, as 65.5% and 73.2% of contraceptive users relied on less effective methods in Kinshasa and Lagos, respectively, versus 15% in Kenya and 19% in Burkina Faso. Conversely, 44.7% of women at risk in Kenya and 47.8% in Burkina Faso relied on highly effective methods pre-pandemic versus 17.4% in Kinshasa and 19.0% in Lagos.

While results from GEE models identified few statistically significant interactions between time and women's characteristics, signaling limited differences in contraceptive trends by sociodemographic characteristics, some patterns emerged (Table 4). For example, increases in the odds of contraceptive use during COVID-19 relative to pre-pandemic were more pronounced among women in their mid-twenties compared to teenagers or women over 40 years in all sites but Kinshasa. Likewise, increases in the odds of contraceptive use were elevated among parous women but not among nulliparous women in Kenya. In Lagos, increases in the odds of contraceptive use were greater among women with 1-2 children relative to those who had more children. Finally, wealthiest women in Lagos experienced greater increases in the odds of contraceptive use relative to women in the medium wealth category in Lagos. Conversely, there were no differential trends by education, recent loss of income, or recent experience of food insecurity.

For peer review only

Table 4. Differential trends in contraceptive use by women's sociodemographic characteristics

	Burkina Faso (n=5,207)	Kenya (n=6,932)	DRC-Kinshasa (n=1,967)	Nigeria-Lagos (n=1,088)
	RRR (95% CI)			
Overall	1.4 (1.2, 1.6)	1.3 (1.2, 1.5)	0.9 (0.7, 1.2)	1.3 (1.1, 1.6)
Age#year interaction	p=0.06	p=0.24	p=0.13	p=0.08
15-19	1.3 (0.8, 2.2)	1.4 (0.7, 2.8)	0.4 (0.1,2.6)	-
20-24	1.4 (1.1, 1.8)	1.3 (1.0, 1.7)	1.5 (0.6,3.6)	1.1 (0.5, 2.2)
25-29	1.7 (1.2, 2.3)	1.5 (1.2, 1.9)	1.2 (0.7,2.2)	2.9 (1.5, 5.6)
30-34	1.2 (0.8, 1.6)	1.4 (1.2, 1.6)	0.4 (0.2,0.7)	1.7 (1.0, 3.1)
35-39	1.6 (1.2, 2.3)	1.5 (1.2, 1.8)	1.1 (0.6,1.8)	1.0 (0.7, 1.4)
40-44	1.5 (1.0, 2.1	1.1 (0.9, 1.3)	0.9 (0.6,1.4)	1.2 (0.7, 2.1)
45-49	0.8 (0.7, 1.1)	1.1 (0.9, 1.4)	1.0 (0.6,1.7)	1.1 (0.6, 2.0)
Parity#year interaction	p=0.34	p=0.08	p=0.88	p=0.004
Nulliparous	-	0.7 (0.2, 2.1)	3.3 (0.2, 55.9)	-
1-2	1.4 (1.1, 1.8)	1.5 (1.2, 1.8)	0.8 (0.5, 1.3)	2.1 (1.4, 23.0)
3+	1.4 (1.2, 1.6)	1.3 (1.1, 1.4)	1.0 (0.7, 1.2)	1.1 (0.8, 1.4)
Residence#year interaction	p=0.32	p=0.54		
Urban	1.3 (1.1, 1.4)	1.2 (1.0, 1.5)	--	--
Rural	1.4 (1.2, 1.6)	1.3 (1.2, 1.5)	--	--
Wealth#year interaction	p=0.57	p=0.61	p=0.42	p=0.08
Poorest	1.5 (1.2, 2.0)	1.4 (1.2, 1.6)	0.9 (0.5, 1.4)	1.5 (0.9, 2.3)
Medium	1.3 (1.0, 1.6)	1.3 (1.1, 1.6)	1.1 (0.7, 1.8)	1.1 (0.7, 1.6)
Richest	1.3 (0.9, 1.3)	1.2 (0.9, 1.5)	1.1 (0.7, 1.8)	2.0 (1.3, 2.9)
Loss of income last 12 months	p=0.30	p=0.48	p=0.71	p=0.09
None	-	1.2 (1.0, 1.4)	-	-
Partial	1.4 (1.2, 1.6)	1.4 (1.2, 1.5)	0.9 (0.7,1.3)	1.4 (1.1, 1.9)
Complete	1.5 (1.0, 2.3)	1.3 (1.1, 1.5)	0.9 (0.7, 1.2)	1.2 (0.8, 1.7)
Food insecurity	p=0.24	p=0.33	p=0.85	p=0.10
No	1.4 (1.2, 1.5)	1.3 (1.2, 1.4)	1.0 (0.7, 1.3)	1.4 (1.1, 1.7)
Yes	1.8 (1.1, 2.9)	1.5 (1.2, 1.9)	0.7(0.5, 1.1)	0.8 (0.6, 1.1)
Education	p=0.29	p=0.87	p=0.26	p=0.22
No schooling/primary	1.4 (1.2, 1.6)	1.3 (1.2, 1.4)	0.6 (0.3, 1.3)	1.2 (0.7, 2.0)
Secondary low	1.3 (0.8, 2.0)	1.2 (0.6, 2.4)	1.0 (0.8, 1.3)	1.2 (0.9, 1.7)
Secondary high or higher	0.7 (0.3,1.3)	1.3 (1.1,1.6)	0.7 (0.4,1.2)	1.9 (1.4,2.6)

For peer review only

Few factors were related to contraceptive adoption among women who were not using a method pre-pandemic and to contraceptive discontinuation among those using contraception pre-pandemic (Appendix 1). The odds of adopting contraception were higher among women with secondary education or higher in Burkina Faso and Kenya, relative to less educated women, and among women in the richest tertile, relative to the poorest women in Kenya. Conversely the oldest women (35-49 years) were less likely to adopt a method in Kinshasa and Kenya and, more likely to discontinue their method in Kenya. Discontinuation was also more common among parous women compared to nulliparous women in Kenya. Finally, women with lower secondary education in Kinshasa were less likely to discontinue their method than women with no schooling or primary education.

Finally, factors associated with characterizing an unintended pregnancy during COVID among women who had sexual intercourse in the last 12 months are reported in Appendix 2. Older women (35 years or older) and women using long-acting contraception were less likely to experience an unintended pregnancy across sites. Women with higher education and from wealthier households were less likely to experience an unintended pregnancy in Kenya, while women of higher parity were more likely to experience unintended pregnancies in Burkina Faso and Kinshasa. COVID-related economic vulnerability was unrelated to unintended pregnancy across sites.

DISCUSSION

This study confirms women's resiliency in the use of contraception to prevent unwanted pregnancies across a diversity of sub-Saharan contexts, despite the profound economic and social disruptions caused by the COVID-19 pandemic [7–12]. While we found sustained need for contraception, we also saw an increase in contraceptive coverage in three of the four geographies studied. Within Burkina Faso and Kenya, increases in contraceptive protection corresponded with a non-significant decrease in the use of methods requiring a prescription; but most women still used effective or highly effective methods, including women who adopted methods during the pandemic. These contraceptive dynamics coincide with the absence of significant changes in pregnancy rates or unintended pregnancy rates across sites.

These results challenge initial forecasts of rising unintended pregnancies due to service disruptions for sexual and reproductive health services in LMICs [1]. The absence of negative population-level effects of the pandemic on contraceptive behaviors and reproductive outcomes, noted in a previous study focusing on the early months of the pandemic [5,6], are surprisingly reassuring and suggest several potential explanations [14].

First, while disruptions to sexual and reproductive health (SRH) services were reported in the early stage of the pandemic [3,14], they may have been limited and short-lived [15], as suggested in several studies reporting rapid post-lockdown rebounds in service provision [14–16]. Further, it is possible that the quality of facility-based registers, including clients' family planning records, were impacted due to understaffing amid COVID-19-related disruptions and, thus, early pandemic provision of care was underreported [17].

For peer review only - http://bmjopen.bmj.com/site/about/guidelines.xhtml

At the same time, the expansion or continued use of high impact practices in family planning [18], such as community health workers, mobile outreach, supply chain management, and digital health initiatives, may also have compensated for facility-based disruptions to care. For example, the expansion of community health workers to deliver family planning services resulted in a 2.5 percent increase in the number of family planning clients in rural Kenya in the summer of 2020 [19]. These initiatives were encouraged by Kenyan government who issued guidelines to support "Continuity of Reproductive, Maternal, Newborn and Family Planning Care and Services in the Background of COVID-19 Pandemic" in the month following the first reported case of COVID-19 [20].

A third explanation lies in the resilience of women in intensifying their efforts or finding alternative solutions to access contraception and self-manage their contraceptive needs. This hypothesis is plausible given that our initial investigation into family planning amid COVID-19, conducted between May and July of 2020, found that only 3.8-14.4% of women at risk of unintended pregnancy were not using contraception for COVID-19-related reasons in Burkina Faso and Kenya; these women primarily cited fear of COVID-19 transmission [6]. Approximately six months later, the data collected via PMA surveys between November 2020 and January 2021 included in the present analysis reveal 9 to 35% reported difficulties accessing any health service in the last year, however 89-93% were ultimately successful in obtaining services (www.pmadata.org). Despite access-related difficulties, our data suggest that women were still able to obtain contraceptive methods, signaling the prioritization of women's SRH services amidst competing health, economic, and safety needs.

These overall trends remain encouraging, but a critical concern over widening social disparities in family planning access may still arise in the face of increasing economic hardships [7,8]. Our findings provide no evidence of adverse SRH outcomes among women who report food insecurity, or those who experienced substantial loss of income since the start of the COVID-19 pandemic. Recent policy initiatives towards universal access to primary healthcare, such as the implementation of free access to contraception in Burkina Faso in 2019 [21], are likely to have mitigated the economic impact of COVID-19, but the affordability of contraception remains a prominent concern for women experiencing economic hardship [9].

Our results should be considered with several limitations in mind. Specifically, the loss to follow-up rate ranged from 21-27% across geographies, which is likely to affect the reported estimates of change. However, the use of replacement households in Phase 2 of data collection was designed to minimize impacts to population-level estimates. Further, post-stratification weights were used in panel analysis to limit the potential for attrition biases; stable estimates of sociodemographic distributions across samples were observed (Appendix 3). A second limitation is our measure of change using two time points, which prevents a more comprehensive assessment of changes over the full time-period, including potential gaps in contraceptive use. A further evaluation of contraceptive use dynamics, using calendar data, was beyond the scope of this analysis but could provide a more thorough investigation of contraceptive gaps, albeit limited by the quality of the calendar data [22].

Despite these limitations, we believe the current study provides important insights on the resilience of women across geographically and socially diverse contexts in sub-Saharan Africa in sustaining

For peer review only - http://bmjopen.bmj.com/site/about/guidelines.xhtml

contraceptive practices amid the COVID-19 pandemic. Renewed national and international commitments to family planning made as part of the 2011 Ouagadougou Partnership [21] and 2012 London Summit on Family Planning [23] have likely contributed to the resiliency and innovations in family planning delivery systems; however, investments in these systems must be sustained to ultimately reduce unsatisfied demand for contraception and achieve universal access to SRH services. With no end to the pandemic in sight, rising global poverty, and the potential diversion of international family planning funding, there is continued cause for concern over women's access to affordable contraceptive services in-Sub-Saharan Africa—sustained advocacy and monitoring can ensure accountability and progress towards essential SRH service provision, including women's access to and use of contraception.

For peer review only

For peer review only - http://bmjopen.bmj.com/site/about/guidelines.xhtml

REFERENCES

1 Riley T, Sully E, Ahmed Z, *et al.* Estimates of the Potential Impact of the COVID-19 Pandemic on Sexual and Reproductive Health In Low- and Middle-Income Countries. *Int Perspect Sex Reprod Health* 2020;**46**:73–6.

2 UNFPA. Impact of the COVID-19 Pandemic on Family Planning and Ending Gender-based Violence, Female Genital Mutilation and Child Marriage. 2020. https://www.unfpa.org/sites/default/files/resource-pdf/COVID-19_impact_brief_for_UNFPA_24_April_2020_1.pdf

3 Hall KS, Samari G, Garbers S, *et al.* Centring sexual and reproductive health and justice in the global COVID-19 response. *Lancet* 2020;**395**:1175–7. doi:10.1016/S0140-6736(20)30801-1

4 Darroch JE, Singh S. Trends in contraceptive need and use in developing countries in 2003, 2008, and 2012: An analysis of national surveys. *Lancet* 2013;**381**:1756–62. doi:10.1016/S0140-6736(13)60597-8

5 Wood SN, Karp C, OlaOlorun F, *et al.* Trends in the need for and use of contraception before and during COVID-19 in four sub-Saharan African geographies: results from population-based national or regional cohort surveys. *Lancet Glob Heal* 2021;**9**:e793-801.

6 Karp C, Wood SN, Guiella G, *et al.* Contraceptive dynamics during COVID-19 in sub--Saharan Africa: longitudinal evidence from Burkina Faso and Kenya. *BMJ Sex Reprod Heal* 2021;**0**:1–9. doi:10.1136/bmjsrh-2020-200944

7 Gummerson E, Cardona C, Anglewicz P, *et al.* The wealth gradient and the effect of COVID-19 restrictions on income loss, food insecurity and health care access in four sub-Saharan African geographies. *PLoS One* 2021;**16**:e0260823. doi:10.1371/journal.pone.0260823

8 Mahler DG, Yonzan N, Lakner C, *et al.* Updated estimates of the impact of COVID-19 on global poverty: Turning the corner on the pandemic in 2021? World Bank Data Blog. 2021;:1–10.https://blogs.worldbank.org/opendata/updated-estimates-impact-covid-19-global-poverty-turning-corner-pandemic-2021

9 Our World in Data. Policy Responses to the Coronavirus Pandemic--Statistics and Research. https://ourworldindata.org/policy-responses-covid (accessed 26 Jan 2022).

10 Pinchoff J, Austrian K, Rajshekhar N, *et al.* Gendered economic, social and health effects of the COVID-19 pandemic and mitigation policies in Kenya: Evidence from a prospective cohort survey in Nairobi informal settlements. *BMJ Open* 2021;**11**:1–11. doi:10.1136/bmjopen-2020-042749

11 Decker MR, Wood SN, Thiongo M, *et al.* Gendered health, economic, social and safety impact of COVID-19 on adolescents and young adults in Nairobi, Kenya. *PLoS One* 2021;**16**:e0259583.

12 Karp C, Moreau C, Sheehy G, *et al.* Youth Relationships in the Era of COVID-19: A Mixed-Methods Study Among Adolescent Girls and Young Women in Kenya. *J Adolesc Heal* 2021;**69**:754–61. doi:10.1016/j.jadohealth.2021.07.017

13 Greenleaf AR, Gadiaga A, Guiella G, *et al.* Comparability of modern contraceptive use estimates between a face-to-face survey and a cellphone survey among women in Burkina Faso. *PLoS One* 2020;**15**:1–15. doi:10.1371/journal.pone.0231819

14 Thomas AB, Yaya S. Looking ahead in the COVID‑19 pandemic : emerging lessons learned for sexual and reproductive health services in low‑ and middle‑ income countries. *Reprod Health* 2021;**4**:1–8.

For peer review only

15 Leight J, Hensly C, Chissano M, *et al.* Short-term effects of the COVID-19 state of emergency on contraceptive access and utilization in Mozambique. *PLoS One* 2021;**16**:1–16. doi:10.1371/journal.pone.0249195

16 Ameh C, Banke-Thomas A, Balogun M, *et al.* Reproductive maternal and newborn health Providers' assessment of facility preparedness and its determinants during the COVID-19 pandemic in Lagos, Nigeria. *Am J Trop Med Hyg* 2021;**104**:1495–506. doi:10.4269/ajtmh.20-1324

17 Karp C, Moreau C, Olaolorun F, *et al.* Family planning service disruptions amid COVID-19: Longitudinal evidence from facilities in seven low- and middle-income countries Authors. 2021. https://ipc2021.popconf.org/uploads/211072

18 Mickler AK, Carrasco MA, Raney L, *et al.* Applications of the High Impact Practices in Family Planning during COVID-19. *Sex Reprod Heal Matters* 2021;**29**. doi:10.1080/26410397.2021.1881210

19 PSI. Serving Sara during the COVID-19 pandemic: Lessons learned in family planning adapatations. 2021;:1–13.https://www.psi.org/wp-content/uploads/2021/08/PSI_COVID_Briefs_Final_29July2021.pdf

20 Government of Kenya M of H. Kenya COVID19 RMNH Guidelines: A Kenya Practical Guide for Continuity of Reproductive, Maternal, Newborn and Family Planning Care and Services in the Background of COVID19 Pandemic. 2020. https://kenya.unfpa.org/sites/default/files/pub-pdf/KENYA - COVID19 RMNH GUIDELINES.pdf

21 The Ouagadougou Partnership. 2021.https://partenariatouaga.org/en/about-us/the-partnership/ (accessed 27 Jan 2022).

22 Bradley SE., Sarnak D, Breckler E, *et al.* Measuring Dynamic Contraceptive Use: The Quality and Consistency of DHS Calendar Data. 2021.

23 The London Summit on Family Planning. 2012.

Funding: This research was developed under grants OPP1198333 and OPP1198339 awarded by the Bill & Melinda Gates Foundation to Johns Hopkins Bloomberg School of Public Health and Jhpiego.

Reflexivity statement. The PMA project brings together a consortium of researchers from the global South who lead the project and their respective geographies and researchers from the University of Hopkins who oversee cross-site PMA activities. The PMA research objectives, study design and instruments are co-developed based on common LMIC priorities and, each partner includes site-specific indicators to address their local priorities. The research is conducted collaboratively, each partner indicating their interests in leading or collaborating on manuscripts. PMA data are made publicly available, and results are published in open access journals to ensure publication dissemination in partner countries.

Contributions: FOO, PA, GG, PG, LZ and PA designed the PMA panel study, CM, FOO, PA, GG, PG, LZ and PA design the COVID module, CM, CK, SW designed this particular study. CM conducted the analysis, CM and KW conducted the literature review, CM, KW, CK, SW wrote the initial draft, and FOO, PA, GG, PG, LZ, PA interpreted the results and revised the manuscript.

Competing Interests: None declared.

Data Availability Statement: Data are publicly available at pmadata.org.

For peer review only - http://bmjopen.bmj.com/site/about/guidelines.xhtml

Figure 1: Changes in individual level risk of unintended pregnancy, and use of contraception between Phases 1 and 2, and contraceptive method mix between adopters and discontinuers of contraception

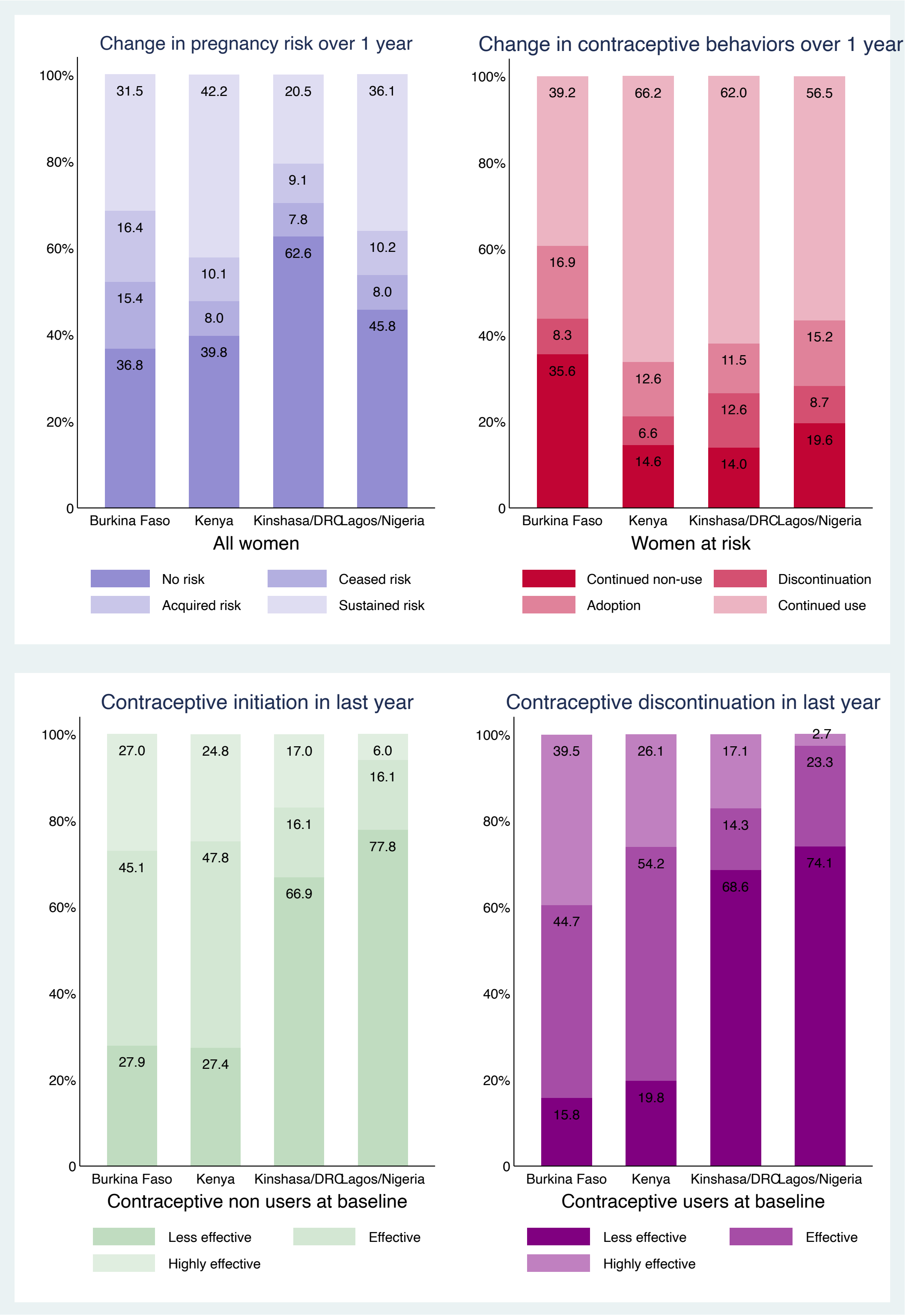

APPENDIX 1. Sociodemographic factors related to contraceptive adoption and discontinuation between Phases 1 and 2--results of multivariable logistic regression models, per setting

	Burkina Faso		Kenya		Kinshasa-DRC		Lagos-Nigeria	
	Adoption (n=972)	Discontinuation. (n=917)	Adoption (n=929)	Discontinuation. (n=2183)	Adoption (n=126)	Discontinuation. (n=321)	Adoption (n=156)	Discontinuation. (n=271)
	aOR (95% CI)							
Age								
15-24	ref	ref	ref	ref	ref	ref	ref	ref
25-34	1.3 (0.7-2.5)	**3.7 (1.1,12.0)**	0.7 (0.4-1.3)	1.2 (0.6-2.32)	0.5 (0.1-2.7)	2.1 (0.5-9.0)	-	1.0 (0.2-6.6)
35-49	1.0 (0.4-2.3)	2.9 (0.8-11.1	**0.4 (0.2-0.8)**	**2.0 (1.1-3.9)**	**0.1 (0.0-0.8)**	0.9 (0.2-4.7)	3.9 (0.3-51.3)	3.0 (0.4-24.7
Parity								
0-1	ref	ref	ref	ref	ref	ref	ref	ref
2-3	2.0 (1.0-3.9)	0.4 (0.1-1.6)	1.9 (1.0-3.8)	**0.4 (0.3-0.8)**	2.1 (0.10.4)	0.4 (0.2-1.6)	0.3 (0.0-2.3)	0.5 (0.1-1.9)
4+	1.6 (0.6-4.8)	0.9 (0.3-3.3)	1.4 (0.7-2.9)	**0.5 (0.2-0.9)**	1.3 (0.2-7.1)	0.6 (0.2-1.6)	0.6 (0.4-6.1)	0.3 (0.1-1.)
Residence								
Urban	1.3 (0.7-2.3)	0.8 (0.3-2.-2)	1.1 (0.7-1.6)	0.8 (0.5-1.3)	--	--	--	--
Rural	ref	ref	ref	ref	--	--	--	
Education								--
No schooling /primary	ref	ref	ref	ref	ref	ref	ref	ref
Secondary low	**6.0 (2.8-12.8)**	1.6 (0.6-4.5)	1.1 (0.5-2.8)	0.6 (0.1-2.2)	1.3 (0.2-7.1)	**0.4 (0.2-1.0)**	1.6 (0.4-6.1)	0.6 (0.2-2.0)
Secondary high	**22.6 (4.4-116.7)**	0.9 (0.3-3.3)	**2.0 (1.3-3.0)**	1.2 (0.8-1.7)	0.8 (0.1-7.5)	0.6 (0.2-2.7)	3.5 (0.8-15.4)	0.4 (0.1-2.0)
Food insecurity								
Yes	0.8 (0.3-2.3)	1.1 (0.3,3.6)	0.9 (0.6-1.5)	1.2 (0.6-2.1)	0.3 (0.1-1.3)	0.7 (0.3-1.5)	**-**	0.4 (0.0-3.8)
No	ref	ref	ref	ref	ref	ref	ref	ref
Wealth								
Poorest	ref	ref	ref	ref	ref	ref	ref	ref
Medium	0.7 (0.4-1.2)	0.7 (0.4,1.3)	1.4 (0.9-2.0)	1.0 (0.6-1.5)	0.8 (0.2-2.9)	0.9 (0.4-2.1)	0.7 (0.2-2.46	0.8 (0.3-2.2)
Richest	0.8 (0.4-1.5)	0.5 (0.2-1.6)	**2.0 (1.2-3.4)**	1.0 (0.6-1.7)	0.9 (0.2-3.1)	0.6 (0.2-1.4)	1.2 (0.4-3.9)	0.6 (0.2-2.1)

For peer review only

APPENDIX 2. Sociodemographic factors related to having an unintended pregnancy in Phase 2--results of multivariable logistic regression models, per setting

	Burkina Faso N=4018	Kenya N=5616	Kinshasa-DRC N=1489	Lagos-Nigeria N=836
Age				
15-24	ref	ref	ref	ref
25-34	**0.2 (0.1-0.5)**	0.7 (0.5-1.2)	0.4 (0.2-1.1)	0.3 (0.1,1.0)
35-49	**0.1 (0.0-0.4)**	**0.1 (0.1-0.3)**	**0.1 (0.0-0.3)**	**0.1 (0.0-0.3)**
Marital status				
Not in a cohabitating union	ref	ref	ref	ref
Married or in union	1.1 (0.4-3.1)	0.7 (0.45-1.2)	**2.4 (1.1-5.1)**	2.9 (0.4-21.6)
Parity				
0-1	ref	ref	ref	ref
2-3	1.6 (0.7-3.7)	1.2 (0.7-2.2)	**3.6 (1.4-8.9)**	1.3 (0.1-10.9)
4+	**5.1 (1.3-19.9)**	1.4 (0.6-3.0)	**3.7 (1.2-11.4)**	0.8 (0.0-21.2)
Residence				
Urban	0.9 (0.4-1.9)	0.9 (0.4-1.6)	--	--
Rural	ref	ref	--	--
Education				
No schooling /primary	ref	ref	ref	ref
Secondary low	1.6 (0.8-3.2)	0.4 (0.1-1.6)	0.5 (0.2-1.3)	0.6 (0.1-4.3)
Secondary high	0.6 (0.2-2.0)	**0.4 (0.2-0.7)**	0.6 (0.1-3.2)	0.3 (0.0-3.5)
Food insecurity				
Yes	1.6 (0.7-3.8)	1.3 (0.7-2.2)	0.9 (0.6-2.0)	-
No	ref	ref	ref	
Wealth				
Poorest	ref	ref	ref	ref
Medium	1.4 (0.8-2.7)	**0.6 (0.3-0.9)**	**0.4 (0.2-1.0)**	0.3 (0.0-2.1)
Richest	0.8 (0.3-2.0)	**0.4 (0.2-0.8)**	0.5 (0.2-1.2)	1.1 (0.2-5.1)
Contraception at phase 1				
None	ref	ref	ref	ref
Less effective (barrier/natural)	1.0 (0.3-2.8)	1.4 (0.8-2.7)	1.1 (0.6,5.1)	0.3 (0.0,1.4)
Effective (short-acting hormonal)	1.4 (0.7-2.9)	1.1 (0.6-1.9)	**1.8 (0.2-0.8)**	0.4 (0.0-5.3)
Highly effective (long-acting methods)	**0.1 (0.0-0.2)**	**0.4 (0.2-0.8)**	**0.1 (0.0-0.3)**	**0.1 (0.0-0.8)**

± Less effective=barrier or natural methods; effective= short-acting hormonal methods, including pills, injectables, patches, or rings; highly effective=IUDs, implants, sterilization

For peer review only

APPENDIX 3. Sociodemographic characteristics by sample and setting, weighted

	Burkina Faso			Kenya			Kinshasa-DRC			Lagos-Nigeria		
Overall	Phase 1[a]	Phase 2	Panel	Phase 1	Phase 2	Panel	Phase 1	Phase 2	Panel	Phase 1	Phase 2	Panel
Age												
15-19	21.6	20.9	20.9	21.6	22.3	21.9	22.2	23.2	22.3	14.0	15.8	13.4
20-24	17.3	18.4	17.4	17.6	17.4	17.7	20.9	20.4	21.4	14.0	12.6	12.8
25-29	14.7	15.0	14.8	16.3	15.5	16.3	16.3	15.2	16.0	15.5	16.0	15.7
30-34	15.6	15.2	16.1	15.4	15.6	15.1	12.3	13.4	12.3	17.7	16.1	18.1
35-39	12.7	12.9	12.6	11.8	11.6	11.8	10.7	10.8	10.7	17.8	16.8	18.4
40-44	10.9	10.9	11.1	9.5	9.6	9.4	10.4	10.2	10.2	12.7	12.8	13.0
45-49	7.2	6.9	7.1	7.8	8.1	7.9	7.2	6.8	7.2	8.3	10.0	8.5
Parity												
Nulliparous	24.3	22.7	23.2	28.3	29.2	28.5	41.9	41.4	41.7	35.2	36.5	32.9
1-2	24.8	24.9	24.9	30.9	30.1	29.9	28.1	28.7	28.7	29.5	28.8	30.4
3+	50.9	52.5	51.9	40.9	40.7	41.6	30.1	30.0	29.7	35.3	34.7	36.7
Residence												
Urban	22.8	22.3	22.9	30.2	30.4	30.5	100.0	100.0	100.0	100.0	100.0	100.0
Rural	77.2	77.7	77.1	69.8	69.6	69.5	--	0.0	0.0	0.0	0.0	0.0
Wealth Tertile												
Poorest	330	33.5	33.1	35.9	34.6	35.8	33.9	31.7	33.6	34.2	33.0	34.7
Middle	33.5	32.9	33.2	33.5	34.2	33.6	32.3	33.0	32.7	30.7	33.0	30.8
Richest	33.5	33.7	33.7	30.6	31.3	30.6	33.8	35.4	33.8	35.1	34.0	34.6
Loss of income last 12 months												
None		0.0	0.0		22.4	21.1		0.0	0.0		0.0	0.0
Complete		9.2	9.7		25.2	25.0		41.4	41.7		29.0	32.5
Partial		90.8	90.3		52.4	53.9		58.6	58.3		71.0	67.6
Food insecurity												
No		93.8	93.9		88.1	88.9		79.5	81.0		92.0	92.4
Yes		6.2	6.1		11.9	11.1		20.5	19.0		8.0	7.6
Education												
None/primary	77.6	75.7	77.7	49.7	47.5	49.2	7.8	6.7	7.8	11.8	11.5	12.1
Secondary low	16.4	17.3	16.1	2.1	1.6	2.0	72.9	75.1	72.9	51.2	52.8	51.3
Secondary high+	6.1	7.0	6.2	48.2	50.8	48.8	19.3	18.2	19.3	37.0	35.7	36.6
Contraception												
None	67.7	70.1	68.5	50.4	52.2	52.2	57.1	55.4	55.1	60.2	60.6	60.2
Less effective	7.8	5.7	5.8	9.7	7.2	7.2	31.3	32.7	32.3	27.1	25.8	25.6
Effective	10.3	9.9	10.4	20.5	19.2	19.6	4.3	4.2	10.4	6.2	6.1	6.7
Highly effective	14.0	14.3	15.3	19.4	21.4	21.2	7.3	7.8	15.3	6.5	7.5	7.5

± Less effective=barrier or natural methods; effective= short-acting hormonal methods, including pills, injectables, patches, or rings; highly effective=IUDs, implants, sterilization

For peer review only

STROBE Statement—Checklist of items that should be included in reports of ***cross-sectional studies***

	Item No	Recommendation	Pg. & Line
**Title and abstract**	1	(*a*) Indicate the study's design with a commonly used term in the title or the abstract	1
		(*b*) Provide in the abstract an informative and balanced summary of what was done and what was found	3
**Introduction**			
Background/rationale	2	Explain the scientific background and rationale for the investigation being reported	4
Objectives	3	State specific objectives, including any prespecified hypotheses	4
**Methods**			
Study design	4	Present key elements of study design early in the paper	4
Setting	5	Describe the setting, locations, and relevant dates, including periods of recruitment, exposure, follow-up, and data collection	4
Participants	6	(*a*) Give the eligibility criteria, and the sources and methods of selection of participants	5
Variables	7	Clearly define all outcomes, exposures, predictors, potential confounders, and effect modifiers. Give diagnostic criteria, if applicable	6-7
Data sources/ measurement	8*	For each variable of interest, give sources of data and details of methods of assessment (measurement). Describe comparability of assessment methods if there is more than one group	*6-7*
Bias	9	Describe any efforts to address potential sources of bias	7
Study size	10	Explain how the study size was arrived at	4
Quantitative variables	11	Explain how quantitative variables were handled in the analyses. If applicable, describe which groupings were chosen and why	7
Statistical methods	12	(*a*) Describe all statistical methods, including those used to control for confounding	7
		(*b*) Describe any methods used to examine subgroups and interactions	7
		(*c*) Explain how missing data were addressed	
		(*d*) If applicable, describe analytical methods taking account of sampling strategy	7
		(*e*) Describe any sensitivity analyses	
**Results**			
Participants	13*	(a) Report numbers of individuals at each stage of study—eg numbers potentially eligible, examined for eligibility, confirmed eligible, included in the study, completing follow-up, and analysed	7-8
		(b) Give reasons for non-participation at each stage	6
		(c) Consider use of a flow diagram	
Descriptive data	14*	(a) Give characteristics of study participants (eg demographic, clinical, social) and information on exposures and potential confounders	8
		(b) Indicate number of participants with missing data for each variable of interest	9-10
Outcome data	15*	Report numbers of outcome events or summary measures	10

For peer review only

For peer review only - http://bmjopen.bmj.com/site/about/guidelines.xhtml

Main results	16	(*a*) Give unadjusted estimates and, if applicable, confounder-adjusted estimates and their precision (eg, 95% confidence interval). Make clear which confounders were adjusted for and why they were included	11-12
		(*b*) Report category boundaries when continuous variables were categorized	
		(*c*) If relevant, consider translating estimates of relative risk into absolute risk for a meaningful time period	
Other analyses	17	Report other analyses done—eg analyses of subgroups and interactions, and sensitivity analyses	
**Discussion**			
Key results	18	Summarise key results with reference to study objectives	12
Limitations	19	Discuss limitations of the study, taking into account sources of potential bias or imprecision. Discuss both direction and magnitude of any potential bias	13
Interpretation	20	Give a cautious overall interpretation of results considering objectives, limitations, multiplicity of analyses, results from similar studies, and other relevant evidence	12-13
Generalisability	21	Discuss the generalisability (external validity) of the study results	13
**Other information**			
Funding	22	Give the source of funding and the role of the funders for the present study and, if applicable, for the original study on which the present article is based	1

*Give information separately for exposed and unexposed groups.

Note: An Explanation and Elaboration article discusses each checklist item and gives methodological background and published examples of transparent reporting. The STROBE checklist is best used in conjunction with this article (freely available on the Web sites of PLoS Medicine at http://www.plosmedicine.org/, Annals of Internal Medicine at http://www.annals.org/, and Epidemiology at http://www.epidem.com/). Information on the STROBE Initiative is available at www.strobe-statement.org.

For peer review only

Trends in fertility intentions and contraceptive practices in the context of COVID-19 in sub-Saharan Africa: Insights from four national and regional population-based cohorts

Journal:	*BMJ Open*
Manuscript ID	bmjopen-2022-062385.R1
Article Type:	Original research
Date Submitted by the Author:	15-Aug-2022
Complete List of Authors:	Moreau, Caroline; Johns Hopkins University Bloomberg School of Public Health, Department of Population Family and Reproductive Health; INSERM, Soins et Santé Primaire, CESP Centre for Research in Epidemiology and Population Health U1018<br>Karp, Celia; Johns Hopkins University Bloomberg School of Public Health<br>Wood, Shannon; Johns Hopkins University Bloomberg School of Public Health<br>Williams, Kelsey; Johns Hopkins University Bloomberg School of Public Health, Department of Population Family and Reproductive Health<br>Olaolorun, Funmilola; University of Ibadan College of Medicine<br>Akilimali, Pierre; DRC<br>Guiella, Georges; Universite de Ouagadougou<br>Gichangi, Peter; International Centre for Reproductive Health Kenya<br>Zimmerman, Linnea; Johns Hopkins Bloomberg School of Public Health, Population Family and Reproductive Health<br>Anglewicz, Philip; Johns Hopkins University, Population, Family, and Reproductive Health
**Primary Subject Heading**:	Public health
Secondary Subject Heading:	Sexual health
Keywords:	COVID-19, GYNAECOLOGY, REPRODUCTIVE MEDICINE

I, the Submitting Author has the right to grant and does grant on behalf of all authors of the Work (as defined in the below author licence), an exclusive licence and/or a non-exclusive licence for contributions from authors who are: i) UK Crown employees; ii) where BMJ has agreed a CC-BY licence shall apply, and/or iii) in accordance with the terms applicable for US Federal Government officers or employees acting as part of their official duties; on a worldwide, perpetual, irrevocable, royalty-free basis to BMJ Publishing Group Ltd ("BMJ") its licensees and where the relevant Journal is co-owned by BMJ to the co-owners of the Journal, to publish the Work in this journal and any other BMJ products and to exploit all rights, as set out in our licence.

The Submitting Author accepts and understands that any supply made under these terms is made by BMJ to the Submitting Author unless you are acting as an employee on behalf of your employer or a postgraduate student of an affiliated institution which is paying any applicable article publishing charge ("APC") for Open Access articles. Where the Submitting Author wishes to make the Work available on an Open Access basis (and intends to pay the relevant APC), the terms of reuse of such Open Access shall be governed by a Creative Commons licence – details of these licences and which Creative Commons licence will apply to this Work are set out in our licence referred to above.

Other than as permitted in any relevant BMJ Author's Self Archiving Policies, I confirm this Work has not been accepted for publication elsewhere, is not being considered for publication elsewhere and does not duplicate material already published. I confirm all authors consent to publication of this Work and authorise the granting of this licence.

For peer review only

Trends in fertility intentions and contraceptive practices in the context of COVID-19 in sub-Saharan Africa: Insights from four national and regional population-based cohorts

Caroline Moreau[1,2]
Celia Karp[1]
Shannon N. Wood[1]
Kelsey Williams[1]
Funmilola M. OlaOlorun[3]
Pierre Akilimali[4]
Georges Guiella[5]
Peter Gichangi[6,7]
Linnea A. Zimmerman[1]
Philip A. Anglewicz[1]

[1] Department of Population Family and Reproductive Health, Johns Hopkins Bloomberg School of Public Health, Baltimore, Maryland, USA
[2] Soins et Santé Primaire, CESP Centre for Research in Epidemiology and Population Health U1018, Inserm, F-94805 Villejuif, France
3 College of Medicine, University of Ibadan, Ibadan, Nigeria
4 Kinshasa School of Public Health, Kinshasa, DRC.
5 Institut Supérieur des Sciences de la Population (ISSP/University of Ouagadougou), Ouagadougou, Burkina Faso
6 International Centre for Reproductive Health-Kenya, Nairobi, Kenya, Technical University of Mombasa;
7 Department of Public Health and Primary Care, Faculty of Medicine and Health Sciences, Ghent University, Belgium;

Corresponding author
Caroline Moreau, MD, PhD
Department of Population, Family and Reproductive Health
Johns Hopkins Bloomberg School of Public Health
615 N. Wolfe Street Rm. E4537 Baltimore, MD USA 21205
cmoreau2@jhu.edu

Word count: 3,584/5,000
Figures: 1
References: 23
Tables: 4 + 3 in Appendix

For peer review only

ABSTRACT

Objectives: Studies in several sub-Saharan geographies conducted early in the COVID-19 pandemic suggested little impact on contraceptive behaviors. Initial results may mask widening disparities with rising poverty, and changes to women's pregnancy desires and contraceptive use amid prolonged health service disruptions. This study examined trends in contraceptive behaviors in four sub-Saharan African settings one year into the pandemic.

Design: Nationally and regionally representative longitudinal surveys

Setting: Burkina Faso, Kenya, Democratic Republic of Congo (Kinshasa), and Nigeria (Lagos)

Participants: Women 15-49 years with sample size ranging from 1,469 in Nigeria to 9,477 in Kenya.

Outcome measures: Fertility preferences, contraceptive use, and unintended pregnancies measured before COVID-19 (November 2019-January 2020) and during COVID-19 (November 2020-January 2021).

Analysis: We described population-level and individual-level changes by socioeconomic characteristics using generalized equation modeling. We used logistic regression models to identify factors related to contraceptive adoption and discontinuation and to experiencing an unintended pregnancy.

Results: At the population level, we found no change in women's exposure to unintended pregnancy risk, alongside 5 to 9 percentage point increases in contraceptive prevalence in Burkina Faso, Kenya, and Lagos. Reliance on provider-dependent methods dropped by 2 and 4 percentage points in Kenya and Burkina Faso, respectively, although these declines were not statistically significant. Between 1.0% and 2.8% of women across sites experienced an unintended pregnancy during COVID-19, with no significant change over time. Individual-level trajectories showed contraceptive adoption was more common than discontinuation in Burkina Faso, Kenya, and Lagos, with little difference by sociodemographic characteristics. Women's COVID-19-related economic vulnerability was unrelated to unintended pregnancy across sites.

Conclusions:
This study highlights the resilience of African women across diverse settings in sustaining contraceptive practices amid the COVID-19 pandemic. However, with reports of rising poverty in sub-Saharan Africa, there is continued need to monitor access to essential sexual and reproductive health services.

For peer review only

STRENGTHS AND LIMITATIONS

- The study uses a longitudinal design to assess individual change in contraceptive behaviors since the onset of the COVID-19 pandemic.
- Nationally or regionally representative survey data were used to evaluate population level change in contraceptive behaviors since the onset of the COVID-19 pandemic.
- This investigation spans four diverse geographies in sub-Saharan Africa, allowing for a comparison of COVID-19 impact by social and pandemic contexts.
- Rates of loss to follow-up ranged from 21-27%, likely affecting study estimates, although the use of post-stratification weights reduces attrition biases.
- Data from two time points prevents the estimation of gaps in contraceptive use over the entire COVID-19 pandemic.

For peer review only

INTRODUCTION

The COVID-19 pandemic, which started in late 2019, quickly traversed the globe, creating health and economic crisis. By the end of 2020, the SARS-CoV-2 virus had resulted in nearly two million deaths [1], destroyed 114 million jobs [2], and debilitated global economic growth rates, which fell to an annualized negative rate of -3.2 [2]. With 65,468 fatalities, the sub-Saharan continent bore less of a health burden in this early stage of the pandemic [1], but swift policy containment measures led to unprecedented economic loss, with a 1.9% contraction in the Gross Domestic Product (GDP) per capita and an estimated 32 million additional people living in extreme poverty [3].

Loss of household income [4] coupled with restricted access to essential health services , including family planning [5-6] were expected to significantly alter fertility intentions and behaviors, [7–9], especially in sub-Saharan Africa [10], given pre-existing high unmet need for sexual and reproductive health (SRH) services [11]. In 2019, the unintended pregnancy rate (91 per 1000 women per year) in sub-Saharan Africa was the highest in the world [12] and only 55% of women's need for contraception was satisfied [13]. However, initial empirical evidence from four sub-Saharan African countries, Burkina Faso, Democratic Republic of Congo (DRC), Kenya, and Nigeria, using Performance monitoring for Action (PMA) data, suggested little impact on women's use of contraception in the early stages of the pandemic [14]. In fact, the study found a rise in contraceptive prevalence in rural Burkina Faso and across Kenya. These data were corroborated with findings further exploring changes in women's individual-level contraceptive behaviors in the early months of the pandemic in Burkina Faso and Kenya, indicating that more women adopted contraception than discontinued their methods between the end of 2019 and mid-2020 (13% Burkina Faso and 25% Kenya versus 5% Burkina Faso and 6% Kenya, respectively) [15].

While these initial studies were pivotal in reporting early pandemic trends, the longer term societal impacts of COVID-19, including rising poverty [3,16] and interruptions to schooling and employment [17], were likely to affect relationships and childbearing decisions [18-19]. At the same time, growing economic disparities were projected to affect people's financial access and use of health services [4,20], potentially reducing their ability to achieve their reproductive goals. Furthermore, early impact estimates were drawn from phone-based surveys amid COVID-19 lockdowns, which may have affected the interpretation of early study findings due to selection bias and data collection mode [14,15,21]. Recent analysis estimating changes in modern contraception and pregnancy rates using repeated cross-sectional surveys collected face to face shed light on these trends. Specifically, in Burkina Faso, Kenya, Kinshasa (DRC), and Lagos (Nigeria), data collected in pre-pandemic periods and one year into the pandemic, suggest slight decreases in pregnancy rates among younger, less educated women in Burkina Faso and a rise in modern contraception among young women in all geographies, except Lagos [22].

Expanding on these results, the present study describes population- and individual-level changes in women's fertility preferences, contraceptive behaviors, and unintended pregnancy experiences in the first year of the COVID-19 pandemic using cross-sectional representative data and panel data collected in the same sub-Saharan African geographies. Specifically, we examined how these trends differed by sociodemographic characteristics and COVID-19-related economic hardship.

For peer review only - http://bmjopen.bmj.com/site/about/guidelines.xhtml

Finally, we assessed factors related to individual-level changes in contraceptive behaviors and to experiencing an unintended pregnancy during the pandemic.

METHODS

Samples

We used two rounds of publicly available data (available at www.pmadata.org) collected in November 2019-January 2020 (3-4 months before the pandemic) and one year later (November 2020-January 2021) from nationally or regionally representative, population-based surveys. Surveys were implemented by the Performance Monitoring for Action (PMA) project among women aged 15-49 in four sub-Saharan African geographies. Surveys are nationally representative in Burkina Faso and Kenya, and regionally representative in Kinshasa province in DRC and Lagos State in Nigeria. We did not include data from Kongo Central in the DRC and Kano State in Nigeria, also collected through the PMA platform due to smaller sample size and lack of statistical power to show any reduction in contraceptive use in these two geographies. Women were identified using a multistage clustered sampling design, starting with the random selection of census enumeration areas (EAs) after stratifying by rural and urban area (in Kenya and Burkina Faso), followed by the random selection of 35 households per EA. All women of reproductive age (15-49 years) from the selected households were eligible for inclusion. Sample sizes were determined to produce modern contraceptive prevalence rates (mCPRs) with a precision of +/- 3% at the national or regional level, leading to sample size variation by geography (Table 1).

Patient and Public involvement

This study is a population-based study that does not include patients. The public was not involved in this research.

Data collection

Pre-pandemic data (PMA's Phase 1 surveys) were collected face-to-face by trained resident interviewers before COVID-19 (November 2019-January 2020). Women who consented to be followed-up as part of PMA's panel design were re-interviewed in-person, one year later (PMA's Phase 2 surveys), during the pandemic (November 2020-January 2021). Replacement households from the EA were also included in Phase 2 to account for attrition at the household level (projected at 15%). The survey design allows for longitudinal analysis to examine individual-level changes since the onset of COVID-19 and for annual cross-sectional analysis to track national or regional level indicators (including household replacements). Follow-up rates between Phase 1 and Phase 2 surveys ranged from 73% in Kenya to 79% in Burkina Faso (Table 1). No differences between Phase 1 and Phase 2 cross-sectional sample characteristics were observed; differential lost to follow-up in the panel sample was accounted for by applying post-stratification weights (more details available at www.pmadata.org).

For peer review only - http://bmjopen.bmj.com/site/about/guidelines.xhtml

Table 1. Number of women aged 15 to 49 years included in each PMA survey and follow-up rate, by setting

Country/Site	Phase 1-Baseline	Phase 2 cross-sectional-including household replacement	Phase 2 panel-no replacement sample
Burkina Faso	6,590	6,362	5,207 (79%)
Kenya	9,477	9,310	6,932 (73%)
Kinshasa, DRC	2,611	2,368	1,967 (75%)
Lagos, Nigeria	1,469	1,481	1,088 (74%)

DRC=Democratic Republic of Congo.
Follow-up rate reported as % of all baseline women who successfully completed the Phase 2 survey

Ethical Approval

PMA surveys received approval from ethical committees in each geography (Burkina Faso: Comité d'Ethique Institutionnel Pour La Recherche en Santé, Ministère de l'Enseignement Supérieur, de la Recherche Scientifique et de l'Innovation; Democratic Republic of the Congo: University of Kinshasa School of Public Health; Kenya: Kenyatta National Hospital—University of Nairobi Ethics Research Committee; Nigeria: Lagos State University Teaching Hospital Health Research Ethics Committee). PMA also received approval from the Institutional review board of the Johns Hopkins School of Public Health (IRB00014702). Public health preventive measures (i.e., social distancing, mask-wearing, use of hand sanitizer) were instituted in Phase 2 to prevent COVID-19 transmission during data collection.

Measures

At each survey, women were asked about their current pregnancy status, whether the pregnancy was wanted or happened at the right time, future fertility intentions, and current contraceptive behaviors. This information was operationalized as indicators used for population-level comparisons (cross-sectional measures) and for individual-level trends analysis (longitudinal measures).

Cross-sectional measures

We explored six measures reflecting women's sexual and reproductive health experiences before and during the pandemic. *Women's fertility intentions for the next year* distinguished women who intended to have a child in the next year from those who wanted no more children or wanted to delay childbearing for more than one year. *Exposure to the risk of unintended pregnancy* (yes/no) classified women who had sexual intercourse in the last 12 months, were fecund, and were not pregnant or trying to have a child in the next year as being "at risk". Among women at risk of unintended pregnancy, *use of contraception* (yes/no) classified women who were using barrier and natural methods, pills, injectables, patches, rings, implants, IUDs, and sterilization as using contraception. Among contraceptive users, *use of a provider-dependent method* (yes/no) classified women using methods generally requiring interaction with a healthcare provider (i.e., pills, injectables, implants, IUDs, sterilization) as provider-dependent method users. We also considered a three-category measure of contraceptive method types, grouping contraceptive users according to the effectiveness of their contraceptive method—users of *less effective* methods (i.e., barrier or

For peer review only - http://bmjopen.bmj.com/site/about/guidelines.xhtml

natural methods), users of *effective* methods (i.e., short-acting hormonal methods, including pills, injectables, patches, or rings), and users of *highly effective* methods (i.e., implants, IUDs, or sterilization). *Pregnancy status* identified women who were pregnant at the time of each survey (yes/no), while an indicator of *unintended* (yes/no) identified women who were pregnant and indicated they had not wanted to become pregnant or wanted to become pregnant later.

Longitudinal measures

We also explored individual-level changes in women's *exposure to risk of unintended pregnancy*, *contraceptive use*, and *experience of an unintended pregnancy* (reported as wanted later or not at all). Changes in women's exposure to unintended pregnancy risk across the two surveys were categorized into four categories: 1) *No risk*—never at risk before or during COVID-19; 2) *Acquired risk*—newly at risk during COVID-19 (no risk pre-pandemic); 3) *Ceased risk*—no longer at risk during COVID-19 (at risk pre-pandemic); 4) *Sustained risk*—at risk before and during COVID-19. Likewise, we defined a four-category variable describing trends in any contraceptive use: 1) *Continued non-use*—non-use before or during COVID-19; 2) *Adoption*— during COVID-19 (non-use pre-pandemic); 3) *Discontinuation*—non-use during COVID-19 following use pre-pandemic; 4) *Continued use*—use before and during COVID-19. Finally, we considered the three-category indicator of contraceptive effectiveness (less effective/effective/highly effective) and a binary indicator of unintended pregnancy (yes/no) during Phase 2 (i.e., at the time of the COVID-19 survey), as previously defined.

We considered several sociodemographic and COVID-19-related factors that could impact women's use of contraception. Specifically, we assessed age, parity, marital status (in union/ not in union), education, household wealth in tertiles, and residence (urban/rural). During COVID-19, we also considered two indicators of household economic vulnerability: loss of income in the last 12 months (categorical—none, partial, complete), and food insecurity (i.e., not being able to eat for 24 hours due to lack of food since the beginning of the COVID-19 pandemic).

Analysis

We first examined population-level trends in reproductive health indicators comparing Phase 1 and Phase 2 cross sectional samples across our six indicators (proportion who intend to have a child in the next year, proportion at risk of unintended pregnancy, proportion using contraception among women who were at risk of unintended pregnancy, proportion using provider-dependent methods among users, proportion pregnant, and proportion experiencing an unintended pregnancy. We conducted the same analysis stratified by rural and urban residence in Burkina Faso and Kenya, to compare trends across urban localities (urban Burkina Faso, urban Kenya, Lagos_Nigeria and Kinshasa_DRC and rural localities in Burkina Faso and Kenya). Statistically significant differences were determined based on non-overlapping confidence intervals between Phase 1 and Phase 2 estimates.

Next, we conducted individual level analysis, focusing on the sample of women completing both surveys. We evaluated changes in exposure to unintended pregnancy risk and changes in contraceptive behaviors (among women at risk at both time points). We used generalized estimation equation models (GEE) to test for differential trends in the use of any contraception between Phase 1 and Phase 2, by women's sociodemographic characteristics (measured at Phase 1) and their experience of economic instability (measured at Phase 2) (Appendix 1). Next, we

For peer review only - http://bmjopen.bmj.com/site/about/guidelines.xhtml

examined the contraceptive method mix of contraceptive adopters and the methods women discontinued between the two surveys. We conducted three multivariable logistic regression models to identify factors related to 1) contraceptive adoption among women who were not using contraception pre-pandemic, 2) contraceptive discontinuation among women who were using a method pre-pandemic, and 3) the experience of an unintended pregnancy during the pandemic.

All analyses applied survey and post-stratification weights to account for the complex survey design, clustering of women within EAs and differential loss-to-follow-up in the case of the longitudinal sample. All analyses were conducted in Stata 16.1 with statistical significance set a priori at $p<0.05$.

RESULTS

The characteristics of pre-pandemic study samples by setting are presented in Table 2. By design, all women in Kinshasa-DRC and Lagos-Nigeria were living in urban areas, while this was the case for 22.8% and 30.2% of the samples in Burkina Faso and Kenya, respectively. The mean age of women ranged from 28.3 years in Kinshasa-DRC to 31.0 years in Lagos-Nigeria. Most women across settings were in union, except in Kinshasa, where this was the case in 42.7% of respondents. Women in Burkina Faso had the highest parity (50.9% with three or more children), followed by Kenya (40.9% with three or more children), while lower parities were observed in Kinshasa and Lagos, where 41.9% and 35.2% of women, respectively, had no children. Educational attainment varied substantially across settings, as 77.6% of women in Burkina Faso had no schooling or a primary level of education, with this proportion dropping to 49.7% in Kenya and to 7.8% and 11.8% in Kinshasa-DRC and Lagos-Nigeria, respectively. Contraceptive practices also varied, with non-use ranging from 52.2% in Kenya to 70.1% in Burkina Faso. Reliance on highly effective methods was greatest in Kenya (21.4%) and Burkina Faso (14.3%), while use of less effective barrier and natural methods was more common in Kinshasa-DRC (32.7%) and Lagos-Nigeria (25.8%).

For peer review only

Table 2. Phase 1 sociodemographic characteristics, by setting

		Burkina Faso (n=6,590)	Kenya (n=9,477)	Kinshasa-DRC (n=2,611)	Lagos-Nigeria (n=1,469)
Age (mean)		28.9	28.8	28.3	31.0
		Column %			
Age	15-19	21.6	21.6	22.2	14.0
	20-24	17.3	17.6	20.9	14.0
	25-29	14.7	16.3	16.3	15.5
	30-34	15.6	15.4	12.3	17.7
	35-39	12.7	11.8	10.7	17.8
	40-44	10.9	9.5	10.4	12.7
	45-49	7.2	7.8	7.2	8.3
Union status	In union†	75.8	59.2	42.7	60.9
	Not in union	24.2	40.8	57.3	39.1
Parity	Nulliparous	24.3	28.3	41.9	35.2
	1-2 children	24.8	30.9	28.1	29.5
	3+ children	50.9	40.9	30.1	35.3
Residence	Urban	22.8	30.2	100.0	100.0
	Rural	77.2	69.8	--	--
Education	No schooling /primary	77.6	49.7	7.8	11.8
	Secondary low	16.4	2.1	72.9	51.2
	Secondary high or more	6.1	48.2	19.3	37.0
Contraception±	None	70.1	52.2	55.4	60.6
	Less effective	5.7	7.2	32.7	25.8
	Effective	9.9	19.2	4.2	6.1
	Highly effective	14.3	21.4	7.8	7.5

301 †In union defined as married or living with a partner as if married
302 ± Less effective=barrier or natural methods; effective= short-acting hormonal methods, including pills, injectables, patches, or
303 rings; highly effective=IUDs, implants, sterilization

306 At the population level, 6.2% to 16.8% of women intended a birth in the next 12 months across geographies, with no significant change during the pandemic (Table 3), including in urban and rural areas (Appendix 2). Accounting for past-year sexual activity, fertility intentions, and fecundity, 27.8% to 46.3% of women were exposed to the risk of unintended pregnancy, with the highest proportion observed in Burkina Faso. Exposure to unintended pregnancy risk did not vary over time in any setting.

Contraceptive use (any method) varied widely by setting and was lowest in Burkina Faso and highest in Kenya and Kinshasa province in the DRC. During the pandemic, contraceptive use rose significantly by nine percentage points in Burkina Faso and five percentage points in Kenya. The rise in contraceptive use among women in Burkina Faso was twice as elevated in rural settings (10 percentage points) compared to urban settings (5 percentage points; non-significant difference)

For peer review only - http://bmjopen.bmj.com/site/about/guidelines.xhtml

while increases were comparable and statistically significant in rural and urban Kenya (Appendix 2). Contraceptive use also rose by six percentage points in Lagos-Nigeria (non-significant difference), while it remained stable in Kinshasa-DRC. The proportion of contraceptive users relying on provider-dependent methods ranged from 35.2% in Lagos to 88.3% in Kenya. This proportion remained stable in Kinshasa-DRC and Lagos-Nigeria and declines were non-significant in Burkina Faso and in Kenya overall (overlapping confidence intervals) (Table 3), but dropped by a significant 3 percentage point in rural Kenya (from 92.3 to 89.1%, Appendix 2).

Between 5.1% women in Kenya and 8.5% of women in Burkina Faso were pregnant at the time of the survey with no change over time. Finally, between 1.0% and 2.8% of women across sites had an unintended pregnant at the time of the Phase 2 survey, this proportion remaining stable over time in all sites (Table 3). No significant trends were observed among women in urban and rural settings (Appendix 2).

For peer review only

Table 3. Cross-sectional reproductive characteristics of women at Phase 1 and Phase 2, by setting

	Burkina Faso		Kenya		DRC-Kinshasa		Nigeria-Lagos	
	Phase 1 (n=6,590)	Phase 2 (n=6,362)	Phase 1 (n=9,477)	Phase 2 (n=9,31 0)	Phase 1 (n=2,611)	Phase 2 (n=2,368)	Phase 1 (n=1,469)	Phase 2 (n=1,481)
	% (95% CI)							
Intends to have a child in next year	13.4 (11.8, 15.3)	14.1 (12.3, 16.1)	6.9 (6.1, 7·8)	6.2 (5.5, 6.9)	12.2 (9.3, 15.8)	10.1 (7.8, 13.2)	16.8 (14.6, 19.2)	16.2 (14.6, 18.1)
In need of contraception	44.1 (41.1, 47.2)	44.5 (41.4, 47.4)	46.3 (44.9, 47.6)	45.6 (44.3, 46.9)	27.8 (24.7, 31.1)	29.2 (26.3, 32.2)	39.5 (36.6, 42.5)	40.4 (37.7, 43.2)
Contraceptive use, among women in need	**43.2 (39.0, 47.6)**	**52.3 (48.0, 56.6)**	**71.3 (69.4, 73.2)**	**76.5 (74.6, 78.2)**	71.8 (67.4, 75.9)	72.4 (68.2, 76.2)	63.4 (56.8, 69.5)	69.7 (63.7, 75.2)
Provider-dependent contraception, among contraceptive users	83.4 (79.3, 86.9)	79.1 (74.3, 83.1)	88.3 (86.7, 89.8)	86.3 (84.6, 87.8)	37.3 (29.5, 45.9)	35.6 (28.3, 43.5)	40.1 (33.7, 46.9)	35.2 (29.1, 41.8)
Currently pregnant	8.5 (7.6, 9.6)	7.6 (6.6, 8.4)	5.4 (4.9, 5.9)	5.1 (4.6, 5.7)	5.7 (4.5, 7.1)	5.2 (4.1, 6.5)	4.7 (3.7, 6.0)	4.7 (3.7, 5.9)
Current unintended pregnancy	2.8 (2.1,3.7)	2.1 (1.6,2.7)	2.3 (2.0,2.7)	2.1 (1.7,2.4)	2.6 (1.9,3.6)	2.8 (2.1,3.8)	1.0 (0.6-1.6)	1.0 (0.6.1.7)

Numbers in bold indicate statistical significance based the non-overlap of confidence interval

For peer review only

Assessment of individual-level trajectories of women who responded to both surveys indicated that changes in exposure to unintended pregnancy risk among all women ranged from 16.9% in Kinshasa-DRC to 31.5% in Burkina Faso (Figure 1). Patterns of change were similar in rural versus urban settings in Burkina Faso and Kenya (data not shown). Half of changes involved new exposure to risk, while the other half led to cessation of exposure to risk during the pandemic. Among all women, the proportion at risk of unintended pregnancy at both time points (i.e., sustained risk) ranged from 20.5% in Kinshasa-DRC to 42.2% in Kenya. Among women with sustained risk, change in contraceptive status varied from 19.4% in Kenya to 25.2% in Burkina Faso. Contraceptive adoption was more common than discontinuation in Burkina Faso (16.9% versus 8.3%, respectively), Kenya (12.6% versus 6.8%, respectively) and Lagos-Nigeria (15.2% versus 8.7%, respectively) but equivalent in Kinshasa-DRC (11.5% versus 12.6%, respectively). Among women who changed contraceptive status, most adopted or discontinued less effective barrier and natural methods in Kinshasa-DRC (66.9% and 68.5%, respectively) and Lagos-Nigeria (77.8% and 74.1%, respectively), while most adopted or discontinued effective short-acting methods in Burkina Faso (45.1% and 44.7%, respectively) and Kenya (47.8% and 54.2%, respectively). Highly effective long-acting method contributed the least to adoption and discontinuation in Lagos-Nigeria (6.0% and 2.7%, respectively) and accounted for 27.0% of adoption and 39.5% of discontinuation in Burkina Faso. Patterns of change reflect differences in contraceptive method mix among women at risk before COVID-19, as 65.5% and 73.2% of contraceptive users relied on less effective methods in Kinshasa-DRC and Lagos-Nigeria, respectively, versus 15% in Kenya and 19% in Burkina Faso. Conversely, 44.7% of women at risk in Kenya and 47.8% in Burkina Faso relied on highly effective methods pre-pandemic versus 17.4% in Kinshasa-DRC and 19.0% in Lagos-Nigeria.

While results from GEE models identified few statistically significant interactions between time and women's characteristics, signaling limited differences in contraceptive trends by sociodemographic characteristics, some patterns emerged (Table 4). For example, increases in the odds of contraceptive use during COVID-19 relative to the pre-pandemic period were more pronounced among women in their mid-twenties compared to teenagers or women over 40 years in all sites but Kinshasa-DRC. Likewise, increases in the odds of contraceptive use were elevated among parous women but not among nulliparous women in Kenya. In Lagos-Nigeria, increases in the odds of contraceptive use were greater among women with 1-2 children relative to those who had more children. Finally, wealthiest women in Lagos experienced greater increases in the odds of contraceptive use relative to women in the medium wealth category in Lagos. Conversely, there were no differential trends by education, recent loss of income, or recent experience of food insecurity.

Table 4. Differential trends in contraceptive use by women's sociodemographic characteristics

	Burkina Faso (n=5,207)	Kenya (n=6,932)	DRC-Kinshasa (n=1,967)	Nigeria-Lagos (n=1,088)
	RRR (95% CI)			
Overall	1.4 (1.2, 1.6)	1.3 (1.2, 1.5)	0.9 (0.7, 1.2)	1.3 (1.1, 1.6)
Age#year interaction	p=0.06	p=0.24	p=0.13	p=0.08

15-19	1.3 (0.8, 2.2)	1.4 (0.7, 2.8)	0.4 (0.1,2.6)	-
20-24	1.4 (1.1, 1.8)	1.3 (1.0, 1.7)	1.5 (0.6,3.6)	1.1 (0.5, 2.2)
25-29	1.7 (1.2, 2.3)	1.5 (1.2, 1.9)	1.2 (0.7,2.2)	2.9 (1.5, 5.6)
30-34	1.2 (0.8, 1.6)	1.4 (1.2, 1.6)	0.4 (0.2,0.7)	1.7 (1.0, 3.1)
35-39	1.6 (1.2, 2.3)	1.5 (1.2, 1.8)	1.1 (0.6,1.8)	1.0 (0.7, 1.4)
40-44	1.5 (1.0, 2.1	1.1 (0.9, 1.3)	0.9 (0.6,1.4)	1.2 (0.7, 2.1)
45-49	0.8 (0.7, 1.1)	1.1 (0.9, 1.4)	1.0 (0.6,1.7)	1.1 (0.6, 2.0)
Parity#year interaction	p=0.34	p=0.08	p=0.88	p=0.004
Nulliparous	-	0.7 (0.2, 2.1)	3.3 (0.2, 55.9)	-
1-2	1.4 (1.1, 1.8)	1.5 (1.2, 1.8)	0.8 (0.5, 1.3)	2.1 (1.4, 23.0)
3+	1.4 (1.2, 1.6)	1.3 (1.1, 1.4)	1.0 (0.7, 1.2)	1.1 (0.8, 1.4)
Residence#year interaction	p=0.32	p=0.54		
Urban	1.3 (1.1, 1.4)	1.2 (1.0, 1.5)	--	--
Rural	1.4 (1.2, 1.6)	1.3 (1.2, 1.5)	--	--
Wealth#year interaction	p=0.57	p=0.61	p=0.42	p=0.08
Poorest	1.5 (1.2, 2.0)	1.4 (1.2, 1.6)	0.9 (0.5, 1.4)	1.5 (0.9, 2.3)
Medium	1.3 (1.0, 1.6)	1.3 (1.1, 1.6)	1.1 (0.7, 1.8)	1.1 (0.7, 1.6)
Richest	1.3 (0.9, 1.3)	1.2 (0.9, 1.5)	1.1 (0.7, 1.8)	2.0 (1.3, 2.9)
Loss of income last 12 months	p=0.30	p=0.48	p=0.71	p=0.09
None	-	1.2 (1.0, 1.4)	-	-
Partial	1.4 (1.2, 1.6)	1.4 (1.2, 1.5)	0.9 (0.7,1.3)	1.4 (1.1, 1.9)
Complete	1.5 (1.0, 2.3)	1.3 (1.1, 1.5)	0.9 (0.7, 1.2)	1.2 (0.8, 1.7)
Food insecurity	p=0.24	p=0.33	p=0.85	p=0.10
No	1.4 (1.2, 1.5)	1.3 (1.2, 1.4)	1.0 (0.7, 1.3)	1.4 (1.1, 1.7)
Yes	1.8 (1.1, 2.9)	1.5 (1.2, 1.9)	0.7(0.5, 1.1)	0.8 (0.6, 1.1)
Education	p=0.29	p=0.87	p=0.26	p=0.22
No schooling/primary	1.4 (1.2, 1.6)	1.3 (1.2, 1.4)	0.6 (0.3, 1.3)	1.2 (0.7, 2.0)
Secondary low	1.3 (0.8, 2.0)	1.2 (0.6, 2.4)	1.0 (0.8, 1.3)	1.2 (0.9, 1.7)
Secondary high or higher	0.7 (0.3,1.3)	1.3 (1.1,1.6)	0.7 (0.4,1.2)	1.9 (1.4,2.6)

Few factors were related to contraceptive adoption among women who were not using a method pre-pandemic and to contraceptive discontinuation among those using contraception pre-pandemic (Appendix 3). The odds of adopting contraception were higher among women with secondary education or higher in Burkina Faso and Kenya, relative to less educated women, and among women in the richest tertile, relative to the poorest women in Kenya. Conversely the oldest women (35-49 years) were less likely to adopt a method in Kinshasa-DRC and Kenya and, more likely to

For peer review only

For peer review only - http://bmjopen.bmj.com/site/about/guidelines.xhtml

discontinue their method in Kenya. Discontinuation was also more common among parous women compared to nulliparous women in Kenya. Finally, women with lower secondary education in Kinshasa were less likely to discontinue their method than women with no schooling or primary education.

Finally, factors associated with characterizing an unintended pregnancy during COVID-19 among women who had sexual intercourse in the last 12 months are reported in Appendix 4. Older women (35 years or older) and women using long-acting contraception were less likely to experience an unintended pregnancy across sites. Women with higher education and from wealthier households were less likely to experience an unintended pregnancy in Kenya, while women of higher parity were more likely to experience unintended pregnancies in Burkina Faso and Kinshasa-DRC. COVID-19-related economic vulnerability was unrelated to unintended pregnancy across sites.

DISCUSSION

This study confirms women's resiliency in contraceptive use to prevent unintended pregnancies across a diversity of sub-Saharan contexts, despite the profound economic and social disruptions caused by the COVID-19 pandemic [2-4,16-17]. While we found a sustained need for contraception, we also observed an increase in contraceptive coverage in three of the four geographies studied. Within Burkina Faso and Kenya, increases in contraceptive protection corresponded with a decrease in the use of methods requiring a prescription, which was only significant in rural Kenya, but most women still used effective or highly effective methods, including women who adopted methods during the pandemic. These contraceptive dynamics coincide with the absence of significant changes in pregnancy rates or unintended pregnancy rates across sites.

These results challenge initial forecasts of rising unintended pregnancies due to service disruptions to sexual and reproductive health services in LMICs [7]. The absence of negative population-level effects of the pandemic on contraceptive behaviors and reproductive outcomes, noted in a previous study focusing on the early months of the pandemic [14,15], are surprisingly reassuring and suggest several potential explanations [23].

First, while disruptions to sexual and reproductive health (SRH) services were reported in the early stage of the pandemic [5,23-28], patterns of service disruptions varied significantly by level, duration, type of service and geography [24-26]. Discontinuity of services was often short-lived—several studies reporting rapid post-lockdown rebounds in service provision [23,27-29].

Further, it is possible that the quality of facility-based registers, including clients' family planning records, were impacted due to understaffing amid COVID-19-related disruptions and, thus, early pandemic provision of care was underreported [30].

At the same time, the expansion or continued use of high impact practices in family planning [31], such as community health workers, mobile outreach, supply chain management, and digital health initiatives, may also have compensated for facility-based disruptions to care. For example, the expansion of community health workers to deliver family planning services resulted in a 2.5 percent increase in the number of family planning clients in rural Kenya in the summer of 2020 [32]. These initiatives were encouraged by the Kenyan government who issued guidelines to

For peer review only - http://bmjopen.bmj.com/site/about/guidelines.xhtml

support "Continuity of Reproductive, Maternal, Newborn and Family Planning Care and Services in the Background of COVID-19 Pandemic" in the month following the first reported case of COVID- 19 [33]. More generally, a recent WHO report of SRH services in Africa during COVID-19 indicates that 15 out the 17 countries reflected in the survey had integrated SRH services in their continuity of care plan during COVID-19, including awareness campaigns and communication messages about family planning [25].

A third explanation lies in the resilience of women in intensifying their efforts or finding alternative solutions to access contraception and self-manage their contraceptive needs [25]. This hypothesis is plausible given that our initial investigation into family planning amid COVID-19, conducted between May and July of 2020, found that only 3.8-14.4% of women at risk of unintended pregnancy were not using contraception for COVID-19-related reasons in Burkina Faso and Kenya; these women primarily cited fear of COVID-19 transmission [15]. Approximately six months later, the data collected via PMA surveys between November 2020 and January 2021 included in the present analysis reveal 9 to 35% reported difficulties accessing any health service in the last year, however 89-93% of these were ultimately successful in obtaining services (www.pmadata.org). Self-provision of oral contraceptive pills during the COVID-19 is likely to have facilitated women's access to short-acting contraception [24], while the impact of DMPA-SC self-administration policies should be further investigated to evaluate their contribution to continuity of family planning use during the pandemic.

These overall trends remain encouraging, but a critical concern over widening social disparities in family planning access may still arise in the face of increasing economic hardships [3-4]. Our findings provide no evidence of adverse SRH outcomes among women who report food insecurity, or those who experienced substantial loss of income since the start of the COVID-19 pandemic. Recent FP2030 policy commitments towards family planning (https://fp2030.org/FP2030-commitment-makers), including increased public funding, and expansion of free access to contraception in some countries, such as Burkina Faso in 2019 are likely to have mitigated the economic impact of COVID-19, but the affordability of contraception remains a prominent concern for women experiencing economic hardship [18].

Our results should be considered with several limitations in mind. Specifically, the loss to follow-up rate ranged from 21-27% across geographies, which is likely to affect the reported estimates of change. However, the use of replacement households in Phase 2 of data collection was designed to minimize impacts to population-level estimates. Further, post-stratification weights were used in panel analysis to limit the potential for attrition biases; stable estimates of sociodemographic distributions across samples were observed (Appendix 5). A second limitation is our measure of change using two time points, which prevents a more comprehensive assessment of changes over the full time-period, including potential gaps in contraceptive use. A further evaluation of contraceptive use dynamics, using calendar data, was beyond the scope of this analysis but could provide a more thorough investigation of contraceptive gaps, albeit limited by the quality of the calendar data [34]. Our data in DRC and Nigeria are not nationally representative but rather reflect urban trends. Stratified analyses comparing urban and rural Burkina Faso and Kenya found no major differences in family planning trends, however, these results cannot be generalized to DRC and Nigeria, given distinct patterns of SRH disruptions reported in several studies in Africa [25-26]. Finally, our sample sizes were limited to detect significant trends in rare events, such as

For peer review only - http://bmjopen.bmj.com/site/about/guidelines.xhtml

pregnancy outcomes, or differential trends by women's sociodemographic and economic background.

Our study also has several strengths. First, our population-based approach provides a complimentary perspective to data collected via Health Management Information Systems (HMIS), showing little change in behaviors and SRH outcomes, despite facility-based disruptions, suggesting the importance of non-facility-based services and self-care practices in ensuring continuity of family planning care [25]. Second, our novel study design, integrating perspectives from representative cross-sectional data and panel data, offers the double advantage of examining population and individual trends between pre- COVID-19 and COVID-19 periods, with the potential of identifying disparities in COVID-19 effects on family planning practices. The use of a comprehensive set of indicators ranging from pregnancy exposure to contraceptive practices and reproductive outcomes also provides a more holistic understanding of fertility processes during a pandemic, elucidating how family planning needs and use evolve concurrently. Finally, the cross-national/regional nature of the study, enhanced by using a common study protocol and indicators across geographies strengthens comparative trends analysis, supporting previously reported variations in COVID-19 effects on SRH services [25-26].

In conclusion, the current study provides important insights on the resilience of women across geographically and socially diverse contexts in sub-Saharan Africa in sustaining contraceptive practices amid the COVID-19 pandemic. Renewed national and international commitments to family planning made as part of the 2011 Ouagadougou Partnership [35] and 2012 London Summit on Family Planning [36] have likely contributed to the resiliency and innovations in family planning delivery systems. The integration of maternal health and other reproductive health services into continuity of care plans during COVID-19 is a reflection of such commitments [25]. However, investments in these systems must be sustained to ultimately reduce unsatisfied demand for contraception and achieve universal access to SRH services. With rising global poverty and the diversion of international family planning funding (as exemplified by the recent shortfall in UNFPA's funding for family planning programs), there is continued cause for concern over women's access to affordable contraceptive services in-sub-Saharan Africa. Sustained global and local advocacy and monitoring are needed to ensure accountability and progress towards essential SRH service provision, including women's access to and use of contraception.

Figure 1: Changes in individual level risk of unintended pregnancy, and use of contraception between Phases 1 and 2, and contraceptive method mix between adopters and discontinuers of contraception

Contributorship: FMO, PA, GG, PG, LZ and PAA designed the PMA panel study, CM, FMO, PA, GG, PG, LZ and PAA designed the COVID module, CM, CK, SW designed this particular study. CM conducted the analysis, CM and KW conducted the literature review, CM, KW, CK, SW wrote the initial draft, and FMO, PA, GG, PG, LZ, PAA interpreted the results and revised the manuscript.

Funding statement: This research was developed under grants OPP1198333 and OPP1198339 awarded by the Bill & Melinda Gates Foundation to Johns Hopkins Bloomberg School of Public Health and Jhpiego.

Competing Interests: None declared.

For peer review only - http://bmjopen.bmj.com/site/about/guidelines.xhtml

Ethical Approval

PMA surveys received approval from ethical committees in each geography (Burkina Faso: Comité d'Ethique Institutionnel Pour La Recherche en Santé, Ministère de l'Enseignement Supérieur, de la Recherche Scientifique et de l'Innovation; Democratic Republic of the Congo: University of Kinshasa School of Public Health; Kenya: Kenyatta National Hospital—University of Nairobi Ethics Research Committee; Nigeria: Lagos State University Teaching Hospital Health Research Ethics Committee). PMA also received approval from the Institutional review board of the Johns Hopkins School of Public Health (IRB00014702). Public health preventive measures (i.e., social distancing, mask-wearing, use of hand sanitizer) were instituted in Phase 2 to prevent COVID-19 transmission during data collection.

Data Availability Statement: Data are publicly available at pmadata.org.

REFERENCES

1 Our world data. Cumulative confoirmed covid-19 deaths by world reigion. https://ourworldindata.org/grapher/cumulative-covid-deaths-region

2 Congressional research Service. Global Economic Effects of COVID-19. Nov 2010. https://sgp.fas.org/crs/row/R46270.pdf

3 Selassie AA, Hakobyan S. six-charts-show-the-challenges-faced-by-sub-saharan-africa, IMF African Department, April 15, 2021 https://www.imf.org/en/News/Articles/2021/04/12/na041521-six-charts-show-the challenges-faced-by-sub-saharan-africa

4 Gummerson E, Cardona C, Anglewicz P, *et al.* The wealth gradient and the effect of COVID-19 restrictions on income loss, food insecurity and health care access in four sub-Saharan African geographies. *PLoS One* 2021;**16**:e0260823. doi:10.1371/journal.pone.0260823

5. International Planned Parenthood Federation (IPPF). Contraception and COVID-19: Disrupted supply and access. . https://www.ippf.org/blogs/contraception-and-covid-19-disrupted-supply-and-access. 2020

6. Endler M, Al-Haidari T, Benedetto C, Chowdhury S, Christilaw J, El Kak F, et al. How the coronavirus disease 2019 pandemic is impacting sexual and reproductive health and rights and response: results from a global survey of providers, researchers, and policy-makers. Acta Obstet Gynecol Scand. 2021;100(4):571–578.

7 Riley T, Sully E, Ahmed Z, *et al.* Estimates of the Potential Impact of the COVID-19 Pandemic on Sexual and Reproductive Health In Low- and Middle-Income Countries. *Int Perspect Sex Reprod Health* 2020;**46**:73–6.

8 UNFPA. Impact of the COVID-19 Pandemic on Family Planning and Ending Gender-based Violence, Female Genital Mutilation and Child Marriage. 2020. https://www.unfpa.org/sites/default/files/resource-pdf/COVID-19_impact_brief_for_UNFPA_24_April_2020_1.pdf

9 Hall KS, Samari G, Garbers S, *et al.* Centring sexual and reproductive health and justice in the global COVID-19 response. *Lancet* 2020;**395**:1175–7. doi:10.1016/S0140-6736(20)30801-1

10 Govender D, Naidoo S, Taylor M. Don't Let Sexual and Reproductive Health become Collateral Damage in the Face of the COVID-19 Pandemic: A Public Health Perspective. Afr J Reprod Health. 2020 Jun;24(s1):56-635 Wood SN, Karp C, OlaOlorun F, et

For peer review only

al. Trends in the need for and use of contraception before and during COVID-19 in four sub-Saharan African geographies: results from population-based national or regional cohort surveys. Lancet Glob Heal 2021;9:e793-801.

11 Hussain R et al., Unmet Need for Contraception in Developing Countries: Examining Women's Reasons for Not Using a Method, New York: Guttmacher Institute, 2016, http://www.guttmacher.org/report/unmet-need-for-contraception-in-develop....

12 Bearak JM, Popinchalk A, Beavin C, *et al*. Country-specific estimates of unintended pregnancy and abortion incidence: a global comparative analysis of levels in 2015–2019. *BMJ Global Health* 2022;**7:**e007151

13 United Nations Department of Economic and Social Affairs Population facts. July 2019. Global progress in satisfying the need for family planning https://www.un.org/en/development/desa/population/publications/pdf/popfacts/PopFacts_2019-3.pdf, Accessed 10th Mar 2021

14 Wood SN, Karp C, OlaOlorun F, *et al.* Trends in the need for and use of contraception before and during COVID-19 in four sub-Saharan African geographies: results from population-based national or regional cohort surveys. *Lancet Glob Heal* 2021;**9**:e793-801.

15 Karp C, Wood SN, Guiella G, *et al.* Contraceptive dynamics during COVID-19 in sub--Saharan Africa: longitudinal evidence from Burkina Faso and Kenya. *BMJ Sex Reprod Heal* 2021;**0**:1–9. doi:10.1136/bmjsrh-2020-200944

16 Mahler DG, Yonzan N, Lakner C, *et al.* Updated estimates of the impact of COVID-19 on global poverty: Turning the corner on the pandemic in 2021? World Bank Data Blog. 2021;:1–10.https://blogs.worldbank.org/opendata/updated-estimates-impact-covid-19-global-poverty-turning-corner-pandemic-2021

17 Our World in Data. Policy Responses to the Coronavirus Pandemic--Statistics and Research. https://ourworldindata.org/policy-responses-covid (accessed 26 Jan 2022).

18 Decker MR, Wood SN, Thiongo M, *et al.* Gendered health, economic, social and safety impact of COVID-19 on adolescents and young adults in Nairobi, Kenya. *PLoS One* 2021;**16**:e0259583.

19 Karp C, Moreau C, Sheehy G, *et al.* Youth Relationships in the Era of COVID-19: A Mixed-Methods Study Among Adolescent Girls and Young Women in Kenya. *J Adolesc Heal* 2021;**69**:754–61. doi:10.1016/j.jadohealth.2021.07.017

20 Adu, P.A., Stallwood, L., Adebola, S.O. *et al.* The direct and indirect impact of COVID-19 pandemic on maternal and child health services in Africa: a scoping review. *glob health res policy* **7,** 20 (2022). https://doi.org/10.1186/s41256-022-00257-z

21 Greenleaf AR, Gadiaga A, Guiella G, *et al.* Comparability of modern contraceptive use estimates between a face-to-face survey and a cellphone survey among women in Burkina Faso. *PLoS One* 2020;**15**:1–15. doi:10.1371/journal.pone.0231819

22 Backhaus, A. (2021, July 25). Pregnancies and contraceptive use in four African countries during the COVID-19 pandemic. https://doi.org/10.31235/osf.io/av7ec

23 Thomas AB, Yaya S. Looking ahead in the COVID - 19 pandemic : emerging lessons learned for sexual and reproductive health services in low- and middle- income countries. *Reprod Health* 2021;**4**:1–8.

24 Assefa N, Sié A, Wang D, Korte ML, Hemler EC, Abdullahi YY, et al. Reported barriers to healthcare access and service disruptions caused by COVID-19 in Burkina Faso, Ethiopia, and Nigeria: a telephone survey. Am J Trop Med Hygiene. 2021;1(aop).

For peer review only - http://bmjopen.bmj.com/site/about/guidelines.xhtml

25 Otieno, George & Ouedraogo, Leopold & Nkurunziza, Triphonie & Asmani, Chilanga & Elamin, Hayfa & Muriithi, Assumpta & Onyiah, Amaka & Conombo, Ghislaine & Kidula, Nancy & Tall, Fatim & Sekpon, Ameyo & Adegboyega, Ayotunde. (2021). Continuity of Essential Sexual and Reproductive Health Services During Covid-19 Pandemic in the Who African Region. 10.21203/rs.3.rs-316744/v1.

26 APHRC, 2022. *Impact of the COVID-19 Pandemic on Sexual and Reproductive Health Services in Burkina Faso, Ethiopia,*, Africa Population Health Research Center. Retrieved from https://policycommons.net/artifacts/2460145/impact-of-the-covid-19-pandemic-on-sexual-and-reproductive-health-services-in-burkina-faso-ethiopia/3481942/ on 28 Jul 2022. CID: 20.500.12592/qgq1pf.

27 Fuseini K, Jarvis L, Ankomah A, Bintou Mbow F, Hindin MJ. Did COVID-19 Impact Contraceptive Uptake? Evidence from Senegal. Stud Fam Plann. 2022 Jun;53(2):301-314. doi: 10.1111/sifp.12195. Epub 2022 Apr 18. PMID: 35436350; PMCID: PMC9115390.

28 Leight J, Hensly C, Chissano M, *et al.* Short-term effects of the COVID-19 state of emergency on contraceptive access and utilization in Mozambique. *PLoS One* 2021;**16**:1–16. doi:10.1371/journal.pone.0249195

29 Ameh C, Banke-Thomas A, Balogun M, *et al.* Reproductive maternal and newborn health Providers' assessment of facility preparedness and its determinants during the COVID-19 pandemic in Lagos, Nigeria. *Am J Trop Med Hyg* 2021;**104**:1495–506. doi:10.4269/ajtmh.20-1324

30 Karp C, Moreau C, Olaolorun F, *et al.* Family planning service disruptions amid COVID-19: Longitudinal evidence from facilities in seven low- and middle-income countries Authors. 2021. https://ipc2021.popconf.org/uploads/211072

31 Mickler AK, Carrasco MA, Raney L, *et al.* Applications of the High Impact Practices in Family Planning during COVID-19. *Sex Reprod Heal Matters* 2021;**29**. doi:10.1080/26410397.2021.1881210

32 PSI. Serving Sara during the COVID-19 pandemic: Lessons learned in family planning adapatations. 2021;:1–13.https://www.psi.org/wp-content/uploads/2021/08/PSI_COVID_Briefs_Final_29July2021.pdf

33 Government of Kenya M of H. Kenya COVID19 RMNH Guidelines: A Kenya Practical Guide for Continuity of Reproductive, Maternal, Newborn and Family Planning Care and Services in the Background of COVID19 Pandemic. 2020. https://kenya.unfpa.org/sites/default/files/pub-pdf/KENYA - COVID19 RMNH GUIDELINES.pdf

34 The Ouagadougou Partnership. 2021.https://partenariatouaga.org/en/about-us/the-partnership/ (accessed 27 Jan 2022).

35 Bradley SE., Sarnak D, Breckler E, *et al.* Measuring Dynamic Contraceptive Use: The Quality and Consistency of DHS Calendar Data. 2021.

36 The London Summit on Family Planning. 2012.

For peer review only - http://bmjopen.bmj.com/site/about/guidelines.xhtml

Figure 1: Changes in individual level risk of unintended pregnancy, and use of contraception between Phases 1 and 2, and contraceptive method mix between adopters and discontinuers of contraception

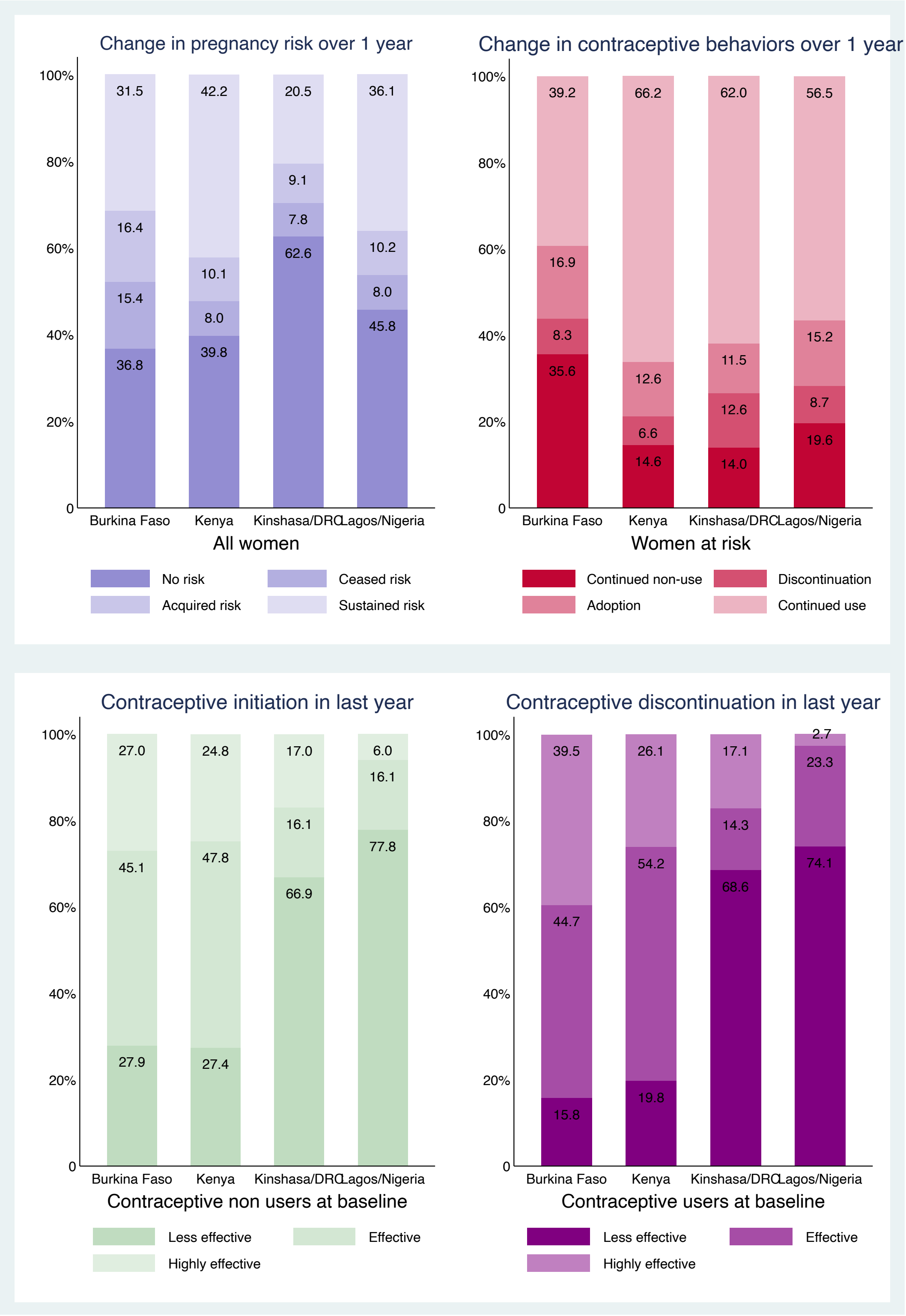

APPENDIX 1

The following GEE model was used to test for differential trends in the use of any contraception between Phase 1 and Phase 2, by women's sociodemographic characteristics

$\text{Logit}\{E(y_{it})\} = \beta_n x_{nit} * t$, $y \sim \text{Bernoulli}$

Y_{it} represents the response for each subject *i*, measured at different time points (*t*=1,2).
$Xn = (X_1, X_2, X_3, \ldots, Xn$ represent sociodemographic variables
x_n represents each sociodemographic variable
βn represents the slope of the outcome for variable x_n

We used a logit function as the outcome *y* (contraception use) is binary with a Bernoulli distribution. We used unstructured-free estimation to account for within-subject correlation

For peer review only - http://bmjopen.bmj.com/site/about/guidelines.xhtml

APPENDIX 2 Cross-sectional reproductive characteristics of women at Phase 1 and Phase 2, by urban and rural setting

	Urban Burkina Faso		Rural Burkina Faso		Urban Kenya		Rural Kenya		DRC-Kinshasa		Nigeria-Lagos	
	Phase 1 (n=)	Phase 2	Phase 1 (n=)	Phase 2	Phase 1 (n=)	Phase 2	Phase 1 (n=)	Phase 2	Phase 1 (n=2,611)	Phase 2	Phase 1 (n=1,469)	Phase 2
Intends to have a child in next year	18.6 (16.9, 20.6)	18.2 (16.8, 19.7)	11.9 (9.9, 14.3)	12.9 (10.8, 15.5)	8.4 (6.9, 10.1)	7.8 (6.5, 9.4)	6.3 (5.4, 7.4)	5.4 (4.7, 6.3)	12.2 (9.3, 15.8)	10.1 (7.8, 13.2)	16.8 (14.6, 19.2)	16.2 (14.6, 18.1)
In need of contraception	33.8 (31.9, 35.8)	35.5 (33.4, 37.7)	47.2 (43.4, 51.0)	47.1 (43.2, 51.0)	43.2 (40.9, 45.5)	43.7 (41.5, 45.9)	47.6 (46.0, 49.2)	46.4 (44.8, 48.1)	27.8 (24.7, 31.1)	29.2 (26.3, 32.2)	39.5 (36.6, 42.5)	40.4 (37.7, 43.2)
Contraceptive use, among women in need	61.5 (58.0, 64.9)	65.9 (62.8, 68.9)	39.4 (34.5, 44.5)	49.4 (44.3, 54.5)	79.1 (75.8, 82.0)	82.6 (79.8, 85.1)	68.2 (65.9, 70.5)	73.9 (71.6, 76.2)	71.8 (67.4, 75.9)	72.4 (68.2, 76.2)	63.4 (56.8, 69.5)	69.7 (63.7, 75.2)
Provider-dependent contraception, among contraceptive users	73.5 (69.5, 77.0)	68.7 (64.5, 72.7)	86.7 (81.3, 90.8)	82.1 (75.9, 87.0)	79.7 (75.8, 83.0)	80.1 (76.4, 83.3)	92.3 (90.8, 93.6)	89.1 (87.4, 90.6)	37.3 (29.5, 45.9)	35.6 (28.3, 43.5)	40.1 (33.7, 46.9)	35.2 (29.1, 41.8)
Currently pregnant	6 (5.2, 6.9)	6.1 (5.3, 7.0)	9.3 (8.1, 10.6)	8.1 (6.7, 9.6)	6.1 (5.1, 7.2)	4.2 (3.5, 5.1)	5.1 (4.5, 5.7)	5.5 (4.9, 6.2)	5.7 (4.5, 7.1)	5.2 (4.1, 6.5)	4.7 (3.7, 6.0)	4.7 (3.7, 5.9)
Current unintended pregnancy	1.6 (1.2,2.1)	1.2 (1.0,1.6)	3.1 (2.3,4.2)	2.3 (1.7,3.2)	2.2 (1.7,2.8)	1.5 (1.0,2.1)	2.3 (1.9,2.8)	2.3 (1.9,2.8)	2.6 (1.9,3.6)	2.8 (2.1,3.8)	1 (0.6-1.6)	1 (0.6.1.7)

For peer review only - http://bmjopen.bmj.com/site/about/guidelines.xhtml

APPENDIX 3. Sociodemographic factors related to contraceptive adoption and discontinuation between Phases 1 and 2--results of multivariable logistic regression models, per setting

	Burkina Faso		Kenya		Kinshasa-DRC		Lagos-Nigeria	
	Adoption (n=972)	Discontinu ation. (n=917)	Adoption (n=929)	Discontin uation. (n=2183)	Adoption (n=126)	Discontin uation. (n=321)	Adoption (n=156)	Discontin uation . (n=271)
	aOR (95% CI)							
Age								
15-24	ref	ref	ref	ref	ref	ref	ref	ref
25-34	1.3 (0.7-2.5)	**3.7 (1.1,12.0)**	0.7 (0.4-1.3)	1.2 (0.6-2.32)	0.5 (0.1-2.7)	2.1 (0.5-9.0)	-	1.0 (0.2-6.6)
35-49	1.0 (0.4-2.3)	2.9 (0.8-11.1	**0.4 (0.2-0.8)**	**2.0 (1.1-3.9)**	**0.1 (0.0-0.8)**	0.9 (0.2-4.7)	3.9 (0.3-51.3)	3.0 (0.4-24.7
Parity								
0-1	ref	ref	ref	ref	ref	ref	ref	ref
2-3	2.0 (1.0-3.9)	0.4 (0.1-1.6)	1.9 (1.0-3.8)	**0.4 (0.3-0.8)**	2.1 (0.10.4)	0.4 (0.2-1.6)	0.3 (0.0-2.3)	0.5 (0.1-1.9)
4+	1.6 (0.6-4.8)	0.9 (0.3-3.3)	1.4 (0.7-2.9)	**0.5 (0.2-0.9)**	1.3 (0.2-7.1)	0.6 (0.2-1.6)	0.6 (0.4-6.1)	0.3 (0.1-1.)
Residence								
Urban	1.3 (0.7-2.3)	0.8 (0.3-2.-2)	1.1 (0.7-1.6)	0.8 (0.5-1.3)	--	--	--	--
Rural	ref	ref	ref	ref	--	--	--	
Education								--
No schooling /primary	ref	ref	ref	ref	ref	ref	ref	ref
Secondary low	**6.0 (2.8-12.8)**	1.6 (0.6-4.5)	1.1 (0.5-2.8)	0.6 (0.1-2.2)	1.3 (0.2-7.1)	**0.4 (0.2-1.0)**	1.6 (0.4-6.1)	0.6 (0.2-2.0)
Secondary high	**22.6 (4.4-116.7)**	0.9 (0.3-3.3)	**2.0 (1.3-3.0)**	1.2 (0.8-1.7)	0.8 (0.1-7.5)	0.6 (0.2-2.7)	3.5 (0.8-15.4)	0.4 (0.1-2.0)
Food insecurity								
Yes	0.8 (0.3-2.3)	1.1 (0.3,3.6)	0.9 (0.6-1.5)	1.2 (0.6-2.1)	0.3 (0.1-1.3)	0.7 (0.3-1.5)	**-**	0.4 (0.0-3.8)
No	ref	ref	ref	ref	ref	ref	ref	ref
Wealth								
Poorest	ref	ref	ref	ref	ref	ref	ref	ref
Medium	0.7 (0.4-1.2)	0.7 (0.4,1.3)	1.4 (0.9-2.0)	1.0 (0.6-1.5)	0.8 (0.2-2.9)	0.9 (0.4-2.1)	0.7 (0.2-2.46	0.8 (0.3-2.2)
Richest	0.8 (0.4-1.5)	0.5 (0.2-1.6)	**2.0 (1.2-3.4)**	1.0 (0.6-1.7)	0.9 (0.2-3.1)	0.6 (0.2-1.4)	1.2 (0.4-3.9)	0.6 (0.2-2.1)

For peer review only

APPENDIX 4. Sociodemographic factors related to having an unintended pregnancy in Phase 2--results of multivariable logistic regression models, per setting

	Burkina Faso N=4018	Kenya N=5616	Kinshasa-DRC N=1489	Lagos-Nigeria N=836
Age				
15-24	ref	ref	ref	ref
25-34	**0.2 (0.1-0.5)**	0.7 (0.5-1.2)	0.4 (0.2-1.1)	0.3 (0.1,1.0)
35-49	**0.1 (0.0-0.4)**	**0.1 (0.1-0.3)**	**0.1 (0.0-0.3)**	**0.1 (0.0-0.3)**
Marital status				
Not in a cohabitating union	ref	ref	ref	ref
Married or in union	1.1 (0.4-3.1)	0.7 (0.45-1.2)	**2.4 (1.1-5.1)**	2.9 (0.4-21.6)
Parity				
0-1	ref	ref	ref	ref
2-3	1.6 (0.7-3.7)	1.2 (0.7-2.2)	**3.6 (1.4-8.9)**	1.3 (0.1-10.9)
4+	**5.1 (1.3-19.9)**	1.4 (0.6-3.0)	**3.7 (1.2-11.4)**	0.8 (0.0-21.2)
Residence				
Urban	0.9 (0.4-1.9)	0.9 (0.4-1.6)	--	--
Rural	ref	ref	--	--
Education				
No schooling /primary	ref	ref	ref	ref
Secondary low	1.6 (0.8-3.2)	0.4 (0.1-1.6)	0.5 (0.2-1.3)	0.6 (0.1-4.3)
Secondary high	0.6 (0.2-2.0)	**0.4 (0.2-0.7)**	0.6 (0.1-3.2)	0.3 (0.0-3.5)
Food insecurity				
Yes	1.6 (0.7-3.8)	1.3 (0.7-2.2)	0.9 (0.6-2.0)	**-**
No	ref	ref	ref	
Wealth				
Poorest	ref	ref	ref	ref
Medium	1.4 (0.8-2.7)	**0.6 (0.3-0.9)**	**0.4 (0.2-1.0)**	0.3 (0.0-2.1)
Richest	0.8 (0.3-2.0)	**0.4 (0.2-0.8)**	0.5 (0.2-1.2)	1.1 (0.2-5.1)
Contraception at phase 1				
None	ref	ref	ref	ref
Less effective (barrier/natural)	1.0 (0.3-2.8)	1.4 (0.8-2.7)	1.1 (0.6,5.1)	0.3 (0.0,1.4)
Effective (short-acting hormonal)	1.4 (0.7-2.9)	1.1 (0.6-1.9)	**1.8 (0.2-0.8)**	0.4 (0.0-5.3)
Highly effective (long-acting methods)	**0.1 (0.0-0.2)**	**0.4 (0.2-0.8)**	**0.1 (0.0-0.3)**	**0.1 (0.0-0.8)**

± Less effective=barrier or natural methods; effective= short-acting hormonal methods, including pills, injectables, patches, or rings; highly effective=IUDs, implants, sterilization

For peer review only

APPENDIX 5. Sociodemographic characteristics by sample and setting, weighted

	Burkina Faso			Kenya			Kinshasa-DRC			Lagos-Nigeria		
Overall	Phase 1[a]	Phase 2	Panel	Phase 1	Phase 2	Panel	Phase 1	Phase 2	Panel	Phase 1	Phase 2	Panel
Age												
15-19	21.6	20.9	20.9	21.6	22.3	21.9	22.2	23.2	22.3	14.0	15.8	13.4
20-24	17.3	18.4	17.4	17.6	17.4	17.7	20.9	20.4	21.4	14.0	12.6	12.8
25-29	14.7	15.0	14.8	16.3	15.5	16.3	16.3	15.2	16.0	15.5	16.0	15.7
30-34	15.6	15.2	16.1	15.4	15.6	15.1	12.3	13.4	12.3	17.7	16.1	18.1
35-39	12.7	12.9	12.6	11.8	11.6	11.8	10.7	10.8	10.7	17.8	16.8	18.4
40-44	10.9	10.9	11.1	9.5	9.6	9.4	10.4	10.2	10.2	12.7	12.8	13.0
45-49	7.2	6.9	7.1	7.8	8.1	7.9	7.2	6.8	7.2	8.3	10.0	8.5
Parity												
Nulliparous	24.3	22.7	23.2	28.3	29.2	28.5	41.9	41.4	41.7	35.2	36.5	32.9
1-2	24.8	24.9	24.9	30.9	30.1	29.9	28.1	28.7	28.7	29.5	28.8	30.4
3+	50.9	52.5	51.9	40.9	40.7	41.6	30.1	30.0	29.7	35.3	34.7	36.7
Residence												
Urban	22.8	22.3	22.9	30.2	30.4	30.5	100.0	100.0	100.0	100.0	100.0	100.0
Rural	77.2	77.7	77.1	69.8	69.6	69.5	--	0.0	0.0	0.0	0.0	0.0
Wealth Tertile												
Poorest	330	33.5	33.1	35.9	34.6	35.8	33.9	31.7	33.6	34.2	33.0	34.7
Middle	33.5	32.9	33.2	33.5	34.2	33.6	32.3	33.0	32.7	30.7	33.0	30.8
Richest	33.5	33.7	33.7	30.6	31.3	30.6	33.8	35.4	33.8	35.1	34.0	34.6
Loss of income last 12 months												
None		0.0	0.0		22.4	21.1		0.0	0.0		0.0	0.0
Complete		9.2	9.7		25.2	25.0		41.4	41.7		29.0	32.5
Partial		90.8	90.3		52.4	53.9		58.6	58.3		71.0	67.6
Food insecurity												
No		93.8	93.9		88.1	88.9		79.5	81.0		92.0	92.4
Yes		6.2	6.1		11.9	11.1		20.5	19.0		8.0	7.6
Education												
None/primary	77.6	75.7	77.7	49.7	47.5	49.2	7.8	6.7	7.8	11.8	11.5	12.1
Secondary low	16.4	17.3	16.1	2.1	1.6	2.0	72.9	75.1	72.9	51.2	52.8	51.3
Secondary high+	6.1	7.0	6.2	48.2	50.8	48.8	19.3	18.2	19.3	37.0	35.7	36.6
Contraception												
None	67.7	70.1	68.5	50.4	52.2	52.2	57.1	55.4	55.1	60.2	60.6	60.2
Less effective	7.8	5.7	5.8	9.7	7.2	7.2	31.3	32.7	32.3	27.1	25.8	25.6
Effective	10.3	9.9	10.4	20.5	19.2	19.6	4.3	4.2	10.4	6.2	6.1	6.7
Highly effective	14.0	14.3	15.3	19.4	21.4	21.2	7.3	7.8	15.3	6.5	7.5	7.5

± Less effective=barrier or natural methods; effective= short-acting hormonal methods, including pills, injectables, patches, or rings; highly effective=IUDs, implants, sterilization

For peer review only - http://bmjopen.bmj.com/site/about/guidelines.xhtml

STROBE Statement—Checklist of items that should be included in reports of ***cross-sectional studies***

	Item No	Recommendation	Pg. & Line
**Title and abstract**	1	(*a*) Indicate the study's design with a commonly used term in the title or the abstract	1
		(*b*) Provide in the abstract an informative and balanced summary of what was done and what was found	3
**Introduction**			
Background/rationale	2	Explain the scientific background and rationale for the investigation being reported	4
Objectives	3	State specific objectives, including any prespecified hypotheses	4
**Methods**			
Study design	4	Present key elements of study design early in the paper	4
Setting	5	Describe the setting, locations, and relevant dates, including periods of recruitment, exposure, follow-up, and data collection	4
Participants	6	(*a*) Give the eligibility criteria, and the sources and methods of selection of participants	5
Variables	7	Clearly define all outcomes, exposures, predictors, potential confounders, and effect modifiers. Give diagnostic criteria, if applicable	6-7
Data sources/ measurement	8*	For each variable of interest, give sources of data and details of methods of assessment (measurement). Describe comparability of assessment methods if there is more than one group	*6-7*
Bias	9	Describe any efforts to address potential sources of bias	7
Study size	10	Explain how the study size was arrived at	4
Quantitative variables	11	Explain how quantitative variables were handled in the analyses. If applicable, describe which groupings were chosen and why	7
Statistical methods	12	(*a*) Describe all statistical methods, including those used to control for confounding	7
		(*b*) Describe any methods used to examine subgroups and interactions	7
		(*c*) Explain how missing data were addressed	
		(*d*) If applicable, describe analytical methods taking account of sampling strategy	7
		(*e*) Describe any sensitivity analyses	
**Results**			
Participants	13*	(a) Report numbers of individuals at each stage of study—eg numbers potentially eligible, examined for eligibility, confirmed eligible, included in the study, completing follow-up, and analysed	7-8
		(b) Give reasons for non-participation at each stage	6
		(c) Consider use of a flow diagram	
Descriptive data	14*	(a) Give characteristics of study participants (eg demographic, clinical, social) and information on exposures and potential confounders	8
		(b) Indicate number of participants with missing data for each variable of interest	9-10
Outcome data	15*	Report numbers of outcome events or summary measures	10

For peer review only - http://bmjopen.bmj.com/site/about/guidelines.xhtml

Main results	16	(*a*) Give unadjusted estimates and, if applicable, confounder-adjusted estimates and their precision (eg, 95% confidence interval). Make clear which confounders were adjusted for and why they were included	11-12
		(*b*) Report category boundaries when continuous variables were categorized	
		(*c*) If relevant, consider translating estimates of relative risk into absolute risk for a meaningful time period	
Other analyses	17	Report other analyses done—eg analyses of subgroups and interactions, and sensitivity analyses	
**Discussion**			
Key results	18	Summarise key results with reference to study objectives	12
Limitations	19	Discuss limitations of the study, taking into account sources of potential bias or imprecision. Discuss both direction and magnitude of any potential bias	13
Interpretation	20	Give a cautious overall interpretation of results considering objectives, limitations, multiplicity of analyses, results from similar studies, and other relevant evidence	12-13
Generalisability	21	Discuss the generalisability (external validity) of the study results	13
**Other information**			
Funding	22	Give the source of funding and the role of the funders for the present study and, if applicable, for the original study on which the present article is based	1

*Give information separately for exposed and unexposed groups.

Note: An Explanation and Elaboration article discusses each checklist item and gives methodological background and published examples of transparent reporting. The STROBE checklist is best used in conjunction with this article (freely available on the Web sites of PLoS Medicine at http://www.plosmedicine.org/, Annals of Internal Medicine at http://www.annals.org/, and Epidemiology at http://www.epidem.com/). Information on the STROBE Initiative is available at www.strobe-statement.org.

For peer review only

BMJ Open

Trends in fertility intentions and contraceptive practices in the context of COVID-19 in sub-Saharan Africa: Insights from four national and regional population-based cohorts

Journal:	*BMJ Open*
Manuscript ID	bmjopen-2022-062385.R2
Article Type:	Original research
Date Submitted by the Author:	23-Sep-2022

Complete List of Authors:	Moreau, Caroline; Johns Hopkins University Bloomberg School of Public Health, Department of Population Family and Reproductive Health; INSERM, Soins et Santé Primaire, CESP Centre for Research in Epidemiology and Population Health U1018<br>Karp, Celia; Johns Hopkins University Bloomberg School of Public Health, Population Family and Reproductive Health<br>Wood, Shannon; Johns Hopkins University Bloomberg School of Public Health, Population Family and Reproductive Health<br>Williams, Kelsey; Johns Hopkins University Bloomberg School of Public Health, Department of Population Family and Reproductive Health<br>Olaolorun, Funmilola; University of Ibadan College of Medicine<br>Akilimali, Pierre; School of Public Health, University of Kinshasa<br>Guiella, Georges; Universite de Ouagadougou, Institut Supérieur des Sciences de la Population<br>Gichangi, Peter; International Centre for Reproductive Health Kenya<br>Zimmerman, Linnea; Johns Hopkins University Bloomberg School of Public Health, Population Family and Reproductive Health<br>Anglewicz, Philip; Johns Hopkins University, Population, Family, and Reproductive Health

**Primary Subject Heading**:	Public health
Secondary Subject Heading:	Sexual health
Keywords:	COVID-19, GYNAECOLOGY, REPRODUCTIVE MEDICINE

SCHOLARONE™ Manuscripts

BMJ

I, the Submitting Author has the right to grant and does grant on behalf of all authors of the Work (as defined in the below author licence), an exclusive licence and/or a non-exclusive licence for contributions from authors who are: i) UK Crown employees; ii) where BMJ has agreed a CC-BY licence shall apply, and/or iii) in accordance with the terms applicable for US Federal Government officers or employees acting as part of their official duties; on a worldwide, perpetual, irrevocable, royalty-free basis to BMJ Publishing Group Ltd ("BMJ") its licensees and where the relevant Journal is co-owned by BMJ to the co-owners of the Journal, to publish the Work in this journal and any other BMJ products and to exploit all rights, as set out in our licence.

The Submitting Author accepts and understands that any supply made under these terms is made by BMJ to the Submitting Author unless you are acting as an employee on behalf of your employer or a postgraduate student of an affiliated institution which is paying any applicable article publishing charge ("APC") for Open Access articles. Where the Submitting Author wishes to make the Work available on an Open Access basis (and intends to pay the relevant APC), the terms of reuse of such Open Access shall be governed by a Creative Commons licence – details of these licences and which Creative Commons licence will apply to this Work are set out in our licence referred to above.

Other than as permitted in any relevant BMJ Author's Self Archiving Policies, I confirm this Work has not been accepted for publication elsewhere, is not being considered for publication elsewhere and does not duplicate material already published. I confirm all authors consent to publication of this Work and authorise the granting of this licence.

For peer review only

Trends in fertility intentions and contraceptive practices in the context of COVID-19 in sub-Saharan Africa: Insights from four national and regional population-based cohorts

Caroline Moreau[1,2]
Celia Karp[1]
Shannon N. Wood[1]
Kelsey Williams[1]
Funmi M. OlaOlorun[3]
Pierre Akilimali[4]
Georges Guiella[5]
Peter Gichangi[6,7]
Linnea A. Zimmerman[1]
Philip A. Anglewicz[1]

[1] Department of Population Family and Reproductive Health, Johns Hopkins Bloomberg School of Public Health, Baltimore, Maryland, USA
[2] Soins et Santé Primaire, CESP Centre for Research in Epidemiology and Population Health U1018, Inserm, F-94805 Villejuif, France
3 College of Medicine, University of Ibadan, Ibadan, Nigeria
4 Kinshasa School of Public Health, Kinshasa, DRC.
5 Institut Supérieur des Sciences de la Population (ISSP/University of Ouagadougou), Ouagadougou, Burkina Faso
6 International Centre for Reproductive Health-Kenya, Nairobi, Kenya, Technical University of Mombasa;
7 Department of Public Health and Primary Care, Faculty of Medicine and Health Sciences, Ghent University, Belgium;

Corresponding author
Caroline Moreau, MD, PhD
Department of Population, Family and Reproductive Health
Johns Hopkins Bloomberg School of Public Health
615 N. Wolfe Street Rm. E4537 Baltimore, MD USA 21205
cmoreau2@jhu.edu

Word count: 3,584/5,000
Figures: 1
References: 23
Tables: 4 + 3 in Appendix

For peer review only

ABSTRACT

Objectives: Studies in several sub-Saharan geographies conducted early in the COVID-19 pandemic suggested little impact on contraceptive behaviors. Initial results may mask widening disparities with rising poverty, and changes to women's pregnancy desires and contraceptive use amid prolonged health service disruptions. This study examined trends in contraceptive behaviors in four sub-Saharan African settings one year into the pandemic.

Design: Nationally and regionally representative longitudinal surveys

Setting: Burkina Faso, Kenya, Democratic Republic of Congo (Kinshasa), and Nigeria (Lagos)

Participants: Women 15-49 years with sample size ranging from 1,469 in Nigeria to 9,477 in Kenya.

Outcome measures: Fertility preferences, contraceptive use, and unintended pregnancies measured before COVID-19 (November 2019-January 2020) and during COVID-19 (November 2020-January 2021).

Analysis: We described population-level and individual-level changes by socioeconomic characteristics using generalized equation modeling. We used logistic regression models to identify factors related to contraceptive adoption and discontinuation and to experiencing an unintended pregnancy.

Results: At the population level, we found no change in women's exposure to unintended pregnancy risk, alongside 5 to 9 percentage point increases in contraceptive prevalence in Burkina Faso, Kenya, and Lagos. Reliance on provider-dependent methods dropped by 2 and 4 percentage points in Kenya and Burkina Faso, respectively, although these declines were not statistically significant. Between 1.0% and 2.8% of women across sites experienced an unintended pregnancy during COVID-19, with no significant change over time. Individual-level trajectories showed contraceptive adoption was more common than discontinuation in Burkina Faso, Kenya, and Lagos, with little difference by sociodemographic characteristics. Women's COVID-19-related economic vulnerability was unrelated to unintended pregnancy across sites.

Conclusions:
This study highlights the resilience of African women across diverse settings in sustaining contraceptive practices amid the COVID-19 pandemic. However, with reports of rising poverty in sub-Saharan Africa, there is continued need to monitor access to essential sexual and reproductive health services.

For peer review only

STRENGTHS AND LIMITATIONS

- The study uses a longitudinal design to assess individual change in contraceptive behaviors since the onset of the COVID-19 pandemic.
- Nationally or regionally representative survey data were used to evaluate population level change in contraceptive behaviors since the onset of the COVID-19 pandemic.
- This investigation spans four diverse geographies in sub-Saharan Africa, allowing for a comparison of COVID-19 impact by social and pandemic contexts.
- Rates of loss to follow-up ranged from 21-27%, likely affecting study estimates, although the use of post-stratification weights reduces attrition biases.
- Data from two time points prevents the estimation of gaps in contraceptive use over the entire COVID-19 pandemic.

For peer review only

INTRODUCTION

The COVID-19 pandemic, which started in late 2019, quickly traversed the globe, creating health and economic crisis. By the end of 2020, the SARS-CoV-2 virus had resulted in nearly two million deaths [1], destroyed 114 million jobs [2], and debilitated global economic growth rates, which fell to an annualized negative rate of -3.2 [2]. With 65,468 confirmed fatalities, the sub-Saharan continent bore less of a health burden in this early stage of the pandemic [1], but swift policy containment measures led to unprecedented economic loss, with a 1.9% contraction in the Gross Domestic Product (GDP) per capita and an estimated 32 million additional people living in extreme poverty [3].

Loss of household income [4] coupled with restricted access to essential health services , including family planning [5-6] were expected to significantly alter fertility intentions and behaviors, [7–9], especially in sub-Saharan Africa [10], given pre-existing high unmet need for sexual and reproductive health (SRH) services [11]. In 2019, the unintended pregnancy rate (91 per 1000 women per year) in sub-Saharan Africa was the highest in the world [12] and only 55% of women's need for contraception was satisfied [13]. However, initial empirical evidence from four sub-Saharan African countries, Burkina Faso, Democratic Republic of Congo (DRC), Kenya, and Nigeria, using Performance monitoring for Action (PMA) data, suggested little impact on women's use of contraception in the early stages of the pandemic [14]. In fact, the study found a rise in contraceptive prevalence in rural Burkina Faso and across Kenya. These data were corroborated with findings further exploring changes in women's individual-level contraceptive behaviors in the early months of the pandemic in Burkina Faso and Kenya, indicating that more women adopted contraception than discontinued their methods between the end of 2019 and mid-2020 (13% Burkina Faso and 25% Kenya versus 5% Burkina Faso and 6% Kenya, respectively) [15].

While these initial studies were pivotal in reporting early pandemic trends, the longer term societal impacts of COVID-19, including rising poverty [3,16] and interruptions to schooling and employment [17], were likely to affect relationships and childbearing decisions [18-19]. At the same time, growing economic disparities were projected to affect people's financial access and use of health services [4,20], potentially reducing their ability to achieve their reproductive goals. Furthermore, early impact estimates were drawn from phone-based surveys amid COVID-19 lockdowns, which may have affected the interpretation of early study findings due to selection bias and data collection mode [14,15,21]. Recent analysis estimating changes in modern contraception and pregnancy rates using repeated cross-sectional surveys collected face to face shed light on these trends. Specifically, in Burkina Faso, Kenya, Kinshasa (DRC), and Lagos (Nigeria), data collected in pre-pandemic periods and one year into the pandemic, suggest slight decreases in pregnancy rates among younger, less educated women in Burkina Faso and a rise in modern contraception among young women in all geographies, except Lagos [22].

Expanding on these results, the present study describes population- and individual-level changes in women's fertility preferences, contraceptive behaviors, and unintended pregnancy experiences in the first year of the COVID-19 pandemic using cross-sectional representative data and panel data collected in the same sub-Saharan African geographies. Specifically, we examined how these trends differed by sociodemographic characteristics and COVID-19-related economic hardship.

For peer review only - http://bmjopen.bmj.com/site/about/guidelines.xhtml

Finally, we assessed factors related to individual-level changes in contraceptive behaviors and to experiencing an unintended pregnancy during the pandemic.

METHODS

Samples

We used two rounds of publicly available data (available at www.pmadata.org) collected in November 2019-January 2020 (3-4 months before the pandemic) and one year later (November 2020-January 2021) from nationally or regionally representative, population-based surveys [23]. Surveys were implemented by the Performance Monitoring for Action (PMA) project among women aged 15-49 in four sub-Saharan African geographies. Surveys are nationally representative in Burkina Faso and Kenya, and regionally representative in Kinshasa province in DRC and Lagos State in Nigeria. We did not include data from Kongo Central in the DRC and Kano State in Nigeria, also collected through the PMA platform due to smaller sample size and lack of statistical power to show any reduction in contraceptive use in these two geographies. Women were identified using a multistage clustered sampling design, starting with the random selection of census enumeration areas (EAs) after stratifying by rural and urban area (in Kenya and Burkina Faso), followed by the random selection of 35 households per EA. All women of reproductive age (15-49 years) from the selected households were eligible for inclusion. Sample sizes were determined to produce modern contraceptive prevalence rates (mCPRs) with a precision of +/- 3% at the national or regional level, leading to sample size variation by geography (Table 1).

Patient and Public involvement

This study is a population-based study that does not include patients. The public was not involved in this research.

Data collection

Pre-pandemic data (PMA's Phase 1 surveys) were collected face-to-face by trained resident interviewers before COVID-19 (November 2019-January 2020). Women who consented to be followed-up as part of PMA's panel design were re-interviewed in-person, one year later (PMA's Phase 2 surveys), during the pandemic (November 2020-January 2021). Replacement households from the EA were also included in Phase 2 to account for attrition at the household level (projected at 15%). The survey design allows for longitudinal analysis to examine individual-level changes since the onset of COVID-19 and for annual cross-sectional analysis to track national or regional level indicators (including household replacements). Follow-up rates between Phase 1 and Phase 2 surveys ranged from 73% in Kenya to 79% in Burkina Faso (Table 1). No differences between Phase 1 and Phase 2 cross-sectional sample characteristics were observed; differential lost to follow-up in the panel sample was accounted for by applying post-stratification weights (more details available at www.pmadata.org).

For peer review only - http://bmjopen.bmj.com/site/about/guidelines.xhtml

Table 1. Number of women aged 15 to 49 years included in each PMA survey and follow-up rate, by setting

Country/Site	Phase 1-Baseline	Phase 2 cross-sectional-including household replacement	Phase 2 panel-no replacement sample
Burkina Faso	6,590	6,362	5,207 (79%)
Kenya	9,477	9,310	6,932 (73%)
Kinshasa, DRC	2,611	2,368	1,967 (75%)
Lagos, Nigeria	1,469	1,481	1,088 (74%)

DRC=Democratic Republic of Congo.
Follow-up rate reported as % of all baseline women who successfully completed the Phase 2 survey

Ethical Approval

PMA surveys received approval from ethical committees in each geography (Burkina Faso: Comité d'Ethique Institutionnel Pour La Recherche en Santé, Ministère de l'Enseignement Supérieur, de la Recherche Scientifique et de l'Innovation; Democratic Republic of the Congo: University of Kinshasa School of Public Health; Kenya: Kenyatta National Hospital—University of Nairobi Ethics Research Committee; Nigeria: Lagos State University Teaching Hospital Health Research Ethics Committee). PMA also received approval from the Institutional review board of the Johns Hopkins School of Public Health (IRB00014702). Public health preventive measures (i.e., social distancing, mask-wearing, use of hand sanitizer) were instituted in Phase 2 to prevent COVID-19 transmission during data collection.

Measures

At each survey, women were asked about their current pregnancy status, whether the pregnancy was wanted or happened at the right time, future fertility intentions, and current contraceptive behaviors. This information was operationalized as indicators used for population-level comparisons (cross-sectional measures) and for individual-level trends analysis (longitudinal measures).

Cross-sectional measures

We explored six measures reflecting women's sexual and reproductive health experiences before and during the pandemic. *Women's fertility intentions for the next year* distinguished women who intended to have a child in the next year from those who wanted no more children or wanted to delay childbearing for more than one year. *Exposure to the risk of unintended pregnancy* (yes/no) classified women who had sexual intercourse in the last 12 months, were fecund, and were not pregnant or trying to have a child in the next year as being "at risk". Among women at risk of unintended pregnancy, *use of contraception* (yes/no) classified women who were using barrier and natural methods, pills, injectables, patches, rings, implants, IUDs, and sterilization as using contraception. Among contraceptive users, *use of a provider-dependent method* (yes/no) classified women using methods generally requiring interaction with a healthcare provider (i.e., pills, injectables, implants, IUDs, sterilization) as provider-dependent method users. We also considered a three-category measure of contraceptive method types, grouping contraceptive users according to the effectiveness of their contraceptive method—users of *less effective* methods (i.e., barrier or

For peer review only - http://bmjopen.bmj.com/site/about/guidelines.xhtml

natural methods), users of *effective* methods (i.e., short-acting hormonal methods, including pills, injectables, patches, or rings), and users of *highly effective* methods (i.e., implants, IUDs, or sterilization). *Pregnancy status* identified women who were pregnant at the time of each survey (yes/no), while an indicator of *unintended* (yes/no) identified women who were pregnant and indicated they had not wanted to become pregnant or wanted to become pregnant later.

Longitudinal measures
We also explored individual-level changes in women's *exposure to risk of unintended pregnancy*, *contraceptive use*, and *experience of an unintended pregnancy* (reported as wanted later or not at all). Changes in women's exposure to unintended pregnancy risk across the two surveys were categorized into four categories: 1) *No risk*—never at risk before or during COVID-19; 2) *Acquired risk*—newly at risk during COVID-19 (no risk pre-pandemic); 3) *Ceased risk*—no longer at risk during COVID-19 (at risk pre-pandemic); 4) *Sustained risk*—at risk before and during COVID-19. Likewise, we defined a four-category variable describing trends in any contraceptive use: 1) *Continued non-use*—non-use before or during COVID-19; 2) *Adoption*— during COVID-19 (non-use pre-pandemic); 3) *Discontinuation*—non-use during COVID-19 following use pre-pandemic; 4) *Continued use*—use before and during COVID-19. Finally, we considered the three-category indicator of contraceptive effectiveness (less effective/effective/highly effective) and a binary indicator of unintended pregnancy (yes/no) during Phase 2 (i.e., at the time of the COVID-19 survey), as previously defined.

We considered several sociodemographic and COVID-19-related factors that could impact women's use of contraception. Specifically, we assessed age, parity, marital status (in union/ not in union), education, household wealth in tertiles, and residence (urban/rural). During COVID-19, we also considered two indicators of household economic vulnerability: loss of income in the last 12 months (categorical—none, partial, complete), and food insecurity (i.e., not being able to eat for 24 hours due to lack of food since the beginning of the COVID-19 pandemic).

Analysis
We first examined population-level trends in reproductive health indicators comparing Phase 1 and Phase 2 cross sectional samples across our six indicators (proportion who intend to have a child in the next year, proportion at risk of unintended pregnancy, proportion using contraception among women who were at risk of unintended pregnancy, proportion using provider-dependent methods among users, proportion pregnant, and proportion experiencing an unintended pregnancy. We conducted the same analysis stratified by rural and urban residence in Burkina Faso and Kenya, to compare trends across urban localities (urban Burkina Faso, urban Kenya, Lagos_Nigeria and Kinshasa_DRC and rural localities in Burkina Faso and Kenya). Statistically significant differences were determined based on non-overlapping confidence intervals between Phase 1 and Phase 2 estimates.

Next, we conducted individual level analysis, focusing on the sample of women completing both surveys. We evaluated changes in exposure to unintended pregnancy risk and changes in contraceptive behaviors (among women at risk at both time points). We used generalized estimation equation models (GEE) to test for differential trends in the use of any contraception between Phase 1 and Phase 2, by women's sociodemographic characteristics (measured at Phase 1) and their experience of economic instability (measured at Phase 2) (Appendix 1). Next, we

For peer review only - http://bmjopen.bmj.com/site/about/guidelines.xhtml

examined the contraceptive method mix of contraceptive adopters and the methods women discontinued between the two surveys. We conducted three multivariable logistic regression models to identify factors related to 1) contraceptive adoption among women who were not using contraception pre-pandemic, 2) contraceptive discontinuation among women who were using a method pre-pandemic, and 3) the experience of an unintended pregnancy during the pandemic.

All analyses applied survey and post-stratification weights to account for the complex survey design, clustering of women within EAs and differential loss-to-follow-up in the case of the longitudinal sample. All analyses were conducted in Stata 16.1 with statistical significance set a priori at $p<0.05$.

RESULTS

The characteristics of pre-pandemic study samples by setting are presented in Table 2. By design, all women in Kinshasa-DRC and Lagos-Nigeria were living in urban areas, while this was the case for 22.8% and 30.2% of the samples in Burkina Faso and Kenya, respectively. The mean age of women ranged from 28.3 years in Kinshasa-DRC to 31.0 years in Lagos-Nigeria. Most women across settings were in union, except in Kinshasa, where this was the case in 42.7% of respondents. Women in Burkina Faso had the highest parity (50.9% with three or more children), followed by Kenya (40.9% with three or more children), while lower parities were observed in Kinshasa and Lagos, where 41.9% and 35.2% of women, respectively, had no children. Educational attainment varied substantially across settings, as 77.6% of women in Burkina Faso had no schooling or a primary level of education, with this proportion dropping to 49.7% in Kenya and to 7.8% and 11.8% in Kinshasa-DRC and Lagos-Nigeria, respectively. Contraceptive practices also varied, with non-use ranging from 52.2% in Kenya to 70.1% in Burkina Faso. Reliance on highly effective methods was greatest in Kenya (21.4%) and Burkina Faso (14.3%), while use of less effective barrier and natural methods was more common in Kinshasa-DRC (32.7%) and Lagos-Nigeria (25.8%).

For peer review only

Table 2. Phase 1 sociodemographic characteristics, by setting

		Burkina Faso (n=6,590)	Kenya (n=9,477)	Kinshasa-DRC (n=2,611)	Lagos-Nigeria (n=1,469)
Age (mean)		28.9	28.8	28.3	31.0
		Column %			
Age	15-19	21.6	21.6	22.2	14.0
	20-24	17.3	17.6	20.9	14.0
	25-29	14.7	16.3	16.3	15.5
	30-34	15.6	15.4	12.3	17.7
	35-39	12.7	11.8	10.7	17.8
	40-44	10.9	9.5	10.4	12.7
	45-49	7.2	7.8	7.2	8.3
Union status	In union†	75.8	59.2	42.7	60.9
	Not in union	24.2	40.8	57.3	39.1
Parity	Nulliparous	24.3	28.3	41.9	35.2
	1-2 children	24.8	30.9	28.1	29.5
	3+ children	50.9	40.9	30.1	35.3
Residence	Urban	22.8	30.2	100.0	100.0
	Rural	77.2	69.8	--	--
Education	No schooling /primary	77.6	49.7	7.8	11.8
	Secondary low	16.4	2.1	72.9	51.2
	Secondary high or more	6.1	48.2	19.3	37.0
Contraception±	None	70.1	52.2	55.4	60.6
	Less effective	5.7	7.2	32.7	25.8
	Effective	9.9	19.2	4.2	6.1
	Highly effective	14.3	21.4	7.8	7.5

†In union defined as married or living with a partner as if married
± Less effective=barrier or natural methods; effective= short-acting hormonal methods, including pills, injectables, patches, or rings; highly effective=IUDs, implants, sterilization

For peer review only

At the population level, 6.2% to 16.8% of women intended a birth in the next 12 months across geographies, with no significant change during the pandemic (Table 3), including in urban and rural areas (Appendix 2). Accounting for past-year sexual activity, fertility intentions, and fecundity, 27.8% to 46.3% of women were exposed to the risk of unintended pregnancy, with the highest proportion observed in Burkina Faso. Exposure to unintended pregnancy risk did not vary over time in any setting.

Contraceptive use (any method) varied widely by setting and was lowest in Burkina Faso and highest in Kenya and Kinshasa province in the DRC. During the pandemic, contraceptive use rose significantly by nine percentage points in Burkina Faso and five percentage points in Kenya. The rise in contraceptive use among women in Burkina Faso was twice as elevated in rural settings (10 percentage points) compared to urban settings (5 percentage points; non-significant difference)

For peer review only - http://bmjopen.bmj.com/site/about/guidelines.xhtml

while increases were comparable and statistically significant in rural and urban Kenya (Appendix 2). Contraceptive use also rose by six percentage points in Lagos-Nigeria (non-significant difference), while it remained stable in Kinshasa-DRC. The proportion of contraceptive users relying on provider-dependent methods ranged from 35.2% in Lagos to 88.3% in Kenya. This proportion remained stable in Kinshasa-DRC and Lagos-Nigeria and declines were non-significant in Burkina Faso and in Kenya overall (overlapping confidence intervals) (Table 3), but dropped by a significant 3 percentage point in rural Kenya (from 92.3 to 89.1%, Appendix 2).

Between 5.1% women in Kenya and 8.5% of women in Burkina Faso were pregnant at the time of the survey with no change over time. Finally, between 1.0% and 2.8% of women across sites had an unintended pregnant at the time of the Phase 2 survey, this proportion remaining stable over time in all sites (Table 3). No significant differences in trends were observed between women in urban and rural settings (Appendix 2).

For peer review only

For peer review only - http://bmjopen.bmj.com/site/about/guidelines.xhtml

1 **Table 3. Cross-sectional reproductive characteristics of women at Phase 1 and Phase 2, by setting**

	Burkina Faso		Kenya		DRC-Kinshasa		Nigeria-Lagos	
	Phase 1 (n=6,590)	Phase 2 (n=6,362)	Phase 1 (n=9,477)	Phase 2 (n=9,310)	Phase 1 (n=2,611)	Phase 2 (n=2,368)	Phase 1 (n=1,469)	Phase 2 (n=1,481)
	% (95% CI)							
Intends to have a child in next year	13.4 (11.8, 15.3)	14.1 (12.3, 16.1)	6.9 (6.1, 7·8)	6.2 (5.5, 6.9)	12.2 (9.3, 15.8)	10.1 (7.8, 13.2)	16.8 (14.6, 19.2)	16.2 (14.6, 18.1)
In need of contraception	44.1 (41.1, 47.2)	44.5 (41.4, 47.4)	46.3 (44.9, 47.6)	45.6 (44.3, 46.9)	27.8 (24.7, 31.1)	29.2 (26.3, 32.2)	39.5 (36.6, 42.5)	40.4 (37.7, 43.2)
Contraceptive use, among women in need	**43.2 (39.0, 47.6)**	**52.3 (48.0, 56.6)**	**71.3 (69.4, 73.2)**	**76.5 (74.6, 78.2)**	71.8 (67.4, 75.9)	72.4 (68.2, 76.2)	63.4 (56.8, 69.5)	69.7 (63.7, 75.2)
Provider-dependent contraception, among contraceptive users	83.4 (79.3, 86.9)	79.1 (74.3, 83.1)	88.3 (86.7, 89.8)	86.3 (84.6, 87.8)	37.3 (29.5, 45.9)	35.6 (28.3, 43.5)	40.1 (33.7, 46.9)	35.2 (29.1, 41.8)
Currently pregnant	8.5 (7.6, 9.6)	7.6 (6.6, 8.4)	5.4 (4.9, 5.9)	5.1 (4.6, 5.7)	5.7 (4.5, 7.1)	5.2 (4.1, 6.5)	4.7 (3.7, 6.0)	4.7 (3.7, 5.9)
Current unintended pregnancy	2.8 (2.1,3.7)	2.1 (1.6,2.7)	2.3 (2.0,2.7)	2.1 (1.7,2.4)	2.6 (1.9,3.6)	2.8 (2.1,3.8)	1.0 (0.6-1.6)	1.0 (0.6.1.7)

2 Numbers in bold indicate statistical significance based the non-overlap of confidence interval

For peer review only

Assessment of individual-level trajectories of women who responded to both surveys indicated that changes in exposure to unintended pregnancy risk among all women ranged from 16.9% in Kinshasa-DRC to 31.5% in Burkina Faso (Figure 1). Patterns of change were similar in rural versus urban settings in Burkina Faso and Kenya (data not shown). Half of changes involved new exposure to risk, while the other half led to cessation of exposure to risk during the pandemic. Among all women, the proportion at risk of unintended pregnancy at both time points (i.e., sustained risk) ranged from 20.5% in Kinshasa-DRC to 42.2% in Kenya. Among women with sustained risk, change in contraceptive status varied from 19.4% in Kenya to 25.2% in Burkina Faso. Contraceptive adoption was more common than discontinuation in Burkina Faso (16.9% versus 8.3%, respectively), Kenya (12.6% versus 6.8%, respectively) and Lagos-Nigeria (15.2% versus 8.7%, respectively) but equivalent in Kinshasa-DRC (11.5% versus 12.6%, respectively). Among women who changed contraceptive status, most adopted or discontinued less effective barrier and natural methods in Kinshasa-DRC (66.9% and 68.5%, respectively) and Lagos-Nigeria (77.8% and 74.1%, respectively), while most adopted or discontinued effective short-acting methods in Burkina Faso (45.1% and 44.7%, respectively) and Kenya (47.8% and 54.2%, respectively). Highly effective long-acting method contributed the least to adoption and discontinuation in Lagos-Nigeria (6.0% and 2.7%, respectively) and accounted for 27.0% of adoption and 39.5% of discontinuation in Burkina Faso. Patterns of change reflect differences in contraceptive method mix among women at risk before COVID-19, as 65.5% and 73.2% of contraceptive users relied on less effective methods in Kinshasa-DRC and Lagos-Nigeria, respectively, versus 15% in Kenya and 19% in Burkina Faso. Conversely, 44.7% of women at risk in Kenya and 47.8% in Burkina Faso relied on highly effective methods pre-pandemic versus 17.4% in Kinshasa-DRC and 19.0% in Lagos-Nigeria.

While results from GEE models identified few statistically significant interactions between time and women's characteristics, signaling limited differences in contraceptive trends by sociodemographic characteristics, some patterns emerged (Table 4). For example, increases in the odds of contraceptive use during COVID-19 relative to the pre-pandemic period were more pronounced among women in their mid-twenties compared to teenagers or women over 40 years in all sites but Kinshasa-DRC. Likewise, increases in the odds of contraceptive use were elevated among parous women but not among nulliparous women in Kenya. In Lagos-Nigeria, increases in the odds of contraceptive use were greater among women with 1-2 children relative to those who had more children. Finally, wealthiest women in Lagos experienced greater increases in the odds of contraceptive use relative to women in the medium wealth category in Lagos. Conversely, there were no differential trends by education, recent loss of income, or recent experience of food insecurity.

For peer review only - http://bmjopen.bmj.com/site/about/guidelines.xhtml

Table 4. Differential trends in contraceptive use by women's sociodemographic characteristics

	Burkina Faso (n=5,207)	Kenya (n=6,932)	DRC-Kinshasa (n=1,967)	Nigeria-Lagos (n=1,088)
	RRR (95% CI)			
Overall	1.4 (1.2, 1.6)	1.3 (1.2, 1.5)	0.9 (0.7, 1.2)	1.3 (1.1, 1.6)
Age#year interaction*	p=0.06	p=0.24	p=0.13	p=0.08
15-19	1.3 (0.8, 2.2)	1.4 (0.7, 2.8)	0.4 (0.1,2.6)	-
20-24	1.4 (1.1, 1.8)	1.3 (1.0, 1.7)	1.5 (0.6,3.6)	1.1 (0.5, 2.2)
25-29	1.7 (1.2, 2.3)	1.5 (1.2, 1.9)	1.2 (0.7,2.2)	2.9 (1.5, 5.6)
30-34	1.2 (0.8, 1.6)	1.4 (1.2, 1.6)	0.4 (0.2,0.7)	1.7 (1.0, 3.1)
35-39	1.6 (1.2, 2.3)	1.5 (1.2, 1.8)	1.1 (0.6,1.8)	1.0 (0.7, 1.4)
40-44	1.5 (1.0, 2.1	1.1 (0.9, 1.3)	0.9 (0.6,1.4)	1.2 (0.7, 2.1)
45-49	0.8 (0.7, 1.1)	1.1 (0.9, 1.4)	1.0 (0.6,1.7)	1.1 (0.6, 2.0)
Parity#year interaction*	p=0.34	p=0.08	p=0.88	p=0.004
Nulliparous	-	0.7 (0.2, 2.1)	3.3 (0.2, 55.9)	-
1-2	1.4 (1.1, 1.8)	1.5 (1.2, 1.8)	0.8 (0.5, 1.3)	2.1 (1.4, 23.0)
3+	1.4 (1.2, 1.6)	1.3 (1.1, 1.4)	1.0 (0.7, 1.2)	1.1 (0.8, 1.4)
Residence#year interaction*	p=0.32	p=0.54		
Urban	1.3 (1.1, 1.4)	1.2 (1.0, 1.5)	--	--
Rural	1.4 (1.2, 1.6)	1.3 (1.2, 1.5)	--	--
Wealth#year interaction*	p=0.57	p=0.61	p=0.42	p=0.08
Poorest	1.5 (1.2, 2.0)	1.4 (1.2, 1.6)	0.9 (0.5, 1.4)	1.5 (0.9, 2.3)
Medium	1.3 (1.0, 1.6)	1.3 (1.1, 1.6)	1.1 (0.7, 1.8)	1.1 (0.7, 1.6)
Richest	1.3 (0.9, 1.3)	1.2 (0.9, 1.5)	1.1 (0.7, 1.8)	2.0 (1.3, 2.9)
Loss of income last 12 months	p=0.30	p=0.48	p=0.71	p=0.09
None	-	1.2 (1.0, 1.4)	-	-
Partial	1.4 (1.2, 1.6)	1.4 (1.2, 1.5)	0.9 (0.7,1.3)	1.4 (1.1, 1.9)
Complete	1.5 (1.0, 2.3)	1.3 (1.1, 1.5)	0.9 (0.7, 1.2)	1.2 (0.8, 1.7)
Food insecurity	p=0.24	p=0.33	p=0.85	p=0.10
No	1.4 (1.2, 1.5)	1.3 (1.2, 1.4)	1.0 (0.7, 1.3)	1.4 (1.1, 1.7)
Yes	1.8 (1.1, 2.9)	1.5 (1.2, 1.9)	0.7(0.5, 1.1)	0.8 (0.6, 1.1)
Education	p=0.29	p=0.87	p=0.26	p=0.22
No schooling/primary	1.4 (1.2, 1.6)	1.3 (1.2, 1.4)	0.6 (0.3, 1.3)	1.2 (0.7, 2.0)
Secondary low	1.3 (0.8, 2.0)	1.2 (0.6, 2.4)	1.0 (0.8, 1.3)	1.2 (0.9, 1.7)
Secondary high or higher	0.7 (0.3,1.3)	1.3 (1.1,1.6)	0.7 (0.4,1.2)	1.9 (1.4,2.6)

*p-values refer to the significance of the interaction term between all categories of a demographic factor and the outcome of interest (contraceptive use)

Few factors were related to contraceptive adoption among women who were not using a method pre-pandemic and to contraceptive discontinuation among those using contraception pre-pandemic (Appendix 3). The odds of adopting contraception were higher among women with secondary education or higher in Burkina Faso and Kenya, relative to less educated women, and among women in the richest tertile, relative to the poorest women in Kenya. Conversely the oldest women (35-49 years) were less likely to adopt a method in Kinshasa-DRC and Kenya and, more likely to discontinue their method in Kenya. Discontinuation was also more common among parous women compared to nulliparous women in Kenya. Finally, women with lower secondary education in Kinshasa were less likely to discontinue their method than women with no schooling or primary education.

For peer review only - http://bmjopen.bmj.com/site/about/guidelines.xhtml

Finally, factors associated with characterizing an unintended pregnancy during COVID-19 among women who had sexual intercourse in the last 12 months are reported in Appendix 4. Older women (35 years or older) and women using long-acting contraception were less likely to experience an unintended pregnancy across sites. Women with higher education and from wealthier households were less likely to experience an unintended pregnancy in Kenya, while women of higher parity were more likely to experience unintended pregnancies in Burkina Faso and Kinshasa-DRC. COVID-19-related economic vulnerability was unrelated to unintended pregnancy across sites.

DISCUSSION

This study confirms women's resiliency in contraceptive use to prevent unintended pregnancies across a diversity of sub-Saharan contexts, despite the profound economic and social disruptions caused by the COVID-19 pandemic [2-4,16-17]. While we found a sustained need for contraception, we also observed an increase in contraceptive coverage in three of the four geographies studied. Within Burkina Faso and Kenya, increases in contraceptive protection corresponded with a decrease in the use of methods requiring a prescription, which was only significant in rural Kenya, but most women still used effective or highly effective methods, including women who adopted methods during the pandemic. These contraceptive dynamics coincide with the absence of significant changes in pregnancy rates or unintended pregnancy rates across sites.

These results challenge initial forecasts of rising unintended pregnancies due to service disruptions to sexual and reproductive health services in LMICs [7]. The absence of negative population-level effects of the pandemic on contraceptive behaviors and reproductive outcomes, noted in a previous study focusing on the early months of the pandemic [14,15], and confirmed in a study using one year follow data [22], are surprisingly reassuring and suggest several potential explanations [24].

First, while disruptions to sexual and reproductive health (SRH) services were reported in the early stage of the pandemic [5,24-29], patterns of service disruptions varied significantly by level, duration, type of service and geography [25-27]. Discontinuity of services was often short-lived—several studies reporting rapid post-lockdown rebounds in service provision [24,28-30].

Further, it is possible that the quality of facility-based registers, including clients' family planning records, were impacted due to understaffing amid COVID-19-related disruptions and, thus, early pandemic provision of care was underreported [31].

At the same time, the expansion or continued use of high impact practices in family planning [32], such as community health workers, mobile outreach, supply chain management, and digital health initiatives, may also have compensated for facility-based disruptions to care. For example, the expansion of community health workers to deliver family planning services resulted in a 2.5 percent increase in the number of family planning clients in rural Kenya in the summer of 2020 [33]. These initiatives were encouraged by the Kenyan government who issued guidelines to support "Continuity of Reproductive, Maternal, Newborn and Family Planning Care and Services in the Background of COVID-19 Pandemic" in the month following the first reported case of COVID- 19 [34]. More generally, a recent WHO report of SRH services in Africa during COVID-19 indicates that 15 out the 17 countries reflected in the survey had integrated SRH services in

For peer review only - http://bmjopen.bmj.com/site/about/guidelines.xhtml

their continuity of care plan during COVID-19, including awareness campaigns and communication messages about family planning [26].

A third explanation lies in the resilience of women in intensifying their efforts or finding alternative solutions to access contraception and self-manage their contraceptive needs [25]. This hypothesis is plausible given that our initial investigation into family planning amid COVID-19, conducted between May and July of 2020, found that only 3.8-14.4% of women at risk of unintended pregnancy were not using contraception for COVID-19-related reasons in Burkina Faso and Kenya; these women primarily cited fear of COVID-19 transmission [15]. Approximately six months later, the data collected via PMA surveys between November 2020 and January 2021 included in the present analysis reveal 9 to 35% reported difficulties accessing any health service in the last year, however 89-93% of these were ultimately successful in obtaining services (www.pmadata.org). Self-provision of oral contraceptive pills during the COVID-19 is likely to have facilitated women's access to short-acting contraception [24], while the impact of DMPA-SC self-administration policies should be further investigated to evaluate their contribution to continuity of family planning use during the pandemic.

These overall trends remain encouraging, but a critical concern over widening social disparities in family planning access may still arise in the face of increasing economic hardships [3-4]. Our findings provide no evidence of adverse SRH outcomes among women who report food insecurity, or those who experienced substantial loss of income since the start of the COVID-19 pandemic. Recent FP2030 policy commitments towards family planning (https://fp2030.org/FP2030-commitment-makers), including increased public funding, and expansion of free access to contraception in some countries, such as Burkina Faso in 2019 are likely to have mitigated the economic impact of COVID-19, but the affordability of contraception remains a prominent concern for women experiencing economic hardship [18].

Our results should be considered with several limitations in mind. Specifically, the loss to follow-up rate ranged from 21-27% across geographies, which is likely to affect the reported estimates of change. However, the use of replacement households in Phase 2 of data collection was designed to minimize impacts to population-level estimates. Further, post-stratification weights were used in panel analysis to limit the potential for attrition biases; stable estimates of sociodemographic distributions across samples were observed (Appendix 5). A second limitation is our measure of change using two time points, which prevents a more comprehensive assessment of changes over the full time-period, including potential gaps in contraceptive use. A further evaluation of contraceptive use dynamics, using calendar data, was beyond the scope of this analysis but could provide a more thorough investigation of contraceptive gaps, albeit limited by the quality of the calendar data [35]. Our data in DRC and Nigeria are not nationally representative but rather reflect urban trends. Stratified analyses comparing urban and rural Burkina Faso and Kenya found no major differences in family planning trends, however, these results cannot be generalized to DRC and Nigeria, given distinct patterns of SRH disruptions reported in several studies in Africa [26-27]. Finally, our sample sizes were limited to detect significant trends in rare events, such as pregnancy outcomes, or differential trends by women's sociodemographic and economic background. In addition, a more thorough investigation of changing fertility intentions and their impact on subsequent reproductive outcomes over the course of the pandemic could shed more light on the ways fertility responds to external shocks.

For peer review only - http://bmjopen.bmj.com/site/about/guidelines.xhtml

Our study also has several strengths. First, our population-based approach provides a complimentary perspective to data collected via Health Management Information Systems (HMIS), showing little change in behaviors and SRH outcomes, despite facility-based disruptions, suggesting the importance of non-facility-based services and self-care practices in ensuring continuity of family planning care [26]. Second, our novel study design, integrating perspectives from representative cross-sectional data and panel data, offers the double advantage of examining population and individual trends between pre- COVID-19 and COVID-19 periods, with the potential of identifying disparities in COVID-19 effects on family planning practices. The use of a comprehensive set of indicators ranging from pregnancy exposure to contraceptive practices and reproductive outcomes also provides a more holistic understanding of fertility processes during a pandemic, elucidating how family planning needs and use evolve concurrently. In particular, our focus on unintended pregnancy provides added information about women's ability to achieve their reproductive goals in the context of the global pandemic. Finally, the cross-national/regional nature of the study, enhanced by using a common study protocol and indicators across geographies strengthens comparative trends analysis, supporting previously reported variations in COVID-19 effects on SRH services [26-27].

In conclusion, the current study provides important insights on the resilience of women across geographically and socially diverse contexts in sub-Saharan Africa in sustaining contraceptive practices amid the COVID-19 pandemic. Renewed national and international commitments to family planning made as part of the 2011 Ouagadougou Partnership [36] and 2012 London Summit on Family Planning [37] have likely contributed to the resiliency and innovations in family planning delivery systems. The integration of maternal health and other reproductive health services into continuity of care plans during COVID-19 is a reflection of such commitments [26]. However, investments in these systems must be sustained to ultimately reduce unsatisfied demand for contraception and achieve universal access to SRH services. With rising global poverty and the diversion of international family planning funding (as exemplified by the recent shortfall in UNFPA's funding for family planning programs), there is continued cause for concern over women's access to affordable contraceptive services in-sub-Saharan Africa. Sustained global and local advocacy and monitoring are needed to ensure accountability and progress towards essential SRH service provision, including women's access to and use of contraception.

Figure 1: Changes in individual level risk of unintended pregnancy, and use of contraception between Phases 1 and 2, and contraceptive method mix between adopters and discontinuers of contraception

Contributorship: FMO, PA, GG, PG, LZ and PAA designed the PMA panel study, CM, FMO, PA, GG, PG, LZ and PAA designed the COVID module, CM, CK, SW designed this particular study. CM conducted the analysis, CM and KW conducted the literature review, CM, KW, CK, SW wrote the initial draft, and FMO, PA, GG, PG, LZ, PAA interpreted the results and revised the manuscript.

Funding statement: This research was developed under grants OPP1198333 and OPP1198339 awarded by the Bill & Melinda Gates Foundation to Johns Hopkins Bloomberg School of Public Health and Jhpiego.

Competing Interests: None declared.

For peer review only - http://bmjopen.bmj.com/site/about/guidelines.xhtml

Ethical Approval
PMA surveys received approval from ethical committees in each geography (Burkina Faso: Comité d'Ethique Institutionnel Pour La Recherche en Santé, Ministère de l'Enseignement Supérieur, de la Recherche Scientifique et de l'Innovation; Democratic Republic of the Congo: University of Kinshasa School of Public Health; Kenya: Kenyatta National Hospital—University of Nairobi Ethics Research Committee; Nigeria: Lagos State University Teaching Hospital Health Research Ethics Committee). PMA also received approval from the Institutional review board of the Johns Hopkins School of Public Health (IRB00014702). Public health preventive measures (i.e., social distancing, mask-wearing, use of hand sanitizer) were instituted in Phase 2 to prevent COVID-19 transmission during data collection.

Data Availability Statement: Data are publicly available at pmadata.org.

REFERENCES

1 Our world data. Cumulative confoirmed covid-19 deaths by world reigion. https://ourworldindata.org/grapher/cumulative-covid-deaths-region
2 Congressional research Service. Global Economic Effects of COVID-19. Nov 2010. https://sgp.fas.org/crs/row/R46270.pdf
3 Selassie AA, Hakobyan S. six-charts-show-the-challenges-faced-by-sub-saharan-africa, IMF African Department, April 15, 2021 https://www.imf.org/en/News/Articles/2021/04/12/na041521-six-charts-show-the challenges-faced-by-sub-saharan-africa
4 Gummerson E, Cardona C, Anglewicz P, *et al.* The wealth gradient and the effect of COVID-19 restrictions on income loss, food insecurity and health care access in four sub-Saharan African geographies. *PLoS One* 2021;**16**:e0260823. doi:10.1371/journal.pone.0260823
5. International Planned Parenthood Federation (IPPF). Contraception and COVID-19: Disrupted supply and access. . https://www.ippf.org/blogs/contraception-and-covid-19-disrupted-supply-and-access. 2020
6. Endler M, Al-Haidari T, Benedetto C, Chowdhury S, Christilaw J, El Kak F, et al. How the coronavirus disease 2019 pandemic is impacting sexual and reproductive health and rights and response: results from a global survey of providers, researchers, and policy-makers. Acta Obstet Gynecol Scand. 2021;100(4):571–578.
7 Riley T, Sully E, Ahmed Z, *et al.* Estimates of the Potential Impact of the COVID-19 Pandemic on Sexual and Reproductive Health In Low- and Middle-Income Countries. *Int Perspect Sex Reprod Health* 2020;**46**:73–6.
8 UNFPA. Impact of the COVID-19 Pandemic on Family Planning and Ending Gender-based Violence, Female Genital Mutilation and Child Marriage. 2020. https://www.unfpa.org/sites/default/files/resource-pdf/COVID-19_impact_brief_for_UNFPA_24_April_2020_1.pdf
9 Hall KS, Samari G, Garbers S, *et al.* Centring sexual and reproductive health and justice in the global COVID-19 response. *Lancet* 2020;**395**:1175–7. doi:10.1016/S0140-6736(20)30801-1
10 Govender D, Naidoo S, Taylor M. Don't Let Sexual and Reproductive Health become Collateral Damage in the Face of the COVID-19 Pandemic: A Public Health Perspective.

For peer review only - http://bmjopen.bmj.com/site/about/guidelines.xhtml

Afr J Reprod Health. 2020 Jun;24(s1):56-635
11 Hussain R et al., Unmet Need for Contraception in Developing Countries: Examining Women's Reasons for Not Using a Method, New York: Guttmacher Institute, 2016, http://www.guttmacher.org/report/unmet-need-for-contraception-in-develop....
12 Bearak JM, Popinchalk A, Beavin C, *et al*. Country-specific estimates of unintended pregnancy and abortion incidence: a global comparative analysis of levels in 2015–2019. *BMJ Global Health* 2022;**7:**e007151
13 United Nations Department of Economic and Social Affairs Population facts. July 2019. Global progress in satisfying the need for family planning https://www.un.org/en/development/desa/population/publications/pdf/popfacts/PopFacts_2019-3.pdf, Accessed 10th Mar 2021
14 Wood SN, Karp C, OlaOlorun F, *et al.* Trends in the need for and use of contraception before and during COVID-19 in four sub-Saharan African geographies: results from population-based national or regional cohort surveys. *Lancet Glob Heal* 2021;**9**:e793-801.
15 Karp C, Wood SN, Guiella G, *et al.* Contraceptive dynamics during COVID-19 in sub--Saharan Africa: longitudinal evidence from Burkina Faso and Kenya. *BMJ Sex Reprod Heal* 2021;**0**:1–9. doi:10.1136/bmjsrh-2020-200944
16 Mahler DG, Yonzan N, Lakner C, *et al.* Updated estimates of the impact of COVID-19 on global poverty: Turning the corner on the pandemic in 2021? World Bank Data Blog. 2021;:1–10.https://blogs.worldbank.org/opendata/updated-estimates-impact-covid-19-global-poverty-turning-corner-pandemic-2021
17 Our World in Data. Policy Responses to the Coronavirus Pandemic--Statistics and Research. https://ourworldindata.org/policy-responses-covid (accessed 26 Jan 2022).
18 Decker MR, Wood SN, Thiongo M, *et al.* Gendered health, economic, social and safety impact of COVID-19 on adolescents and young adults in Nairobi, Kenya. *PLoS One* 2021;**16**:e0259583.
19 Karp C, Moreau C, Sheehy G, *et al.* Youth Relationships in the Era of COVID-19: A Mixed-Methods Study Among Adolescent Girls and Young Women in Kenya. *J Adolesc Heal* 2021;**69**:754–61. doi:10.1016/j.jadohealth.2021.07.017
20 Adu, P.A., Stallwood, L., Adebola, S.O. *et al.* The direct and indirect impact of COVID-19 pandemic on maternal and child health services in Africa: a scoping review. *glob health res policy* **7,** 20 (2022). https://doi.org/10.1186/s41256-022-00257-z
21 Greenleaf AR, Gadiaga A, Guiella G, *et al.* Comparability of modern contraceptive use estimates between a face-to-face survey and a cellphone survey among women in Burkina Faso. *PLoS One* 2020;**15**:1–15. doi:10.1371/journal.pone.0231819
22 Backhaus, A. Pregnancies and contraceptive use in four African countries during the COVID-19 pandemic. Vienna Yearbook of Population Research 2022;20. doi:10.1553/populationyearbook2022.dat.4
[Dataset] 23 Performance Monitoring for Action (PMA), Institut national de la statistique et de la demographie (Burkina Faso), Kinshasa School of Public Health (Democratic Republic of the Congo) Centre for Research, Evaluation Resources and Development, Bayero University Kano (Nigeria), International Centre for Reproductive Health (Kenya) 2019-2021. Baltimore, MD: PMA2020, Bill & Melinda Gates Institute for Population and Reproductive Health, Johns Hopkins Bloomberg School of Public Health.

For peer review only - http://bmjopen.bmj.com/site/about/guidelines.xhtml

24 Thomas AB, Yaya S. Looking ahead in the COVID - 19 pandemic : emerging lessons learned for sexual and reproductive health services in low- and middle- income countries. *Reprod Health* 2021;**4**:1–8.
25 Assefa N, Sié A, Wang D, Korte ML, Hemler EC, Abdullahi YY, et al. Reported barriers to healthcare access and service disruptions caused by COVID-19 in Burkina Faso, Ethiopia, and Nigeria: a telephone survey. Am J Trop Med Hygiene. 2021;1(aop).
26 Otieno, George & Ouedraogo, Leopold & Nkurunziza, Triphonie & Asmani, Chilanga & Elamin, Hayfa & Muriithi, Assumpta & Onyiah, Amaka & Conombo, Ghislaine & Kidula, Nancy & Tall, Fatim & Sekpon, Ameyo & Adegboyega, Ayotunde. (2021). Continuity of Essential Sexual and Reproductive Health Services During Covid-19 Pandemic in the Who African Region. 10.21203/rs.3.rs-316744/v1.
27 APHRC, 2022. *Impact of the COVID-19 Pandemic on Sexual and Reproductive Health Services in Burkina Faso, Ethiopia,*, Africa Population Health Research Center. Retrieved from https://policycommons.net/artifacts/2460145/impact-of-the-covid-19-pandemic-on-sexual-and-reproductive-health-services-in-burkina-faso-ethiopia/3481942/ on 28 Jul 2022. CID: 20.500.12592/qgq1pf.
28 Fuseini K, Jarvis L, Ankomah A, Bintou Mbow F, Hindin MJ. Did COVID-19 Impact Contraceptive Uptake? Evidence from Senegal. Stud Fam Plann. 2022 Jun;53(2):301-314. doi: 10.1111/sifp.12195. Epub 2022 Apr 18. PMID: 35436350; PMCID: PMC9115390.
29 Leight J, Hensly C, Chissano M, *et al.* Short-term effects of the COVID-19 state of emergency on contraceptive access and utilization in Mozambique. *PLoS One* 2021;**16**:1–16. doi:10.1371/journal.pone.0249195
30 Ameh C, Banke-Thomas A, Balogun M, *et al.* Reproductive maternal and newborn health Providers' assessment of facility preparedness and its determinants during the COVID-19 pandemic in Lagos, Nigeria. *Am J Trop Med Hyg* 2021;**104**:1495–506. doi:10.4269/ajtmh.20-1324
31 Karp C, Moreau C, Olaolorun F, *et al.* Family planning service disruptions amid COVID-19: Longitudinal evidence from facilities in seven low- and middle-income countries Authors. 2021. https://ipc2021.popconf.org/uploads/211072
32 Mickler AK, Carrasco MA, Raney L, *et al.* Applications of the High Impact Practices in Family Planning during COVID-19. *Sex Reprod Heal Matters* 2021;**29**. doi:10.1080/26410397.2021.1881210
33 PSI. Serving Sara during the COVID-19 pandemic: Lessons learned in family planning adapatations. 2021;:1–13.https://www.psi.org/wp-content/uploads/2021/08/PSI_COVID_Briefs_Final_29July2021.pdf
34 Government of Kenya M of H. Kenya COVID19 RMNH Guidelines: A Kenya Practical Guide for Continuity of Reproductive, Maternal, Newborn and Family Planning Care and Services in the Background of COVID19 Pandemic. 2020. https://kenya.unfpa.org/sites/default/files/pub-pdf/KENYA - COVID19 RMNH GUIDELINES.pdf
35 The Ouagadougou Partnership. 2021.https://partenariatouaga.org/en/about-us/the-partnership/ (accessed 27 Jan 2022).
36 Bradley SE., Sarnak D, Breckler E, *et al.* Measuring Dynamic Contraceptive Use: The Quality and Consistency of DHS Calendar Data. 2021.
37 The London Summit on Family Planning. 2012.

For peer review only

Figure 1: Changes in individual level risk of unintended pregnancy, and use of contraception between Phases 1 and 2, and contraceptive method mix between adopters and discontinuers of contraception

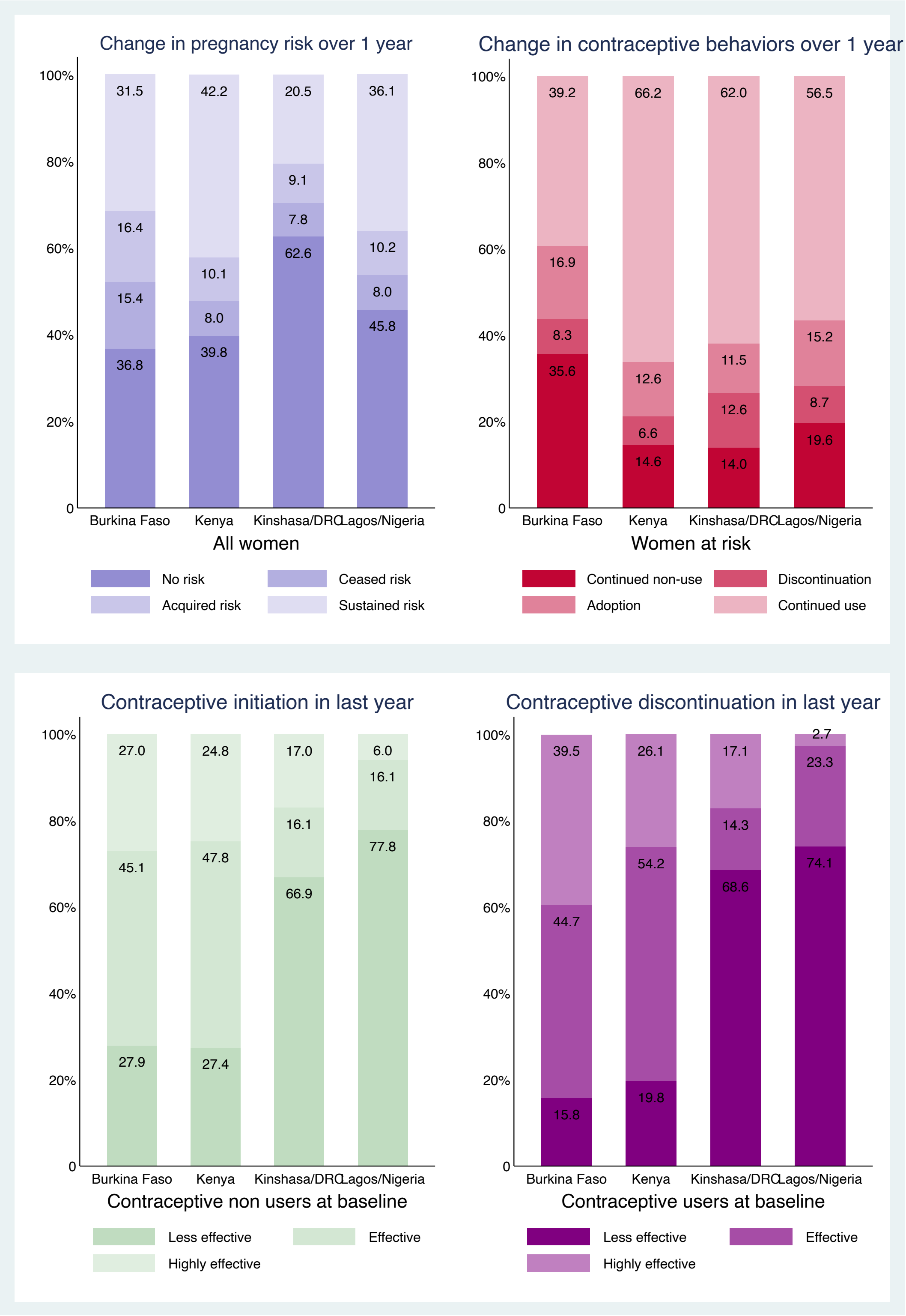

APPENDIX 1

The following GEE model was used to test for differential trends in the use of any contraception between Phase 1 and Phase 2, by women's sociodemographic characteristics

$\text{Logit}\{E(y_{it})\} = \beta_n x_{nit} * t$, $y \sim \text{Bernoulli}$

Y_{it} represents the response for each subject *i*, measured at different time points (*t*=1,2).
$Xn=(X_1, X_2, X_3,\ldots,Xn$ represent sociodemographic variables
x_n represents each sociodemographic variable
βn represents the slope of the outcome for variable x_n

We used a logit function as the outcome *y* (contraception use) is binary with a Bernoulli distribution. We used unstructured-free estimation to account for within-subject correlation

For peer review only

APPENDIX 2 Cross-sectional reproductive characteristics of women at Phase 1 and Phase 2, by urban and rural setting

	Urban Burkina Faso		Rural Burkina Faso		Urban Kenya		Rural Kenya		DRC-Kinshasa		Nigeria-Lagos	
	Phase 1 (n=)	Phase 2	Phase 1 (n=)	Phase 2	Phase 1 (n=)	Phase 2	Phase 1 (n=)	Phase 2	Phase 1 (n=2,611)	Phase 2	Phase 1 (n=1,469)	Phase 2
Intends to have a child in next year	18.6 (16.9, 20.6)	18.2 (16.8, 19.7)	11.9 (9.9, 14.3)	12.9 (10.8, 15.5)	8.4 (6.9, 10.1)	7.8 (6.5, 9.4)	6.3 (5.4, 7.4)	5.4 (4.7, 6.3)	12.2 (9.3, 15.8)	10.1 (7.8, 13.2)	16.8 (14.6, 19.2)	16.2 (14.6, 18.1)
In need of contraception	33.8 (31.9, 35.8)	35.5 (33.4, 37.7)	47.2 (43.4, 51.0)	47.1 (43.2, 51.0)	43.2 (40.9, 45.5)	43.7 (41.5, 45.9)	47.6 (46.0, 49.2)	46.4 (44.8, 48.1)	27.8 (24.7, 31.1)	29.2 (26.3, 32.2)	39.5 (36.6, 42.5)	40.4 (37.7, 43.2)
Contraceptive use, among women in need	61.5 (58.0, 64.9)	65.9 (62.8, 68.9)	39.4 (34.5, 44.5)	49.4 (44.3, 54.5)	79.1 (75.8, 82.0)	82.6 (79.8, 85.1)	68.2 (65.9, 70.5)	73.9 (71.6, 76.2)	71.8 (67.4, 75.9)	72.4 (68.2, 76.2)	63.4 (56.8, 69.5)	69.7 (63.7, 75.2)
Provider-dependent contraception, among contraceptive users	73.5 (69.5, 77.0)	68.7 (64.5, 72.7)	86.7 (81.3, 90.8)	82.1 (75.9, 87.0)	79.7 (75.8, 83.0)	80.1 (76.4, 83.3)	92.3 (90.8, 93.6)	89.1 (87.4, 90.6)	37.3 (29.5, 45.9)	35.6 (28.3, 43.5)	40.1 (33.7, 46.9)	35.2 (29.1, 41.8)
Currently pregnant	6 (5.2, 6.9)	6.1 (5.3, 7.0)	9.3 (8.1, 10.6)	8.1 (6.7, 9.6)	6.1 (5.1, 7.2)	4.2 (3.5, 5.1)	5.1 (4.5, 5.7)	5.5 (4.9, 6.2)	5.7 (4.5, 7.1)	5.2 (4.1, 6.5)	4.7 (3.7, 6.0)	4.7 (3.7, 5.9)
Current unintended pregnancy	1.6 (1.2,2.1)	1.2 (1.0,1.6)	3.1 (2.3,4.2)	2.3 (1.7,3.2)	2.2 (1.7,2.8)	1.5 (1.0,2.1)	2.3 (1.9,2.8)	2.3 (1.9,2.8)	2.6 (1.9,3.6)	2.8 (2.1,3.8)	1 (0.6-1.6)	1 (0.6.1.7)

For peer review only - http://bmjopen.bmj.com/site/about/guidelines.xhtml

APPENDIX 3. Sociodemographic factors related to contraceptive adoption and discontinuation between Phases 1 and 2--results of multivariable logistic regression models, per setting

	Burkina Faso		Kenya		Kinshasa-DRC		Lagos-Nigeria	
	Adoption (n=972)	Discontinuation. (n=917)	Adoption (n=929)	Discontinuation. (n=2183)	Adoption (n=126)	Discontinuation. (n=321)	Adoption (n=156)	Discontinuation . (n=271)
	aOR (95% CI)							
Age								
15-24	ref	ref	ref	ref	ref	ref	ref	ref
25-34	1.3 (0.7-2.5)	**3.7 (1.1,12.0)**	0.7 (0.4-1.3)	1.2 (0.6-2.32)	0.5 (0.1-2.7)	2.1 (0.5-9.0)	-	1.0 (0.2-6.6)
35-49	1.0 (0.4-2.3)	2.9 (0.8-11.1	**0.4 (0.2-0.8)**	**2.0 (1.1-3.9)**	**0.1 (0.0-0.8)**	0.9 (0.2-4.7)	3.9 (0.3-51.3)	3.0 (0.4-24.7
Parity								
0-1	ref	ref	ref	ref	ref	ref	ref	ref
2-3	2.0 (1.0-3.9)	0.4 (0.1-1.6)	1.9 (1.0-3.8)	**0.4 (0.3-0.8)**	2.1 (0.10.4)	0.4 (0.2-1.6)	0.3 (0.0-2.3)	0.5 (0.1-1.9)
4+	1.6 (0.6-4.8)	0.9 (0.3-3.3)	1.4 (0.7-2.9)	**0.5 (0.2-0.9)**	1.3 (0.2-7.1)	0.6 (0.2-1.6)	0.6 (0.4-6.1)	0.3 (0.1-1.)
Residence								
Urban	1.3 (0.7-2.3)	0.8 (0.3-2.-2)	1.1 (0.7-1.6)	0.8 (0.5-1.3)	--	--	--	--
Rural	ref	ref	ref	ref	--	--	--	
Education								--
No schooling /primary	ref	ref	ref	ref	ref	ref	ref	ref
Secondary low	**6.0 (2.8-12.8)**	1.6 (0.6-4.5)	1.1 (0.5-2.8)	0.6 (0.1-2.2)	1.3 (0.2-7.1)	**0.4 (0.2-1.0)**	1.6 (0.4-6.1)	0.6 (0.2-2.0)
Secondary high	**22.6 (4.4-116.7)**	0.9 (0.3-3.3)	**2.0 (1.3-3.0)**	1.2 (0.8-1.7)	0.8 (0.1-7.5)	0.6 (0.2-2.7)	3.5 (0.8-15.4)	0.4 (0.1-2.0)
Food insecurity								
Yes	0.8 (0.3-2.3)	1.1 (0.3,3.6)	0.9 (0.6-1.5)	1.2 (0.6-2.1)	0.3 (0.1-1.3)	0.7 (0.3-1.5)	**-**	0.4 (0.0-3.8)
No	ref	ref	ref	ref	ref	ref	ref	ref
Wealth								
Poorest	ref	ref	ref	ref	ref	ref	ref	ref
Medium	0.7 (0.4-1.2)	0.7 (0.4,1.3)	1.4 (0.9-2.0)	1.0 (0.6-1.5)	0.8 (0.2-2.9)	0.9 (0.4-2.1)	0.7 (0.2-2.46	0.8 (0.3-2.2)
Richest	0.8 (0.4-1.5)	0.5 (0.2-1.6)	**2.0 (1.2-3.4)**	1.0 (0.6-1.7)	0.9 (0.2-3.1)	0.6 (0.2-1.4)	1.2 (0.4-3.9)	0.6 (0.2-2.1)

For peer review only

APPENDIX 4. Sociodemographic factors related to having an unintended pregnancy in Phase 2--results of multivariable logistic regression models, per setting

	Burkina Faso N=4018	Kenya N=5616	Kinshasa-DRC N=1489	Lagos-Nigeria N=836
Age				
15-24	ref	ref	ref	ref
25-34	**0.2 (0.1-0.5)**	0.7 (0.5-1.2)	0.4 (0.2-1.1)	0.3 (0.1,1.0)
35-49	**0.1 (0.0-0.4)**	**0.1 (0.1-0.3)**	**0.1 (0.0-0.3)**	**0.1 (0.0-0.3)**
Marital status				
Not in a cohabitating union	ref	ref	ref	ref
Married or in union	1.1 (0.4-3.1)	0.7 (0.45-1.2)	**2.4 (1.1-5.1)**	2.9 (0.4-21.6)
Parity				
0-1	ref	ref	ref	ref
2-3	1.6 (0.7-3.7)	1.2 (0.7-2.2)	**3.6 (1.4-8.9)**	1.3 (0.1-10.9)
4+	**5.1 (1.3-19.9)**	1.4 (0.6-3.0)	**3.7 (1.2-11.4)**	0.8 (0.0-21.2)
Residence				
Urban	0.9 (0.4-1.9)	0.9 (0.4-1.6)	--	--
Rural	ref	ref	--	--
Education				
No schooling /primary	ref	ref	ref	ref
Secondary low	1.6 (0.8-3.2)	0.4 (0.1-1.6)	0.5 (0.2-1.3)	0.6 (0.1-4.3)
Secondary high	0.6 (0.2-2.0)	**0.4 (0.2-0.7)**	0.6 (0.1-3.2)	0.3 (0.0-3.5)
Food insecurity				
Yes	1.6 (0.7-3.8)	1.3 (0.7-2.2)	0.9 (0.6-2.0)	**-**
No	ref	ref	ref	
Wealth				
Poorest	ref	ref	ref	ref
Medium	1.4 (0.8-2.7)	**0.6 (0.3-0.9)**	**0.4 (0.2-1.0)**	0.3 (0.0-2.1)
Richest	0.8 (0.3-2.0)	**0.4 (0.2-0.8)**	0.5 (0.2-1.2)	1.1 (0.2-5.1)
Contraception at phase 1				
None	ref	ref	ref	ref
Less effective (barrier/natural)	1.0 (0.3-2.8)	1.4 (0.8-2.7)	1.1 (0.6,5.1)	0.3 (0.0,1.4)
Effective (short-acting hormonal)	1.4 (0.7-2.9)	1.1 (0.6-1.9)	**1.8 (0.2-0.8)**	0.4 (0.0-5.3)
Highly effective (long-acting methods)	**0.1 (0.0-0.2)**	**0.4 (0.2-0.8)**	**0.1 (0.0-0.3)**	**0.1 (0.0-0.8)**

± Less effective=barrier or natural methods; effective= short-acting hormonal methods, including pills, injectables, patches, or rings; highly effective=IUDs, implants, sterilization

For peer review only

APPENDIX 5. Sociodemographic characteristics by sample and setting, weighted

	Burkina Faso			Kenya			Kinshasa-DRC			Lagos-Nigeria		
Overall	Phase 1[a]	Phase 2	Panel	Phase 1	Phase 2	Panel	Phase 1	Phase 2	Panel	Phase 1	Phase 2	Panel
Age												
15-19	21.6	20.9	20.9	21.6	22.3	21.9	22.2	23.2	22.3	14.0	15.8	13.4
20-24	17.3	18.4	17.4	17.6	17.4	17.7	20.9	20.4	21.4	14.0	12.6	12.8
25-29	14.7	15.0	14.8	16.3	15.5	16.3	16.3	15.2	16.0	15.5	16.0	15.7
30-34	15.6	15.2	16.1	15.4	15.6	15.1	12.3	13.4	12.3	17.7	16.1	18.1
35-39	12.7	12.9	12.6	11.8	11.6	11.8	10.7	10.8	10.7	17.8	16.8	18.4
40-44	10.9	10.9	11.1	9.5	9.6	9.4	10.4	10.2	10.2	12.7	12.8	13.0
45-49	7.2	6.9	7.1	7.8	8.1	7.9	7.2	6.8	7.2	8.3	10.0	8.5
Parity												
Nulliparous	24.3	22.7	23.2	28.3	29.2	28.5	41.9	41.4	41.7	35.2	36.5	32.9
1-2	24.8	24.9	24.9	30.9	30.1	29.9	28.1	28.7	28.7	29.5	28.8	30.4
3+	50.9	52.5	51.9	40.9	40.7	41.6	30.1	30.0	29.7	35.3	34.7	36.7
Residence												
Urban	22.8	22.3	22.9	30.2	30.4	30.5	100.0	100.0	100.0	100.0	100.0	100.0
Rural	77.2	77.7	77.1	69.8	69.6	69.5	--	0.0	0.0	0.0	0.0	0.0
Wealth Tertile												
Poorest	330	33.5	33.1	35.9	34.6	35.8	33.9	31.7	33.6	34.2	33.0	34.7
Middle	33.5	32.9	33.2	33.5	34.2	33.6	32.3	33.0	32.7	30.7	33.0	30.8
Richest	33.5	33.7	33.7	30.6	31.3	30.6	33.8	35.4	33.8	35.1	34.0	34.6
Loss of income last 12 months												
None		0.0	0.0		22.4	21.1		0.0	0.0		0.0	0.0
Complete		9.2	9.7		25.2	25.0		41.4	41.7		29.0	32.5
Partial		90.8	90.3		52.4	53.9		58.6	58.3		71.0	67.6
Food insecurity												
No		93.8	93.9		88.1	88.9		79.5	81.0		92.0	92.4
Yes		6.2	6.1		11.9	11.1		20.5	19.0		8.0	7.6
Education												
None/primary	77.6	75.7	77.7	49.7	47.5	49.2	7.8	6.7	7.8	11.8	11.5	12.1
Secondary low	16.4	17.3	16.1	2.1	1.6	2.0	72.9	75.1	72.9	51.2	52.8	51.3
Secondary high+	6.1	7.0	6.2	48.2	50.8	48.8	19.3	18.2	19.3	37.0	35.7	36.6
Contraception												
None	67.7	70.1	68.5	50.4	52.2	52.2	57.1	55.4	55.1	60.2	60.6	60.2
Less effective	7.8	5.7	5.8	9.7	7.2	7.2	31.3	32.7	32.3	27.1	25.8	25.6
Effective	10.3	9.9	10.4	20.5	19.2	19.6	4.3	4.2	10.4	6.2	6.1	6.7
Highly effective	14.0	14.3	15.3	19.4	21.4	21.2	7.3	7.8	15.3	6.5	7.5	7.5

± Less effective=barrier or natural methods; effective= short-acting hormonal methods, including pills, injectables, patches, or rings; highly effective=IUDs, implants, sterilization

For peer review only

STROBE Statement—Checklist of items that should be included in reports of ***cross-sectional studies***

	Item No	Recommendation	Pg. & Line
**Title and abstract**	1	(*a*) Indicate the study's design with a commonly used term in the title or the abstract	1
		(*b*) Provide in the abstract an informative and balanced summary of what was done and what was found	3
**Introduction**			
Background/rationale	2	Explain the scientific background and rationale for the investigation being reported	4
Objectives	3	State specific objectives, including any prespecified hypotheses	4
**Methods**			
Study design	4	Present key elements of study design early in the paper	4
Setting	5	Describe the setting, locations, and relevant dates, including periods of recruitment, exposure, follow-up, and data collection	4
Participants	6	(*a*) Give the eligibility criteria, and the sources and methods of selection of participants	5
Variables	7	Clearly define all outcomes, exposures, predictors, potential confounders, and effect modifiers. Give diagnostic criteria, if applicable	6-7
Data sources/ measurement	8*	For each variable of interest, give sources of data and details of methods of assessment (measurement). Describe comparability of assessment methods if there is more than one group	*6-7*
Bias	9	Describe any efforts to address potential sources of bias	7
Study size	10	Explain how the study size was arrived at	4
Quantitative variables	11	Explain how quantitative variables were handled in the analyses. If applicable, describe which groupings were chosen and why	7
Statistical methods	12	(*a*) Describe all statistical methods, including those used to control for confounding	7
		(*b*) Describe any methods used to examine subgroups and interactions	7
		(*c*) Explain how missing data were addressed	
		(*d*) If applicable, describe analytical methods taking account of sampling strategy	7
		(*e*) Describe any sensitivity analyses	
**Results**			
Participants	13*	(a) Report numbers of individuals at each stage of study—eg numbers potentially eligible, examined for eligibility, confirmed eligible, included in the study, completing follow-up, and analysed	7-8
		(b) Give reasons for non-participation at each stage	6
		(c) Consider use of a flow diagram	
Descriptive data	14*	(a) Give characteristics of study participants (eg demographic, clinical, social) and information on exposures and potential confounders	8
		(b) Indicate number of participants with missing data for each variable of interest	9- 10
Outcome data	15*	Report numbers of outcome events or summary measures	10

For peer review only

Main results	16	(*a*) Give unadjusted estimates and, if applicable, confounder-adjusted estimates and their precision (eg, 95% confidence interval). Make clear which confounders were adjusted for and why they were included	11-12
		(*b*) Report category boundaries when continuous variables were categorized	
		(*c*) If relevant, consider translating estimates of relative risk into absolute risk for a meaningful time period	
Other analyses	17	Report other analyses done—eg analyses of subgroups and interactions, and sensitivity analyses	
**Discussion**			
Key results	18	Summarise key results with reference to study objectives	12
Limitations	19	Discuss limitations of the study, taking into account sources of potential bias or imprecision. Discuss both direction and magnitude of any potential bias	13
Interpretation	20	Give a cautious overall interpretation of results considering objectives, limitations, multiplicity of analyses, results from similar studies, and other relevant evidence	12-13
Generalisability	21	Discuss the generalisability (external validity) of the study results	13
**Other information**			
Funding	22	Give the source of funding and the role of the funders for the present study and, if applicable, for the original study on which the present article is based	1

*Give information separately for exposed and unexposed groups.

Note: An Explanation and Elaboration article discusses each checklist item and gives methodological background and published examples of transparent reporting. The STROBE checklist is best used in conjunction with this article (freely available on the Web sites of PLoS Medicine at http://www.plosmedicine.org/, Annals of Internal Medicine at http://www.annals.org/, and Epidemiology at http://www.epidem.com/). Information on the STROBE Initiative is available at www.strobe-statement.org.

For peer review only
